# Role of Jasmonates, Calcium, and Glutathione in Plants to Combat Abiotic Stresses Through Precise Signaling Cascade

**DOI:** 10.3389/fpls.2021.668029

**Published:** 2021-07-22

**Authors:** Saima Aslam, Nadia Gul, Mudasir A. Mir, Mohd. Asgher, Nadiah Al-Sulami, Aala A. Abulfaraj, Sameer Qari

**Affiliations:** ^1^Department of Biotechnology, School of Biosciences and Biotechnology, Baba Ghulam Shah Badshah University, Rajouri, India; ^2^Division of Plant Biotechnology, Sher-e-Kashmir University of Agricultural Sciences and Technology of Kashmir (SKUAST-K), Srinagar, India; ^3^Department of Botany, School of Biosciences and Biotechnology, Baba Ghulam Shah Badshah University, Rajouri, India; ^4^Department of Biological Sciences, Faculty of Sciences, King Abdulaziz University, Jeddah, Saudi Arabia; ^5^Department of Biological Sciences, Science and Arts College, King Abdulaziz University, Jeddah, Saudi Arabia; ^6^Genetics and Molecular Biology Central Laboratory (GMCL), Department of Biology, Aljumun University College, Umm Al-Qura University, Mecca, Saudi Arabia

**Keywords:** abiotic stress, cell signaling, jasmonic acid, glutathione, calcium

## Abstract

Plant growth regulators have an important role in various developmental processes during the life cycle of plants. They are involved in abiotic stress responses and tolerance. They have very well-developed capabilities to sense the changes in their external milieu and initiate an appropriate signaling cascade that leads to the activation of plant defense mechanisms. The plant defense system activation causes build-up of plant defense hormones like jasmonic acid (JA) and antioxidant systems like glutathione (GSH). Moreover, calcium (Ca^2+^) transients are also seen during abiotic stress conditions depicting the role of Ca^2+^ in alleviating abiotic stress as well. Therefore, these growth regulators tend to control plant growth under varying abiotic stresses by regulating its oxidative defense and detoxification system. This review highlights the role of Jasmonates, Calcium, and glutathione in abiotic stress tolerance and activation of possible novel interlinked signaling cascade between them. Further, phyto-hormone crosstalk with jasmonates, calcium and glutathione under abiotic stress conditions followed by brief insights on omics approaches is also elucidated.

## Introduction

Different environmental conditions turn out to be the cause of stress in plants that tend to affect their growth, development, metabolism, and even cause death (Boguszewska and Zagdańska, 2012). These abiotic stresses such as salinity, heavy metals, temperature, drought, etc. are serious threats that affect crop productivity (Asgher et al., 2015; Raza et al., 2020). Plants have mechanisms due to which they adapt themselves to different climatic conditions by modulating their growth and physiology. Phytohormones are associated with various physiological and metabolic processes in plants (Kumar et al., 2014; Asgher et al., 2018; Geetika et al., 2020). The phytohormones play notable roles in inducing the numerous complex processes of growth, development, and response to stress by retaliating the signaling cascades in plants. Moreover, it has been suggested that these phytohormones have potential to minimize the ill effects of abiotic stress (Thao et al., 2015; Asgher et al., 2018; Zaid and Mohammad, 2018). Plant growth hormones such as auxin, gibberellin, cytokinin, abscisic acid, salicylic acid (SA), ethylene, JA, and recently studied brassinosteroid, act as components of abiotic-stress signaling (Fahad et al., 2015; Sharma and Laxmi, 2016; Wani et al., 2016). Among these phytohormones JA has gained much importance during the recent years.

JA has a ubiquitous expression in the plant systems. JA and its derivatives do have remarkable roles as plant growth and stress regulators, involved in diverse plant developmental processes such as callus growth, seed germination flowering, primary root growth, and senescence (Fahad et al., 2015). It acts as an important signaling molecule either in biotic or abiotic stress response (Wasternack, 2015; Per et al., 2018; Ali and Baek, 2020; Jang et al., 2020). Some jasmonates are derived from fungus while its methyl ester form, i.e., methyl jasmonate (MeJA) is extracted from petals of jasmine (*Jasminum grandiflorum*) (Avanci et al., 2010). It is usually present in flowers and reproductive tissues, while sparsely present in minute levels in root and mature leaves. JA have the capability to enhance or suppress the plant response (Agrawal et al., 2003; Fahad et al., 2015). JA tends to boost the antioxidant machinery of the plants (Bali et al., 2020).

Ca^2+^ acts as one of the important secondary messengers in all life forms involving many cell signaling cascades (Berridge et al., 2000; Stael et al., 2012). Among the most important nutrient elements, Ca^2+^ has a role under optimal and stressful conditions in plants (White and Broadley, 2003). Different kinds of stimulus are perceived by Ca^2+^ for downstream cellular retaliations via activation of Ca^2+^ channels followed by an increase in Ca^2+^ concentration due to influx of Ca^2+^ thereby inducing Ca^2+^ signaling (Evans et al., 2001; Chinnusamy et al., 2004). During abiotic stresses, Ca^2+^ signaling plays an important role by stimulation of Ca^2+^ channels and causes an increase in cytoplasmic Ca^2+^ levels for further downstream retaliations (McAinsh and Pittman, 2009; Dodd et al., 2010; Sarwat et al., 2013; Liu et al., 2018).

Glutathione one of the non-protein tripeptide thiol compounds, known as “master antioxidant” or “super defender,” is ubiquitous in nature and present in all plant cells at relatively high concentrations (Dixon et al., 1998). GSH is known to play a pivotal role in root development, plant disease resistance, protection against chilling damage, cell proliferation, and salt tolerance (Mittova et al., 2003; Gómez et al., 2004; Vivancos et al., 2010). GSH holds a very important position in stress responses by determining the cell redox state of the cell (Noctor et al., 2012).

Recent findings suggest that JA has a prime role at the physiological and biochemical levels that is associated with the plant defense against abiotic stress. However, JA cannot work alone to alleviate abiotic stress but works in concord manner via various signaling cascades. Vast literature is available on role of JA and its crosstalk with other phytohormones under abiotic stress, but there is no literature that documents the role of Jasmonate, Calcium, and Glutathione in plants to combat Abiotic stresses through precise signaling cascade. So, this review exemplifies the role of Jasmonates, Calcium, and glutathione in abiotic stress tolerance and activation of possible novel interlinked signaling cascade between them. Further, phyto-hormone crosstalk with jasmonates, Calcium, and Glutathione under abiotic stress conditions following with brief insights on omics approaches is also discussed.

## Biosynthesis of Jasmonic Acid

**FIGURE 1:**
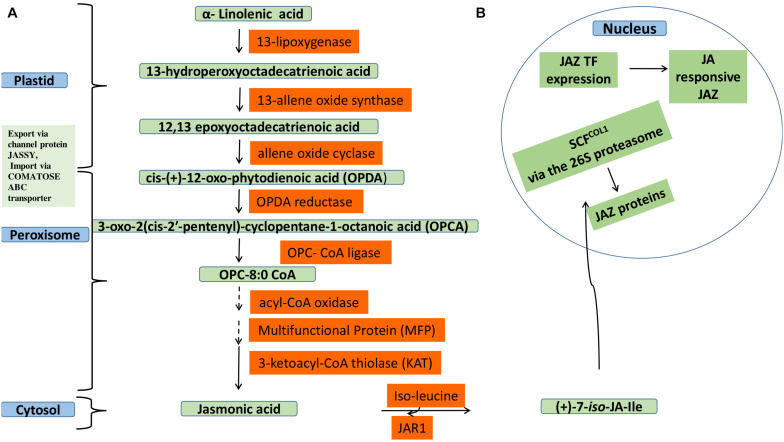
Jasmonic acid biosynthesis and its gene regulation. **(A)** Jasmonic acid biosynthesis in various cellular compartments involving plastid, peroxisome and cytosol. In plastid α-linolenic acid is converted into 12-oxo-phytodienoic acid that is exported via JASSY channel protein and imported via COMATOSE ABC transporter to peroxisome. Subsequent reduction, β-oxidation, and epimerization reactions lead to JA formation and is released into cytosol. Then JA Conjugates with isoleucine in cytosol to form jasmonoyl–isoleucine (Ile-JA). **(B)** Degradation of JAZ repressor proteins via SCF^*COI*1^ regulating JA gene expression at transcription level Ile-JA is in turn perceived Skip-Cullin–F-box complex (SCF^*COI*1^) which mediates degradation of the JAZ repressor via 26S proteasome degradation, thereby relieving the repression by JAZ, transcriptional regulator.

Jasmonic acid (JA) belongs to the family oxylipins, produced from polyunsaturated fatty acid (PUFA) through its oxidative metabolism (Wasternack and Hause, 2002; Ahmad et al., 2016). Its synthesis occurs via octadecanoic acid pathway involving esterification of α-linolenic acid (C18) in galactolipid membranes of chloroplast (Wasternack and Strnad, 2018; Wang et al., 2020). Phospholipase A causes the release of α- linolenic acid followed by oxygenation by 13-lipoxygenase (13-LOX) to a 13-hydroperoxyoctadecatrienoic acid, which is then converted by a 13-allene oxide synthase (13-AOS) to a highly unstable epoxide. Cyclization of this epoxide to *cis*-(+)-12-oxo-phytodienoic acid (OPDA) by the action of an allene oxide cyclase (AOC). The next half of JA biosynthesis takes place in peroxisomes. Followed by subsequent reduction and three steps of β-oxidation after which shortening of the carboxylic acid side chain to (+)-7-iso-JA, which is released into the cytosol and epimerizes to (−)JA. Then Conjugation with amino acids, such as isoleucine, is catalyzed by jasmonoyl–isoleucine (JA-ile) conjugate synthase (JAR1) which is the most active JA bio-compound (Wasternack and Hause, 2013; Ruan et al., 2019). JA and its metabolites, collectively called jasmonates, have an important role in mediating plant signaling in response to abiotic stress. JA signaling based gene expression is negatively regulated by jasmonate zip domain proteins (JAZ proteins). However, JA preferentially conjugates to isoleucine (Ile) to form Ile-JA or gets converted into MeJA (Wasternack, 2007; Wasternack and Hause, 2013). Ile-JA is in turn perceived Skip-Cullin–F-box complex (SCF^*COI*1^) which mediates degradation of the JAZ repressor via 26S proteasome degradation, thereby relieving the repression by JAZ, transcriptional regulator (Figure 1) (Melotto et al., 2008; Gfeller et al., 2010; Zhou and Memelink, 2016). The JAZ repressors recruit the protein topless (TPL) and the novel interactor of JAZ (NINJA) together to form transcriptional repression complex that inhibits the expression of jasmonate-responsive genes through formation closed to open complex, thereby favoring further attachment of histone deacetylase 6 (HDA6) and HDA19 (Causier et al., 2012; Chini et al., 2016).

## Biosynthesis of Glutathione

**FIGURE 2:**
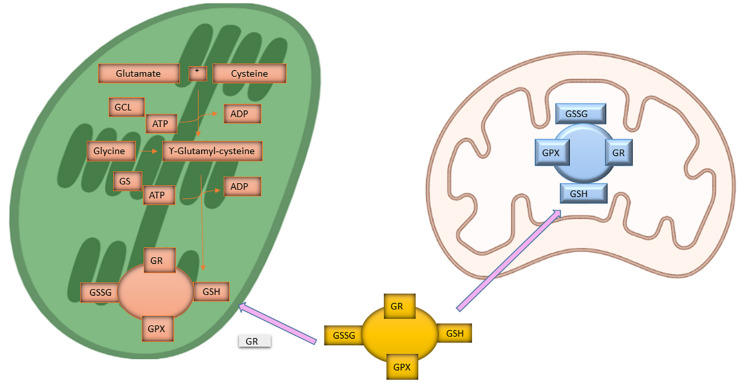
Biosynthesis of Glutathione. The two ATP dependent steps involved in biosynthesis of Glutathione. Glutamate and cysteine leads to the formation of γ-glutamylcysteine and in the next step glutathione formation occurs by addition of glycine to γ-glutamylcysteine. This reduced glutathione (GSH) acts as substrate for numerous cellular reactions in cytosol and mitochondria to yield oxidized glutathione (GSSG).

Various kinds of stresses tend to fluctuate the status of glutathione (Gómez et al., 2004). The biosynthesis of this non-protein thiol compound involves two ATP dependent steps as shown in Figure 2. First, the reaction is catalyzed by glutamate-cysteine ligase (GCL) involving glutamate and cysteine leading to the formation of γ-glutamylcysteine, and in the next step glutathione formation occurs by addition of glycine to γ-glutamylcysteine in the presence of glutathione synthetase (GS). This reduced glutathione (GSH) acts as substrate for numerous cellular reactions to yield oxidized glutathione (GSSG). The balance between GSH and GSSG acts as an important role in maintaining homeostasis of the cell (Meister, 1995; Noctor et al., 2012). Availability or localization of GCL and GS plays an important role in glutathione biosynthesis. Early reports suggest that in plant cells, GCL is localized in chloroplast and GS in cytosol as well as in chloroplast cells (Hell and Bergmann, 1988, 1990). Work on Arabidopsis suggest that GCL and GS are encoded by a single gene with alternate start sites leading either cytosolic or plastid targeted protein (Wachter et al., 2005). Glutathione biosynthesis compartmentalization is unique to plant systems (Galant et al., 2011; Noctor et al., 2012). Generally, over-expression of GCL, not GS, in plants raises glutathione content by increasing flux through the pathway. Increase in GCL activity that is from GSH to GSSG form takes place in response to 5 mM H_2_O_2_ treatment to Arabidopsis seedlings as depicted by immunoblot and activity assays (Hicks et al., 2007). Moreover, addition of cysteine, glutamate, or glycine does not enhance glutathione synthesis suggesting the role of GCL as a metabolic control point in the pathway (Meyer and Fricker, 2002). Metabolic studies suggest that feedback inhibition may not be a major control regulatory feature of glutathione (Meyer et al., 2001). Even though glutathione inhibits both GCL and GS (Jez et al., 2004). According to Previous reports glutathione biosynthesis pathway is controlled in a tightly regulated manner and hence supports the increased expression level of GCL and GS genes under various stress conditions (Xiang and Oliver, 1998; Lu, 2013).

## Role of Jasmonates, Calcium, and Glutathione in Abiotic Stress Tolerance

Abiotic stresses including heavy metals, salinity, temperature, drought, etc. represent a significant threat to plants by causing cellular damage and inhibiting normal physiological activities in plants thereby limiting productivity (Fujita et al., 2006; Asgher et al., 2015; Hasanuzzaman et al., 2017a) the detailed roles of JA, Ca^2+^, and GSH under different abiotic stress conditions are discussed below.

### Salt Stress

Alkaline stress is one of the most important stresses, especially in arid and semi -arid environments, that affects crop productivity at a global level (Parvin et al., 2019). Alkaline salt contamination to agricultural soil has been predicted in the past few decades mostly in Asian countries (Paz et al., 2012). Extreme alkaline stress can promote negative effects on plants at cellular level high sodium (Na^+^) concentration, enhancing ionic stress (Chen et al., 2012). About 10% of the world’s cultivable land productivity is affected by salt and alkaline stress (Tanji, 2006). Salinity disturbs the allocation of minerals and membrane permeability. It decreases chlorophyll biosynthesis, metabolism of nitrogen, and carbon dioxide (Gupta et al., 2002; Kim et al., 2004). High salinity also causes the production of reactive oxygen species (ROS), hence leading to oxidative stress (Smirnoff, 1993). Application of plant growth regulators including phytohormones help to counter the different environmental stresses in plants. Earlier reports have shown that JA activates expression of α- linolenic acid metabolism genes which is a branch of JA biosynthesis (Wasternack, 2007). Moreover, it has been seen that JA accumulation occurs under salt stress in plant species like *Solanum lycopersicum* (Pedranzani et al., 2003). The exogenous application of JA alleviated salt-induced injury in other variety of plants like barley (Walia et al., 2007), rice (Kang et al., 2005), and wheat (Qiu et al., 2014). Exogenous supplementation of (60 and 120 mM) MeJA increased growth and physiological attributes of *Anchusa italica* (Taheri et al., 2020). It has been recently reported that 45–60 μM MeJA significantly improved SOD, GPX, APX while 15–30 μM increased AsA, CAT, GSH activities in *Glycyrrhiza uralensis* seedlings under salt-stressed conditions, respectively (Lang et al., 2020). JA treatment led to up-regulation of the osmolyte synthesis, antioxidant system, and metabolite accumulation in tomato (Ahmad et al., 2018). Under increasing salt stress conditions abscisic acid (ABA) accumulated in tolerant varieties of rice while decreased in salt intolerant ones. Exogenous application of JA, however, led to an increase in ABA, especially in salt intolerant cultivars of rice, thereby ameliorating the salt stress (Kang et al., 2005). Exogenous application of MeJA effectively safeguards salinity stress symptoms in soybean seedlings by increasing the levels of ABA and relieving the repression of GA biosynthesis (Yoon et al., 2009). The lipoxygenase 3 (LOX 3) enzyme involved in JA biosynthesis of Arabidopsis is induced under salt treatment. However, LOX3 mutant seem to be hypersensitive toward salt treatment and could be complemented by exogenous JA treatment (Ding H. et al., 2016). Moreover, JA increased GSH related gene expression in plants in response to salt stress which in turn increases the antioxidant ability thereby protecting against oxidative stress caused by salt stress as seen in wheat seedlings (Qiu et al., 2014; Mir et al., 2018a). Ion homeostasis seem to be an important factor for plants under salt stress (Hasegawa, 2013). Plants have mechanisms by which they are able to sense salt stress via ionic and osmotic signals (Zhu, 2003). The Salt Overly Sensitive (SOS) pathway is a central mechanism in plant salt tolerance, which includes two calcium sensor proteins, SOS3 and SCaBP8; the protein kinase SOS2; and the Na^+^/H^+^ anti-porter SOS1. AtANNEXINs are a family of calcium-dependent membrane-binding proteins in plants. AtANNEXIN4 (AtANN4) plays a critical role in generating the calcium signal under salt stress that activates the SOS pathway in Arabidopsis. The SOS pathway suppresses AtANN4-mediated calcium transients through an interaction between SCaBP8 and AtANN4. AtANN4 likely functions during a very early stage of the plant salt stress response by generating a calcium signal. Afterward, an initial calcium signal is created in cells by AtANN4, SCaBP8, and SOS2 under salt stimulus. Phosphorylation of AtANN4 by SOS2 reduces the calcium-binding capability of AtANN4, which might alter calcium perception. The collaboration with SCaBP8 and phosphorylation by SOS2 represses the AtANN4-mediated calcium changes, there by provides a negative regulation loop. The negative feedback regulatory loop involving the SOS pathway decreases cytoplasmic sodium levels and elicits specific, long lasting salt stress reactions in plants (Ma L. et al., 2019). Phosphatases like calcineurin (protein phosphatase B) are important Ca^2+^ sensors and result in a decrease in K^+^ current and stomatal closure in plants. The specific to salt stress (SOS3) gene of Arabidopsis was comprehended to encode calcineurin B homolog of yeast. However, SOS3 mutant acts as a Ca^2+^ sensor to elicit downstream signaling under salt stress conditions which are hypersensitive to Na^+^ and are partially suppressed by increasing the concentration of Ca^2+^. Therefore, SOS3 mutant helps to unravel the basic role of Ca^2+^ in regulating potassium nutrition and salt stress in plants (Sanders et al., 1999).

### Metal(loid) Stress

Metal contamination of agricultural systems has become a worldwide concern due to industrialization and anthropogenic activities like mining (Chary et al., 2008; Noriega et al., 2012). Essential micronutrients like Zn and Cu are required by plants to carry out activities of different metal dependent proteins and enzymes. There accumulation above certain threshold value results in oxidative stress and nutrition stress. It results in alteration in carbohydrate metabolism, inhibition of photosynthesis and transpiration. Changes in plant morphology and physiology are also evident under heavy metal (HM) stress (Qureshi et al., 2016). JA production in response to metal or metalloid has been reported in *Wolffia arrhiza* (Piotrowska et al., 2009), *Arabidopsis thaliana* (Maksymiec et al., 2005), and *Cajanus cajan* (Poonam et al., 2013). Under biotic stress, JA levels increase within seconds to minutes (Chung et al., 2008). The endogenous JA levels also increase after abiotic stress exposure (Wang et al., 2020). However, response of exogenous application of JAs depends on the type of plant species tested or its concentration (Ahmad et al., 2016). Similarly, exogenous application of JA helps in alleviating the toxic effect of HM, depending on the species and the concentration of JA used. JA tends to modulate the HM accumulation by preventing its biosorption, plant growth restoration, and primary metabolite formation (Piotrowska et al., 2009; Farooq et al., 2016). GSH is also reported to have an important role in ROS detoxification and HM chelation. GSH has a central role in both antioxidant defense system and glyoxalase system and therefore provides protection from oxidative damage induced by HM. Moreover, GSH and its metabolizing enzymes such as glutathione-*s-*transferase (GST), glutathione peroxidase (GPX), glutathione reductase (GR), etc. provides protection against ROS by HM uptake, chelation, and detoxification. GSH also acts as cofactor in ROS scavenger reactions like in the glyoxalase pathway. Proline levels also increase in HM tolerance by maintaining the stringent redox environment of the cell by retaining a higher GSH pool (Hossain et al., 2012). Moreover, exogenous Ca^2+^ treatment increased the tolerance of the plant to HM by inhibiting ion uptake, increasing membrane stabilization, proline, and soluble sugar content (Shen et al., 1998; Kanu et al., 2019; Mukta et al., 2019).

#### Lead Stress

Lead (Pb) a non-essential element toxic to plants is absorbed by plants via roots, shoots, or foliage. Its entry into the plant cell causes inhibition of enzymatic activities, alteration of mineral nutrition, hormonal status, membrane structure, etc., leading to decreased growth and chlorosis (Seregin and Ivanov, 2001; Küpper, 2017). Change in enzyme activities causes inhibition of seed germination as reported in *Sporobolus alterniflorus* (Mrozek and Funicelli, 1982) and *Eichhornia crassipes* (Malar et al., 2016). Moreover, it is also responsible for inhibition of growth in plants like privet (Zhou et al., 2018). High Pb content leads to generation of ROS that directly or indirectly induces oxidative stress (Verma and Dubey, 2003; Reddy et al., 2005). JA elicits lead detoxification in tomato through production of secondary metabolites and gene expression and by decreasing the expression of the RBO and P-type ATPase transporter genes (Bali et al., 2019a, b). It also leads to the induction of lipid peroxidation, perturbing the level of saturated to unsaturated fatty acids in plant (Verma and Dubey, 2003; Bidar et al., 2008). Pb toxicity in *W. arrhiza* increased when supplemented with 100 μM of JA by the formation of lipid peroxides which resulted in decreased fresh weight, chlorophyll a, carotenoid soluble protein content, and monosaccharide while 0.1 μM of JA protected *W. arrhiza* fern against Pb stress by preventing Pb accumulation, restoring plant growth, and primary metabolite level by promoting the activities enzymatic antioxidants and non-enzymatic antioxidants, such as the content of AsA and GSH (Piotrowska et al., 2009). JA also causes changes in ascorbate glutathione pathway in plants like *Lycopersicum esculentum* under lead stress at various growth stages (Bali et al., 2018). GSH has chelating properties for heavy metals and therefore helps in ROS detoxification (Hossain et al., 2012). GSH also exhibits diverse functional roles in alleviating Pb induced toxicity by increasing activity of antioxidant enzymes like activating GPX and GR that act as ROS scavenger and regeneration of the GSH/GSSG pool of the cell, respectively (Hasanuzzaman et al., 2018). Exogenous application of GSH improved tolerance in *Iris lacteal* var. *chinensis* by mediating Pb accumulation and transport (Yuan et al., 2015).

#### Cadmium Stress

Cadmium (Cd), a non-redox heavy metal with long biological perseverance is highly toxic to plants (Asgher et al., 2015). It interferes with normal functioning of plants like photosynthesis, mineral, and water uptake (Baryla et al., 2001; Khan N.A. et al., 2016). Its toxicity in plants causes chlorosis, leaf rolling, and reduced growth of stem and root (Smeets et al., 2005; Mishra et al., 2006). It induces oxidative stress via generation of ROS causing serious damage to plants (Gallego et al., 2012). JA positively regulates plants in response to Cd stress (Zhao et al., 2016). Varying concentrations of JA and MeJA tend to alleviate the stress caused by Cd in soybean and *Oryza sativa.* 5 μM of MeJA improved antioxidant response and accumulation of antioxidants under Cd stress in *O. sativa* while 20 μM of JA reduced the damage caused by Pb stress in soybean (Noriega et al., 2012; Singh and Shah, 2014). These phytohormones cause increase in growth and photosynthesis besides changing the activity of different antioxidants and increasing GSH pools (Yan et al., 2013; Singh and Shah, 2014). Tomato seedlings susceptible to Cd show enhanced JA deficiency, this suggests that JA positively regulates the tomato plant to Cd stress (Zhao et al., 2016). Furthermore, JA acts as a signaling molecule for combating Cd stress and is also associated with expression of genes related to GSH biosynthesis (Maksymiec et al., 2007). Heavy metals compete with Ca^2+^ on the plasma membrane by substituting Cd, thereby altering the plant metabolism (Mansour, 2004). However, exogenous application of Ca^2+^ results in improving biochemical and physiological processes, besides enhancing activity of antioxidant enzymes, which provides tolerance against Cd stress as shown in faba bean (Siddiqui et al., 2012). Up regulation of antioxidant enzymes has been observed upon Ca^2+^ treatment to the *Sesamum indicum* under Cd stress (Abd-Allah et al., 2017). The same positive correlation was observed by exogenously applying GSH in combating Cd induced stress in *O. sativa.* It was reported that the difference in tolerance capability of sensitive and insensitive cultivars of *O. sativa* is associated with the tendency of the plant to elevate its GSH levels. Higher GSH levels halt the translocation of Cd and decreases its lethal effect. GSH treatment also enhances the chlorophyll level, photosynthetic performance and antioxidant capability of plants (Cai et al., 2011a; Fang et al., 2020).

#### Copper Stress

Copper (Cu^2+^) is a micronutrient that plays an important role in energy generation by means of ATP synthesis and carbon dioxide assimilation. It also alters ultra-structure and pigment composition of chloroplast. Therefore, being responsible for decline in the rate of photosynthesis via decrease in Ribulose-1, 5-bisphosphate carboxylase oxygenase (RuBisCO) inhibition of the electron transport chain and Photosystem II activities (Rakwal et al., 1996; Gang et al., 2013). High levels of Cu^2+^ exposure to *Theobroma cacao* seedlings caused damaging effects such as absence of starch grains and swelling of chloroplast double membrane (Souza et al., 2014). It has been reported that JA (1 μM, 1 nM, and 1 pM) enhances photosynthetic pigment accumulation and production of hydrogen peroxide (H_2_O_2_) mitigating enzymes, i.e., superoxide dismutase (SOD) and peroxidase (POD), suggesting that seed priming with JA can decrease the toxic effect of Cu^2+^ (Poonam et al., 2013). Moreover, addition of Ca^2+^ into nutrient solution improved the growth of Cu-treated seedling, by lowering the concentration of polyamines putrescine and increasing the levels of spermine and spermidine in the epicotyl of plants (Shen et al., 1998). Supplementation of Ca^2+^ to pea plants increases the Cu metal bio- absorption and maintains the homeostatic environment of the cell (Ben Massoud et al., 2019). A similar effect has been observed for GSH that helps in alleviating the effect of copper in rice seedlings by reducing copper uptake (Mostofa et al., 2015). Pretreatment with GSH caused the activation of oxidative stress scavenging mechanisms of plant thereby decreasing the level of ROS and Malondialdehyde concentration (Tahjib-Ul-Arif et al., 2020).

#### Arsenic Stress

Arsenic toxicity poses a serious health threat to all living organisms across the globe associated with anthropogenic activities like mining and smelting operations (Kumar et al., 2015; Singh et al., 2015). The As contamination in groundwater is a worldwide problem. It badly affects crop productivity and accumulates in different plant tissues, including grains, and affects the food chain (Verma S. et al., 2016; Ghosh et al., 2019). Naturally As exists in Inorganic arsenate As (V) and arsenite As (III) forms. Both forms are toxic but As (III) is more toxic than As (V) to plants as it has a tendency to bind proteins with sulfhydryl groups and hinder with their functions (Verma S. et al., 2016). It also leads to ROS generation and inhibition of respiration by binding to vicinal thiols in pyruvate dehydrogenase and 2-oxo-glutarate dehydrogenase (Helleday et al., 2000; Verma S. et al., 2016). Thus, arsenic-induced ROS production causes impairment of normal cellular function and plant metabolism by widespread damage to DNA, lipids, and proteins (de Campos et al., 2019). In order to recognize the possible mechanism involved in mitigating As toxicity in plants, the expression analysis of various genes involved in AsIII translocation and sequestration have been analyzed. MeJA has been reported to alleviate AsIII toxicity in rice through modulating As uptake, translocation, and JA signaling (Verma et al., 2020). JA also regulates the pigment balance, ROS homeostasis, and improvement of the antioxidant enzymatic system, thereby increasing accumulation of As without showing major damage (Coelho et al., 2020). It has also been found that MeJA has improved the growth and yield characteristics of rice varieties under As toxicity by alleviating oxidative stress through increasing the activity of antioxidant enzymes along with ASA–GSH cycle and reducing As accumulation by controlling As transporters (Mousavi et al., 2020). As induces the generation of ROS leading to oxidative stress and lipid peroxidation in plants (Shukla et al., 2018). Arsenic toxicity causes activation of phytochelatins (PCs) produced from GSH. PCs sequester As into the vacuoles make complexes with As, which gets sequestered into the vacuoles through ABCC1/ABCC2 transporters (Schmöger et al., 2000; Dhankher, 2005). Furthermore, Lambda class of GST (GSTLs) has been seen to bind tightly to the flavonols and their derivatives (Chronopoulou et al., 2017). It has been suggested that GSTLs can recycle GSH adducts of oxidized flavonols back to the parent flavonols, maintaining the antioxidant pools (Hernández et al., 2009). Genome wide expression analyses have shown differential expression of OsGSTLs at various stages of plant development as well as under stress conditions (Kumar et al., 2013). However, the exogenous use of GSH in As-treated seedlings decreased As-induced oxidative stress, increased the AsA and GSH contents, and mediated As translocation from the roots to the shoots. Therefore, the results suggest that exogenous GSH application could be a favorable approach to enhance As stress resistance in rice (Farooq et al., 2018; Jung et al., 2019). The identification of calcium-dependent protein kinase CPK31 is a major component controlling As(III) tolerance in Arabidopsis. Genetic and biochemical studies show that CPK31 fulfils this function by interaction with NIP1;1, providing a novel role of CPK31 in controlling As(III) toxicity in plants via Ca^2+^ signaling (Mousavi et al., 2020).

#### Nickel Stress

Nickel (Ni) is among the common heavy metals that cause serious health complications even in trace quantity (Masindi and Muedi, 2018). Nickel induces the deficiency of Zn and Fe. It also hinders the uptake of other heavy metals such as Cd, Pb, Co, and Cr (Myśliwa-Kurdziel et al., 2004). Nickel toxicity disrupts the important macro and micronutrients uptake by hindering the translocation of these nutrients through root to aerial (Pandey and Sharma, 2002; Chen et al., 2009; Ameen et al., 2019). The treatment of NiSO4.7H2O has led to decrease in chloroplast size and number. It was also seem to be responsible for the disorganization of ultrastructure of chloroplast like numbers of grana decreased, thylakoids deformation, the development of plasto globuli, and the membrane lipid composition alterations were stated in *Brassica oleracea* plants. These changes were due to the Ni induced drop in cell moisture content or subsequent peroxidation of membrane lipids due to oxidative stress (Ameen et al., 2019). The toxicity of Ni has been associated with oxidative stress in plants (Rao and Sresty, 2000; Boominathan and Doran, 2002). Nickel toxicity like other abiotic stresses led to production of ROS (Gill and Tuteja, 2010). Excessive Ni increases the concentration of superoxide anions, hydroxyl radicals, nitric oxide, and hydrogen peroxide (Stohs et al., 2000). The toxicity of heavy metals is directly linked with overall crop yield. The increasing concentration of Ni has deleterious effects on plants that finally triggered reduction in crop yield (Balaguer et al., 1993). Ni was also reported to be associated with inhibition of germination and production of chlorophyll (Zhou et al., 2009). Exogenous application of JA relieved the adverse effect of oxidative stress on biomass production, growth, and protein content in Ni treated plants by further enhancing the activity of antioxidant enzymes (Azeem, 2018; Mir et al., 2018b). However, exogenous or endogenous biosynthesis of JA make plants tolerant to any oxidative damage (Sirhindi et al., 2016). There is information that methyl ester of JA (MeJA) affects the pools of stress antioxidant enzymes activity to combat oxidative stress (Jung, 2004). Ni stimulated the activities of SOD, APX, and CAT. SOD is the primary enzyme of Asada-Halliwell pathway that causes dismutation of superoxide radicals under elevated levels of H2O2 followed by further hydrolysis into H2O and O2 by CAT and APOX or other POD enzymes present in various cellular organelles (Keramat et al., 2010; Sirhindi et al., 2015). Higher GSH reductase and catalase activities are present in a Ni-tolerant strain of the green alga, *Scenedesmus acutus f. alternans* (Randhawa et al., 2001). Also in transgenic Arabidopsis, GSH seems to be intensely associated with increased resistance the growth inhibitory and oxidative stress induced effects of Ni. This rise in GSH concentrations was reported to be determined by serine acetyltransferase (SAT) activity in conferring tolerance to Ni-induced oxidative stress in Thlaspi Ni hyperaccumulators (Freeman et al., 2004). However, the application of Ca^2+^ has been seen to be responsible for the higher efficiency of the antioxidants for increasing tomato tolerance to the Ni stress (Asrar et al., 2014).

### Drought Stress

Drought is responsible for great famines of the past. It is one of the common threats to food security. Limited supply and increasing demand of water worsened drought effects (Somerville and Briscoe, 2001). Jasmonate zip-domain (JAZ) proteins are essential regulators of proteins of JA signaling in many plants including *A. thaliana* (Vanholme et al., 2007) and rice (Ye et al., 2009). It has been reported that enhanced expression of stress responsive OsJAZ1- gene of *O. sativa* showed higher sensitivity to drought stress, while the JAZ1 mutant plants were more hyposensitive to drought stress compared to wild plants, suggesting the role of JA in combating the drought stress in plants (Fu et al., 2017). Exogenous application of MeJA (75, 150, and 225 μM) improved many characteristics of *Satureja hortensis* such as growth, water content, proline level, antioxidant activity, and essential oil percentage as well as yield. However, among different concentrations used, 75 μM was more effective, like that seen in drought tolerance in different *Brassica* species by trehalose (5 mM) treatment (Hasanuzzaman et al., 2014; Miranshahi and Sayyari, 2016). Exogenously applied 0.1 μM of MeJA to the wheat seedlings decreased the drought-induced retarded growth, lesions of membrane by increasing the level of dehydrin protein expression in them (Allagulova et al., 2020). JA treatment to cowpea plant under drought stress improves relative water content, proline, chlorophyll content, and causes stomatal closure, so as to elevate the stress pertaining to drought (Sadeghipour, 2018; Tayyab et al., 2020). Exogenous application of 10 and 150 μM JA increases the antioxidant potential of sugar beet (Ghaffari et al., 2019) and bitter melon (Alisofi et al., 2020) respectively, under drought conditions thereby imparting tolerance to them. Differential expression of Me-JA induced miRNAs was seen by Me-JA treatment in wheat under drought stress. These miRNAs could play a significant role in the activation of a particular gene, so play an important function in combating drought stress (Ma C. et al., 2019). GSH also reduces the effect of drought stress via maintaining water status, proline content, and by acting as an antioxidant (Nahar et al., 2015a). Exogenous GSH application improves growth characteristics and yield of plants under drought stress (Chen et al., 2012; Nahar et al., 2015a). Recent studies indicate that apart from increasing antioxidant activity GSH has a role in maintaining the plant mineral homeostasis (Sohag et al., 2020). However, it has also been reported that application of Ca^2+^ and H_2_O_2_ to plants mitigated ill effects of the drought stress (Hasanuzzaman et al., 2014; Hu et al., 2018). Ca^2+^ also regulates the water status, proline, and H_2_O_2_ levels in maize plants under drought stress (Naeem et al., 2018).

### Flooding Stress

Flooding influences agricultural productivity all over world (Jackson and Colmer, 2005). It restricts gaseous exchange between plants and their environment, thereby resulting in lowering of oxygen, carbon dioxide levels, and increasing levels of ethylene in plants (Bailey-Serres and Voesenek, 2008). Its interference in photosynthesis and respiration in plants hinders production of ATP via oxidative phosphorylation, besides leading to generation of ROS due to hypoxic and anoxic conditions (Gibbs and Greenway, 2003; Paradiso et al., 2016). Owing to flooding stress, plants experience compound stress like energy and carbon deficiency that leads to retardation of plant growth (Armstrong, 1980; Jackson, 1985). JA upregulates the ROS and H_2_O_2_ detoxification system in plant cells during floods (Nanjo et al., 2011). JA is reported to have a post-flooding recovery function in soyabean by modulating the levels of nucleotidylyl transferase activity (Khan and Komatsu, 2016). Studies have shown that GSH regulates the gene expression of JA at a basal level. Ca^2+^ also has a role in regulating the cell wall integrity besides the mitigating effect of oxidative stress during flood conditions (Porto et al., 2013). Supplementation of GSH to rice plant increased its antioxidant potential and could be an important factor to rescue plants under flooding stress (Siddiqui et al., 2020). Exogenous application of Ca^2+^ causes the root elongation and inhibits the cell death at the root tips of soyabean under flood stress (Oh et al., 2014). Moreover, external supplementation of Ca^2+^ decreased the negative effects on their physiological parameters like stomatal conductance, photosynthesis, soluble protein content, fruit size, etc., and also seem to have roles in maintaining the integrity of root cells of pepper (Ou et al., 2017). In addition, early cytosolic Ca^2+^ transients also seem to be important in circumventing the effect of flood stress among plants (Subbaiah and Sachs, 2003).

### Ozone Stress

Stratospheric ozone layer depletion results in enhancement of the tropospheric ozone levels that adversely affect the terrestrial biosphere (Overmyer et al., 2000; Ainsworth et al., 2012). Ozone mediated changes at the cellular level in plants involves oxidative burst, accelerated cell senescence, and hypersensitive response kind of reactions (Vollenweider et al., 2003). Oxidative burst leads to lump and strand like protrusions on the cell wall with enhanced cellular oxidation (Günthardt-Goerg et al., 1997). ROS generation overcomes the cellular detoxification system. Its accumulation results in hypersensitive response with apparent symptoms like disruption of cellular structure, collapse of cell walls, incomplete degradation of cellular organelles, chromatin condensation, condensation of cell leftovers into apoptotic-like bodies, and nuclear degeneration leading to cell death (Vollenweider et al., 2003; Iriti and Faoro, 2007). JA plays a role in mitigating the ozone stress in plants (Tamaoki, 2008). JA insensitive mutants of Arabidopsis such as methyl-jasmonate resistant1 (jar1), coronatine insensitive1 (coi1), ozone-sensitive and jasmonate-insensitive (oji1), JA-biosynthesis defective fad3/7/8triple mutant, and the 12-oxophytodienoate reductase 3 (OPR3) mutants are extremely susceptible to ozone (Staswick et al., 1992; Feys et al., 1994; Overmyer et al., 2000; Kanna et al., 2004). O_3_ tolerant cultivar of wheat is reported to have increased expression of JA as compared to the non-tolerant variety, thus rendering it to alleviate the ozone stress (Fatima et al., 2018). JA was also found to be associated with the maintenance of cellular homeostasis under ozone stress in *Brassica campestris* (Zhang et al., 2017). Change in GSH/GSSG pools is also seen as an early symptom to ozone exposure (Tausz et al., 1999). Tobacco sensitive variety (9 BelW3) upon ozone fumigation showed oxidation of GSH pool with a decrease in GR activity while the resistant variety (BelB) showed a high GSH/GSSG ratio with increased GR activity (Pasqualini et al., 1999). However, recent genome-based expression profiling of *Glycine max* GST gene (GmGST) has reported the presence of 126 putative GST gene in *G. max* and, among them, four genes, namely GmGSTU63, GmGSTF2, GmGSTU73, and GmGSTT5 are highly expressed under few abiotic stresses including ozone stress, therefore providing tolerance against adverse climatic conditions (Hasan et al., 2020). Ca^2+^ transients are also seen in ozone stress conditions (Sanders et al., 2002). But in the presence of Ca^2+^ channel blockers like lanthanum chloride decreased glutathione-*s-*transferase (GST) expression takes place which in turn affects the GSH/GSSG pool. Also, Ca^2+^ dependent differential gene expression is observed in Arabidopsis under ozone stress thereby elucidating important role of Ca^2+^ as well in combating ozone mediated stress in plants (Clayton et al., 1999; Short et al., 2012).

### Temperature Stress

Extreme differential temperature exposure causes stress in plants. Temperature stress (cold/low) alters the normal functioning of plants (Dhingra, 2015; Hatfield and Prueger, 2015), JA has role in surmounting the effect of extreme temperatures in plants (Zhao et al., 2013). Role of JA, Ca^2+^, and GSH under extreme temperature conditions are discussed below.

#### Cold Stress

Cold (low) temperature stress, a major threat that prevents plants from resuming full potential, results in a decrease in the crop productivity worldwide (Yadav, 2010; Dhingra, 2015). It affects plant metabolism and growth via inhibition of electron transport chain and disturbance in the activity of enzymes that participate in plant metabolism (Dhingra, 2015). Low temperature exposure of a plant leads to oxidative stress. During which, the plant’s antioxidant machinery is activated to restore normal functioning of the plant. Antioxidants play key roles in cold acclimatization, low temperature stress tolerance, and maintenance of cellular redox homeostasis (Chen and Li, 2002; Khan et al., 2015). Exogenous application of MeJA to Arabidopsis (Hu et al., 2013) and loquat fruit (Cai et al., 2011b) imparted cold tolerance to them. However, it has been reported in cold tolerant *Camellia japonica* that upregulation of MYC – genes that are key regulators of JA signaling occurs in addition to an increase in the levels of precursor molecule α-linolenic acid of JA biosynthesis (Li Q. et al., 2016). The inducer of the CBF expression ICE-CBF pathway plays a core role in cold stress related response in plants. Under a normal set of conditions, ICE1 and ICE2 bind to the CANNTG sequence of the promoter region of CBF genes. These factors also bind JAZ1 and JAZ4, causing inhibition of ICE-CBF pathway. However, under cold stress conditions, the formation of more JA-Ile occurs which mediates the 26s proteasome degradation of JAZ factors that were previously bound to ICE1 and ICE2, hence activation of the ICE- CBF pathway occurs (Hu et al., 2017). It has been reported that external application of MeJA to a rubber tree eliminates the repression of JAZ proteins on ICE2 transcription factor that has an important role in the activation of CBF (C-repeat binding factor) cold signaling pathways involving genes CBF1, CBF2, COR47. So, the increase in the gene expression of CBF1, CBF2, COR47 genes tend to acclimate cold stress conditions in plants like rubber trees (Chen et al., 2019). Further, JA-related expression of genes involved in synthesis of GSH and GR occurs (Xiang and Oliver, 1998). It upregulates the antioxidant activities and protects the ultra-structure of the cell against cold stress (Li et al., 2012). Low temperatures increase the GSH level many folds which in turn alters the redox status of GSH (Wildi and Lütz, 1996; Karpinski et al., 1997). GSH accumulation was seen to be more evident in cold tolerant varieties of rice as compared to non-tolerant (Yu et al., 2020). Cold stress can inhibit some metabolic activity of plants (Shi Y. et al., 2018). Moreover, GSH interacts with JA, which is involved in regulating plant developmental processes and signaling networks under different types of stresses (Per et al., 2018). It has also been reported that Ca^2+^ influx is required for elicitor-induced synthesis of JA (Hu et al., 2009). Thus, JA induced signaling cascade may lead to activation of nifedipine sensitive channels associated with the increase in cytosolic Ca^2+^ through release from intra-cellular stores (Sun et al., 2009). The decrease in temperature also causes significant increase in the cellular Ca^2+^ through increase in the influx of Ca^2+^ ions. Increased influx of radio labeled Ca^2+^ was seen in roots of plants in response to cold stress by the hypo-polarization of plasmalemma (Rincon and Hanson, 1986). Exogenous Ca^2+^ enhanced the tolerance potential of wheat under cold stress by regulating the levels of antioxidant machinery, photosynthetic rate, and membrane injury (Zhang et al., 2020).

#### Heat Stress

Constant rise in temperature due to greenhouse gases emission causes heat stress in plants. Plants are worst hit because of their sessile nature which makes them unable to shift to better place to handle the damaging effect of heat (Cassia et al., 2018). Heat stress greatly affects growth, physiological aspects, development, and yield of plant, thereby leading to generation of ROS in excess eliciting oxidative stress (Hasanuzzaman et al., 2013; Sarwar et al., 2018). It has been reported that applying JA helps to mitigate the effects of heat stress in plants via activating the oxidative defense and detoxification system (Sharma and Laxmi, 2016). Heat induced inhibition of photosynthesis is counteracted by Ca^2+^ salts that ameliorate the damage to Photosystem II as observed in tomato (Sakhonwasee and Phingkasan, 2017) and tobacco (Tan et al., 2011). Ca^2+^ ions tend to decrease the level of ROS production (Sakhonwasee and Phingkasan, 2017). Exogenous application of Ca^2+^ led to thermos-tolerance in common bean by up-regulating antioxidant enzyme activity and sugar accumulation in them (Naeem et al., 2020). GSH has also been found to protect plants under heat stress via improving photosynthetic attributes, osmolytes, and antioxidant levels in plants such as in Arabidopsis (Cheng et al., 2015). External application of GSH imparted heat tolerance in plants as seen in *Cummis sativa* (Ding X. et al., 2016). However, the involvement of GSH in mitigating heat mediated oxidative stress in plant is very well documented (Nahar et al., 2015a).

The response of exogenously applied JA, Ca^2+^, and GSH, respectively, to different plant varieties under various kinds of abiotic stresses is given below in the Table 1.

**TABLE 1:** Abiotic stress response in relation with JA, Ca^2+^, and GSH among different plant species.

Stress type	Plant species		JA/GSH/Ca^2^^+^	Response	Growth pattern	Cotyledon number	References
	
			MeJA				
High salt concentration	*Pisum sativum* (L.)		10^–5^ M	Osmoregulation, increased proline content	Annual	Dicot	Fedina and Tsonev, 1997
	*Glycine max* (L.)		20 and 30 μM	Increase in growth and proline content	Annual	Dicot	Yoon et al., 2009
	*Arabidopsis thaliana* (L.)		5 and 10 μM	Compliments lox3 mutant rescues salt stress	Annual	Dicot	Ding H. et al., 2016
	*Triticum aestivum* (L.)		0.1 μM	Increases cytokinin production and plant growth	Annual	Monocot	Avalbaev et al., 2016
	*Solanum lycopersicum* (L.)		10, 20, 30, 40, 50, and 60 μM	Increase in levels of osmo-protectants and enzymatic antioxidants	Annual	Dicot	Manan et al., 2016
	*Brassica napus* (L.)		100 μM	Increases relative water content, soluble sugar, photosynthesis	Annual	Dicot	Ahmadi et al., 2018

			**JA**				

	*Pisum sativum* (L.)		10^–5^ M	Decreased activity of sodium and chloride ions, increased endogenous level of proline	Annual	Dicot	Velitchkova and Fedina, 1998
	*Oryza sativa* (L.)		30 μM	Increases ion uptake, growth, ABA levels	Annual	Monocot	Kang et al., 2005
	*Hordeum vulgare* (L.)		12 μM	Induction of genes having role in imparting salt tolerance	Annual	Monocot	Walia et al., 2007
	*Brassica napus* (L.)		10^–6^, 10^–9^, and 10^–12^ M	Sugar accumulation	Annual	Dicot	Kaur et al., 2013
	*Triticum aestivum* (L.)		2 mM	Increase in concentration of GSH, enhanced activity of SOD, CAT, APX	Annual	Monocot	Qiu et al., 2014

			**GSH**				

	*Oryza sativa* (L.)		2 mM	Positive influence on yield contributing traits	Annual	Monocot	Wang et al., 2014
	*Arabidopsis thaliana* (L.)		400 μM	Abscisic acid, auxin and jasmonic acid biosynthesis	Annual	Dicot	Cheng et al., 2015
	*Vigna radiata* (L.)		1 mM	Activation of glyoxalase system and improved antioxidant system	Annual	Dicot	Nahar et al., 2015b
	*Solanum lycopersicum* (L.)		5 mM	Increased GSH biosynthesis, improved activity of SOD, CAT, POD	Annual	Dicot	Zhou et al., 2017
	*Glycine max* (L.)		2 mM	Improved stress tolerance and yield attributes	Annual	Dicot	Akram et al., 2017

			**Ca^2^**^+^				

	*Solanum lycopersicum* (L.)		5 and 10 mM	Increased growth, physiology and fruit production	Annual	Dicot	Parvin et al., 2015
	*Glycine max* (L.)		6 mM	Positive effect on growth and metabolic activities.	Annual	Dicot	Yin et al., 2015
	*Oryza sativa* (L.)		3 and 5 mM	Elevated antioxidant enzyme levels	Annual	Monocot	Tahjib-Ul-Arif et al., 2018

	**Lead**		**JA**				

Heavy metal stresses	*Wolffia arrhiza* (L.)		0.1 μM	Preventing Pb accumulation by restoring plant growth and primary metabolite level	Perennial	Monocot	Piotrowska et al., 2009
	*Solanum lycopersicum* (L.)		0.1, 1, and 100 μM	Increase osmolytes concentration and ascorbate glutathione cycle	Annual	Dicot	Bali et al., 2018

				**GSH**			

	*Gossypium* sp. (L.)		50 μM	Stabilized ultra-structure and increased antioxidant activity	Perennial	Dicot	Khan M. et al., 2016
	*Triticum aestivum* (L.)		1 mM	Enhancement of enzymatic and non-enzymatic antioxidant activities and improved seedling growth	Annual	Monocot	Hasanuzzaman et al., 2018

	**Cadmium**		**JA**				

	*Glycine max* (L.)		20 μM	Increased antioxidant response	Annual	Dicot	Noriega et al., 2012
	*Vicia faba* (L.)		0.01 mM	Restoration of growth and pigment system	Annual	Dicot	Ahmad et al., 2017
	*Brassica napus* (L.)		25 μM	Osmolytes and antioxidant activity increased	Annual	Dicot	Ali et al., 2018

			**MeJA**				

	*Oryza sativa* (L.)		5 μM	GSH homeostasis, JA biosynthesis	Annual	Monocot	Singh and Shah, 2014
	*Arabidopsis thaliana* (L.)		0.01 μM	Suppression of genes involved in Cd uptake	Annual	Dicot	Lei et al., 2020
			**GSH**				
	*Hordeum vulgare* (L.)		20 mg/L	Improved photosynthesis	Annual	Monocot	Chen et al., 2010
	*Oryza sativa* (L.)		50 μM	Enhanced photosynthetic performance	Annual	Monocot	Cai et al., 2011a
	*Gossypium* sp. (L.)		50 μM	Reverses stressful effects, leaf ultra-morphology revived	Perennial	Monocot	Daud et al., 2016
	*Populus* sp. (L.)		100 μM	Increased Cd detoxifying gene transcript	Perennial	Monocot	Ding et al., 2017

			**Ca^2^**^+^				

	*Vicia faba* (L.)		2%	Antioxidant enzyme up regulation	Annual	Dicot	Siddiqui et al., 2012
	*Brassica juncea* (L.)		50 mM	Improved photosynthesis	Annual	Dicot	Ahmad et al., 2015
	*Arabidopsis thaliana* (L.)		3 mM	Alleviated the inhibition of Cd on the root growth	Annual	Dicot	Li P. et al., 2016
	*Sesamum indicum* (L.)		50 mM	Improved growth and proline levels	Annual	Dicot	Abd-Allah et al., 2017

	**Copper**		**JA**				

	*Oryza sativa* (L.)		0.5 mM	Phytoalexin production	Annual	Monocot	Rakwal et al., 1996
	*Cajanus cajan* (L.)		1 μM, 1 nM,	Osmolytes and antioxidant enzyme increased	Perennial	Dicot	Poonam et al., 2013
	*Triticum Aestivum* (L.)		5 mM	Increased transcript of glutathione–*s*- transferase	Annual	Monocot	Li et al., 2013

			**MeJA**				

	*Phaseolus coccineus* (L.)		10^–5^ M	Promoted plant growth and development	Perennial	Dicot	Hanaka et al., 2015

			**GSH**				

	*Triticum aestivum* (L.)		2.5 mM/L	Accumulation of nitrogen, sulfur, and phosphorous	Annual	Monocot	Peng et al., 2012
	*Glycine Max* (L.)		0.16 and 0.32 Mm/L	Enhances amylase activity	Annual	Dicot	Chen, 2012
	*Oryza Sativa* (L.)		100 mg/L	Increased germination rate and vigor index	Annual	Monocot	Mostofa et al., 2015

			**Ca^2^**^+^				

	**Drought**	**JA**					

Water stress	*Glycine Max* (L.)		4.5 and 9 mM/L	Maintenance of membrane integrity	Annual	Dicot	Chen et al., 2008
	*Vigna radiata* (L.)		5 mM	Solution improved the growth of Cu-treated seedling and lowering the concentration of Polyamines putrescine and increased concentrations of spermine and spermidine in epicotyl of plants	Annual	Dicot	Shen et al., 1998
	*Brassica* sp. (L.)		0.5 mM	Increase in physiological, antioxidant and glyoxalase system activities	Annual	Dicot	Alam et al., 2014
	*Allium cepa* (L.)		25, 50, and 100 μM	Pigment and compatible solute enhancement	Annual	Monocot	Ahmad and Murali, 2015
	*Beta vulgaris* (L.)		5 and 10 μM	Increased germination rate	Annual	Dicot	Ghafari and Tadayon, 2019

			**MeJA**				

	*Brassica oleracea* (L.)		10 μM	Increased Net photosynthetic rate and antioxidant machinery activation	Annual	Dicot	Wu et al., 2012
	*Triticum aestivum* (L.)		0.25 μM	Water status and antioxidant capacity increased	Annual	Monocot	Ma et al., 2014
	*Satureja hortensis* (L.)		75, 150, and 225 μM	Improved many characteristics of plant like growth, water content, proline level, antioxidant activity	Annual	Dicot	Miranshahi and Sayyari, 2016

			**GSH**				

	*Arabidopsis thaliana* (L.)		400 μM	Changes at translational level of numerous hormones	Annual	Dicot	Cheng et al., 2015
	*Vigna radiata* (L.)		1 mM	Improved their antioxidant components under drought stress	Annual	Dicot	Nahar et al., 2015a

			**Ca^2^**^+^				

	*Zoysia japonica* (L.)		5 and 10 mM	Improved photosynthesis, growth and antioxidant response	Perennial	Monocot	Xu et al., 2013
	*Zea mays* (L.)		5 mg/L	Improved photosynthesis, growth and soluble sugar content	Annual	Monocot	Naeem et al., 2018
	*Nicotiana tabacum* (L.)		10 mM/L	Stabilization of gaseous exchange and photosynthetic organelles	Annual	Dicot	Hu et al., 2018

	**Flooding**		**JA**				

	*Citrus* spp. (L.)		1 mM	Increase in abscisic acid levels	Perennial	Dicot	de Ollas et al., 2013

			**Ca^2^**^+^				

	*Zea mays* (L.)		0.75% (W/V)	Regulates the cell wall integrity and mitigates effect of oxidative stress during flood stress conditions	Annual	Monocot	Porto et al., 2013
	*Glycine max* (L.)		50 mM	Increase the root elongation and inhibited the cell death of root tip of under flood stress	Annual	Dicot	Oh et al., 2014

			**JA**				

Ozone stress	*Capsicum annuum* (L.)		10 mM	Regulates osmotic and antioxidant metabolism	Annual	Dicot	Yang et al., 2016
	*Arabidopsis thaliana* (L.)		1.4 μM	Inhibited cell death and lesion containment	Annual	Dicot	Overmyer et al., 2000
	*Arabidopsis thaliana* (L.) (JA insensitive mutants)		10 μM	Extremely susceptible to ozone	Annual	Dicot	Kanna et al., 2004

			**GSH**				

	Transgenic *Nicotiana tabacum* (L.)		Overexpression of glutathione synthetase in plastid	Ozone tolerance developed	Annual	Dicot	Wellburn et al., 1998
	*Populus* sp. (L.)		Overexpression of Glutathione reductase	Ozone tolerance developed	Perennial	Monocot	Foyer et al., 1995

	**Heat**		**JA**				

Temperature stress	*Vitis* sp (L.) seedling		50 μM/L	Thermotolerance	Perennial	Dicot	Chen et al., 2006

			**Ca^2^**^+^				

	*Solanum lycopersicum* (L.)		1 Mm	Operating efficiency of photosystem II increased	Annual	Dicot	Sakhonwasee and Phingkasan, 2017
	*Nicotiana tabacum* (L.)		20 Mm	Improved stomatal conductance and thermostablity	Annual	Dicot	Tan et al., 2011

	**Cold**		**JA**				

	*Prunus persica* (L.)		0.1 Mm/L	Maintenance of fruit quality	Perennial	Dicot	Meng et al., 2009

			**MeJA**				

	*Cucumis sativus* (L.)		100 μM	Enhances chilling tolerance by regulating antioxidant enzymes	Annual	Dicot	Li et al., 2012
	*Arabidopsis thaliana* (L.)		30 μM	Induced freezing tolerance	Annual	Dicot	Hu et al., 2013
	*Eriobotrya japonica* (L.)		10 μM	Alleviates the chilling injury in the fruits of plants	Perennial	Dicot	Cai et al., 2011b

			**Ca^2^**^+^				

	*Solanum lycopersicum* (L.)		27 mM	Improvement carbon fixation, electron transport, etc.	Annual	Dicot	Zhang G. et al., 2014
	*Cynodon dactylon* (L.)		1, 5, 10, and 20 mM	Antioxidant activation and metabolic homeostasis	Perennial	Monocot	Shi et al., 2014

			**GSH**				

	*Eriobotrya japonica* (L.)		50, 100, and 300 mg/L	Increase in membrane fluidity and decrease in lipid peroxidation	Perennial	Dicot	Wu et al., 2010

## Possible Interaction Between Jasmonates, Calcium, and Glutathione

Plants have different capabilities of combating abiotic stress depending upon their antioxidant expression system (Davenport et al., 2003). Plant hormones regulate the adaptive responses that are indispensable for a plant to adapt itself to abiotic stress. JA increases antioxidant responses against abiotic stress in plants. JA effectively reduces oxidative stress by measuring the decrease in thiobarbituric reactive substance levels, increased GSH content, and scavenging of ROS via expression of enzymatic antioxidants (Maksymiec and Krupa, 2002; Chen Y. et al., 2011). Abiotic stress induces Ca^2+^ influx causing cold acclimation related necessary cellular alterations. Calcium signaling is one of the most vital signaling mechanisms that affect the JA-mediated signaling system inside plant cell via calcium channels (Fisahn et al., 2004; Beyhl et al., 2009; Lu et al., 2016). Generation and accumulation of ROS due to abiotic stresses triggers the opening of Ca^2+^ channels (Demidchik et al., 2018). Regulation and biosynthesis of JA is governed by levels of Ca^2+^ fluctuations (Wasternack and Song, 2017). However, in leaf cells of Arabidopsis JA tend to induce the increase in Ca^2+^ levels by mediating Apo- plastic calcium influx (Lu et al., 2016). It causes significant increase in Ca^2+^ into the cell due to immediate influx of Ca^2+^ ions as seen in roots of winter wheat (Erlandson and Jensén, 2006), alfa alfa (Monroy and Dhindsa, 1995), and maize (Rincon and Hanson, 1986). Drastic changes in Ca^2+^ levels of lodicle cells of panicles of rice and guard cells of Arabidopsis by exogenous application of MeJA has been reported, but transient rise in Ca^2+^ levels can also occur by other JAs in the plant cell cytosol and nucleoplasm (Qin et al., 2005; Walter et al., 2007). Ca^2+^ channel blocker like ruthenium red disrupted the Ca^2+^ transients in potato plants that also hinders the JA formation (Fisahn et al., 2004). Basal level of JA expression was increased in Arabidopsis by gain of function of two pore calcium channel 1 (TPC1) (Bonaventure et al., 2007). JA has been found to cause Ca^2+^ influx via AtCNGC2 calcium channel in epidermal cells of Arabidopsis (Lu et al., 2016). Increase in cytosolic Ca^2+^ levels result in the activation of Ca^2+^ dependent protein kinases (CDPK), calmodulins (CAM), etc., resulting in further integration of stress response pathways. This increase in the cytosolic Ca^2+^ occurs due to Ca^2+^ influx from external or release stores. It has been observed that JA also tends to induce Ca^2+^ mobilization that in turn interacts with CAM or CAM like proteins (CML) to modulate the expression of JA responsive genes like JR1 (Sun et al., 2006). Accumulation of CAM 1 type and CAM 3 type proteins was reported in tobacco plants followed by JA treatment (Yamakawa et al., 2001). CML42 of Arabidopsis is deciphered to have a crucial role in calcium mediated JA biosynthesis (Vadassery et al., 2012a). CAM gene, CAM binding protein, and CML expression also increased in response to MeJA (Bergey and Ryan, 1999; Yang and Poovaiah, 2002; Vadassery et al., 2012b). Moreover, CDPK has also seen to be upregulated by JA as they seem to trigger the formation of OPDA (Ludwig et al., 2005). Inhibitors of JA synthesis prevented the inhibitory effect induced by abiotic stress like heavy metals, on the accumulation of chlorophyll and photosynthesis (Maksymiec and Krupa, 2002). Upregulation of MeJA mediated stress defense by changing the protein profile thereby controlling the photosynthesis and antioxidant metabolism (Chen Y. et al., 2011; Maserti et al., 2011). Changes in the intracellular redox environment of a plant due to generation of ROS in response to various abiotic stresses disturbs the plants cellular physiology (Ogawa et al., 2005). ROS is sequestered via production of antioxidants like GSH (Noctor and Foyer, 1998). Moreover, in response to JA and heavy metals increase in GSH in plants occurs via the expression of genes transcribing the enzymes for GSH production (Schafer et al., 1997; Xiang and Oliver, 1998). JA also leads to increased activity of GCL and GS. Increase of GCL activity causes more glutathione disulfide or oxidized glutathione (GSSG) formation. GSSG in turn causes release of Ca^2+^ thereby also affecting Ca^2+^ signatures (Gómez et al., 2004; Hicks et al., 2007; Sun et al., 2009). Moreover, in response to GSH and GSSG treatment, Ca^2+^ release occurs in plants as seen in tobacco leaf. Ca^2+^ related response is linked to overall GSH supplied to the leaf. Therefore, GSSG has an effect on calcium signatures (Gómez et al., 2004). However, JA biosynthesis and signaling is in turn regulated by Ca^2+^ (Wasternack and Hause, 2013). Glutathione status is modulated by various abiotic stresses that affect the abundance of transcripts related with JA signaling, synthesis, and downstream cascade (Gómez et al., 2004; Han et al., 2013). OPR3, one of the JA biosynthetic enzyme expressions, increased with Ca^2+^ treatment (Chotikacharoensuk et al., 2006). It is also found that Ca^2+^ signaling results in increased levels of OPR3 and JA (Gust et al., 2005).

It is inferred that there must be crosstalk between the JA induced influx of apoplast and ionistol triphosphate sensitive Ca^2+^ stores as JA induced influx of extracellular Ca^2+^ concentration can be via nifedipine sensitive Ca^2+^ channel in the plasma membrane and expression of downstream genes to JA (Sun et al., 2006; Ladyzhenskaia and Korableva, 2008). It has also been demonstrated that for JA synthesis influx Ca^2+^ is necessary for elicitor induction (Hu et al., 2009). However, JA-induced signaling cascade may lead to activation of nifedipine sensitive channels to increase in cytosolic Ca^2+^ which in turn causes release of Ca^2+^ from intra-cellular stores (Sun et al., 2009). JAs also might be involved in transducing signaling pathways and upregulation of the GSH metabolic genes and encourages the synthesis of GSH which eliminates peroxides via ascorbate–glutathione cycle (Smeets et al., 2005; Rouhier et al., 2008). MeJA has found to be responsible for increased expression of JA and glutathione biosynthesis enzymes (Jung et al., 2007).

**FIGURE 3:**
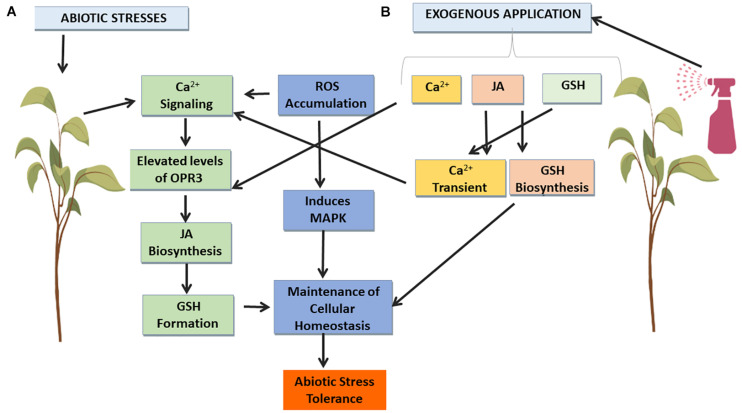
Signaling pathways involved in abiotic stress tolerance. **(A)** Abiotic stresses causes ROS accumulation that leads to activation of Ca^2+^ signaling and MAPK pathways to combat abiotic stresses in plants by maintaining cellular homeostasis. **(B)** Exogenous application of JA, Ca^2+^ and GSH, respectively, to plants prior to or under abiotic stress mediates activation of Ca^2+^ signaling and MAPK pathways via possible crosstalk mechanisms so as to further strengthen the cellular homeostatic mechanisms of plants, thereby imparting abiotic stress tolerance.

The whole cross talk or interaction between JA, Ca^2+^, and GSH is summarized in Figure 3.

## Phyto-Hormone Cross Talk Under Abiotic Stress

Plant hormones have critical roles in mediating the abiotic stress tolerance under unfavorable environmental conditions (Alhaithloul et al., 2020). Abiotic stresses lead to the induction of signal transduction pathways that helps a plant to adapt itself to changing environmental milieu (Dolferus, 2014; Ingole et al., 2021; Kolbert et al., 2021). It leads to the ROS generation and various phyto-hormone accumulation along with remodeling of gene expression in accordance with activation of preferable defense response. These phytohormones mediated signaling and interaction renders them ultimate entity for conferring abiotic stress tolerance in plants (Nguyen et al., 2016; Verma V. et al., 2016; Singh et al., 2019). Phytohormones cross talk in abiotic stress and its link with development of plant stress tolerance in accordance with JA, GSH and Ca^2+^ is discussed below.

### JA Phyto-Hormone Cross Talk Under Abiotic Stress

**FIGURE 4:**
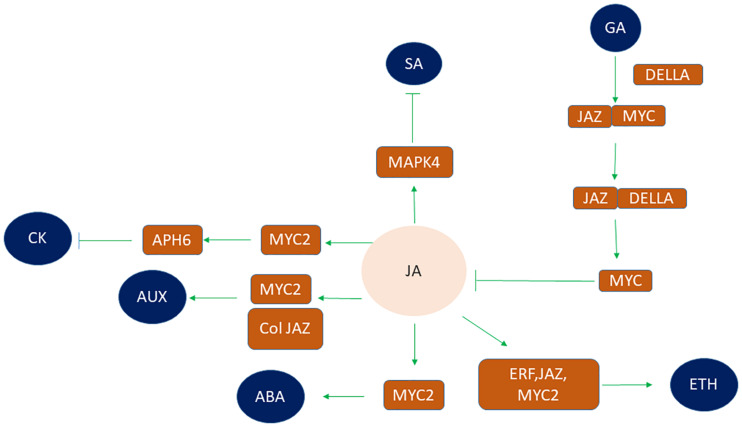
Crosstalk between phytohormones with JA under abiotic stress conditions via different transcription factors. JA interacts with other phytohormones such as SA, ETH, ABA, GA, AUX, and CK in order to regulate plant abiotic stress. JA signaling inhibits SA by modulating mitogen activating protein kinase (MAPK4). Similarly, crosstalk of JA and ETH signaling pathway occurs through interaction of three TF’s viz ERF, JAZ, and MYC2 thereby regulating plant stress response. MYC2 also participates in crosstalk of JA and ABA signaling pathway. DELLA interacts with JAZ in absence of GA to release MYC thereby inhibiting JA biosynthesis and causing activation of MYC downstream genes. However, in the presence of GA, DELLA gets degraded so allowing JAZ-MYC interaction. COL, MYC2, and JAZ are the core components in the crosstalk of JA and AUX signaling pathways. JA mediated inhibition of CK signaling by MYC2 and AHP6 transcription factors. (AUX, auxin; SA, salicylic acid; ETH, ethylene; ABA, abscisic acid; GA, gibberellic acid; CK, cytokinin).

Under multiple environmental stresses, plant hormones allocate limited resources to respond to the most serious stress and develop various signaling pathways to regulate the balance between plant growth and defense response (Tian et al., 2003; Matyssek et al., 2005; Sharma et al., 2013). Understanding the similarities and differences of plant hormone signaling may be important in agricultural production. The crosstalk between plant hormones is of vital importance in plant stress response (He et al., 2017). JA does not work independently but acts in a complex signaling network combined with other plant hormone signaling pathways (Ahmad et al., 2016; He et al., 2017; Hu et al., 2017; Wasternack and Strnad, 2018). Kazan (2015) elucidated the immense role of JA and ethylene in abiotic stress. JA and ET are known to regulate plant tolerance against abiotic stress like drought cold salinity through coordination or antagonistically (van der Fits and Memelink, 2000; Zhai et al., 2013). Ethylene response factors (ERFs) that confer roles in abiotic stress combating mechanism are induced by JA signaling apart from ethylene, thereby facilitating cross talk between them (Ramegowda and Senthil-Kumar, 2015). ERF-domain transcription factor ORA59 of *A. thaliana*, ET INSENSITIVE3 (EIN3) and its homolog EIN3-like 1 (EIL1), as well as JAZs-MYC2 are involved in the crosstalk between JA and Ethylene signaling pathways (Zhu, 2003, 2014; Zhang X. et al., 2014; Zhu and Lee, 2015). JA interacts with ABA under abiotic stress to cause a physiological response to overcome abiotic stress factors (Gomez-Cadenas et al., 2015). MYC2 and JAZ have roles in cross talk between them (Chen Q. et al., 2011). JA cross talk with ABA imparts cold stress tolerance (Hu et al., 2013). MYC2, the core regulator of the JA signaling mechanism, contributes in the ABA signaling cascade in response to drought stress (Abe et al., 2003; Liu et al., 2014). Moreover, JA and SA also have the same regulator glutaredoxin GRX480 which maintains protein redox regulation due to its ability to catalyze disulfide transitions (Meldau et al., 2012). Mitogen-activated protein kinase 4 (MAPK4) is a negative regulator of SA signaling and positive regulator of JA signaling cascade in light stress (Sharma, 2013). The C-terminus of JAZs mediates interaction between JAZs and MYC2 and between JAZs and DELLAs. So, DELLAs can completely interact with JAZs (Hou et al., 2010). In absence of gibberellic acid (GA), DELLA can interact with JAZ and mediates release of MYC2 thereby inhibiting JA biosynthesis and mediating activation MYC2 downstream gene activation (MYB21 and MYB24) (Song et al., 2011). But in the presence of GA, DELLA gets degraded, thus allowing JAZ-MYC2 interaction (Hu et al., 2013). On the contrary, JA delays GA-mediated degradation of DELLA (Yang et al., 2012). JA and auxin signaling coordinately regulate the plant growth and development. COI1, MYC2, and JAZ are the core components in the crosstalk of JA and auxin signaling pathways. In response to exogenous auxin, the activation of auxin–TIR–AUX/IAA–ARF signaling occurs, mediating JA synthesis. The endogenous JA prompts the expression of auxin synthase gene (*ASA1*) and auxin content. JA leads to formation of a complex of COI1 and JAZ leading to the degradation of JAZ, thereby activating the transcriptional activities of MYB21/MYB24 and causing flower development (Chen Q. et al., 2011). JA also interacts with Cytokinin through MYC2 transcription factor. MYC2 is reported to be a negative regulator of cytokinin response by facilitating expression of inhibitor of cytokinin signaling AHP6. JA is involved in decreased expression of PIN-FORMED 7 gene that is involved in the development of xylem and it has been reported that extra xylem formation takes place in roots of Arabidopsis under drought stress (Jang and Do Choi, 2018). So, this opposite interaction between JA and Cytokinin has a role in JA dependent stress response. It is also suggested that differential cytokinin expression under stressful conditions leads to JA – cytokinin interaction at a metabolic level (Le et al., 2012; Jang and Do Choi, 2018). So JA interacts with a different kinds of hormones to regulate the growth and development of plants such as GA, auxin, cytokinin, Ethylene, and SA (Figure 4). These interactions may help to optimize growth and development of plants under abiotic stress conditions.

### Glutathione Phytohormone Cross Talk Under Abiotic Stress

Adverse climatic conditions lead to abiotic stress in plants. Peroxisomal or cytosolic atmosphere leads to electron absorption and subsequently causes oxidative damage via ROS generation (Hasanuzzaman et al., 2017b). ROS-mediated abiotic stress-induces apoptosis or whole plant death in many plant cultivars (Petrov et al., 2015; He et al., 2018). It imparts signals that regulate stress adaptation (Mhamdi and Van Breusegem, 2018). Plants have an antioxidant defense system comprising of non-enzymatic and enzymatic antioxidants in cell organelles, which aids in ROS removal up to a certain level (Gill and Tuteja, 2010). Among this well-defined antioxidant system, Glutathione regulates numerous metabolic functions. Glutathione peroxidase is responsible for ROS detoxification (Hasanuzzaman et al., 2017a). Glutathione increases the plant tolerance to different abiotic stresses, including salinity, drought, high temperature (HT), low temperature, and toxic metal stress (Hasanuzzaman et al., 2013). Exogenously given GSH imparts abiotic stress tolerance in plants (Nahar et al., 2015a, b). Hormonal regulation of GSH and its role in abiotic stress tolerance have been reported in many research findings. Transcriptome analysis revealed that GSH treatment leads to biosynthesis of Auxin, JA, and ABA along with activation signaling cascades pertaining to them (Cheng et al., 2015). GST overexpression in *A. thaliana* plants have signaling and regulatory roles in plant development by maintaining GSH pools (Chen et al., 2012). The increased activities of GST and GPX contributes to improved salt stress in the auxin autotrophic tobacco callus lines (Csiszár et al., 2004). Phytohormones like JA, ABA, Auxin, ethylene, cytokinin, and brassinosteroid induced GST expression in plants (Marrs, 1996; Moons, 2005; Deng et al., 2007). Exogenous Glutathione application brought higher levels of ABA (Chen, 2012). 2, 4-D and NAA (two synthetic auxins) and IAA induced expression of GST8 in Arabidopsis (Bianchi et al., 2002). Moreover, exogenous or endogenous auxin positively regulates the expression intensities of numerous abiotic stress-related genes along with GSH/GSSG pools and GR activity (Shi et al., 2014). JA also regulates GSH concentration and genes for GSH metabolism in Arabidopsis (Akter et al., 2010) and *Agropyron cristatum*, (Sasaki-Sekimoto et al., 2005). It functions as signaling molecule during MeJA signaling in guard cells in Arabidopsis, in addition to intracellular GSH regulating MeJA-induced movements of stomata (Koornneef and Pieterse, 2008). Exogenous SA caused changes in the levels of GSH, GR transcriptomics and activity in maize genotypes and soybean cell suspension thereby mediating abiotic stress tolerance (Knörzer et al., 1999; Kellős et al., 2008). Changes in both SA and GSH expression due to overexpression of the SA gene in rice were correlated with oxidative abiotic stress tolerance (Kusumi et al., 2006). Ethylene has been reported to regulate GSH biosynthesis positively in ozone exposed Arabidopsis leaves (Freeman et al., 2005). The inhibitor of GSH biosynthesis l-buthionine sulfoximine (BSO) effectively reduced the suppression of the JA-responsive gene PDF1.2 by SA, which suggests that SA-mediated control of the cellular redox state is an important trigger for JA signaling (Koornneef and Pieterse, 2008).

### Calcium – Phytohormone Cross Talk Under Abiotic Stress

Plant hormone signaling cascades not only crosstalk with one another, but have also been reported to interact with other signaling molecules such as the Ca^2+^ and mitogen-activated protein kinase (MAPK) pathways during an abiotic stress conditions (Ludwig et al., 2005; Roychoudhury and Paul, 2012; Roychoudhury and Banerjee, 2017). The overlap between hormone-regulated gene expression profiles as adaptive responses of plants to environmental stresses suggests the presence of a complex network with widespread interactions between the different hormone signaling pathways (Suhita et al., 2003, 2004). However, phytohormones like JA and ABA induced the Ca^2+^ transients. The primary role of JA and ABA in the plasma membrane seem to be different to each other. JA aims to encounter the Ca^2+^ channels whereas ABA stimulates effector molecules in the plasma membrane like phospholipase C and D. But at the intracellular Ca^2+^ level, both signaling cascades converge. The intracellular Ca^2+^ level is regulated to a much greater extent by JA rather than by ABA. It has been reported that JA interaction with ABA-regulated stomatal closure by increasing influx of Ca^2+^ causes activation of CDPK-dependent signal pathways, contributing to the drought stress responsiveness (Shi S. et al., 2018). Treatment of Arabidopsis leaves with MeJA or ABA results in less stomatal aperture reduction within 10 min (Munemasa et al., 2007). Though the chemical inhibitors or in ABA-deficient mutants led to inhibition of ABA biosynthesis along with suppression of the MeJA-induced Ca^2+^ oscillations in guard cells (Hossain et al., 2011). Therefore, during stomatal closure MeJA interacts with ABA leading to further Ca^2+^ signaling cascade. Ca^2+^ increase, however, favors the stomata closure by enhancing Slow Anion Channel-Associated 1 (SLAC1) and cytoskeletal rearrangement of plasma membrane (Waidyarathne and Samarasinghe, 2018). Ca^2+^ dependent ABA regulation is related to induction of enzymatic antioxidants (SOD, CAT3, APX, and GR) and non-enzymatic antioxidants [glutathione, ascorbic acid, carotenoids (Ahmad et al., 2010)]. Some drought-responsive CPKs also have some functions like, in rice, OsCPK9 regulates both drought stress tolerance and spikelet fertility through an ABA-dependent manner (Wei et al., 2014). Nevertheless, the precise role of Ca^2+^ in ABA signaling needs to be further explored (Waidyarathne and Samarasinghe, 2018). Gene expression of ethylene-induced ACC oxidase (VR-ACO1) in tissue of root of mung bean was reported due to Cytosolic Ca^2+^ transients. In fact, inhibitors and chelators of Ca^2+^ significantly inhibited the ethylene based gene expression of VR-ACO1, respectively (Jung et al., 2000). Ca^2+^ was seen to augment the conversion of ACC to ethylene in primary roots of *Zea mays* (Hasenstein and Evans, 1986). However, ethylene was found to be responsible for activation of plasma membrane Ca^2+^-permeable channel to increase the Ca^2+^ level in suspension cells of tobacco (*Nicotiana tabacum*) (Zhao et al., 2007). So, this relationship between ethylene and Ca^2+^ seems to be an essential component in abiotic stress response (Acosta-Motos et al., 2017). It has been found that involvement of ethylene in Ca^2+^ induced adventitious rooting under salt stress (Yu et al., 2019). Ca^2+^ plays a pivotal role in the overall downward polar transport of auxin and in the absence of Ca^2+^ basipetal auxin, transport was halted. However, ca^2+^ supplementation can overcome the inhibition of this basipetal transport of auxin (Lee et al., 1983; Allan and Rubery, 1991). So, the transport of auxin plays noteworthy role in the dispersal of Ca^2+^ to developing tissues (Banuelos et al., 1987). The auxin transport pathway seems to be involved in the remodeling of root system architecture in Ca^2+^ mediated alleviation of metal toxicity like Cd toxicity (Li P. et al., 2016).

## Omics Approaches to Study the Roles of JA, Ca^2+^, and GSH Under Abiotic Stress

The intricate molecular controlling systems that have roles in abiotic stress adaptation and tolerance in plants can be interpreted using an ‘omics’ approach (Chawla et al., 2011). The omics technologies have paved the way toward the development of well-established protocols that provide in-depth insights about the gene functionality including their phenotypic effect in defined biological frameworks. Comparative genomic analysis between the plant models like *Thellungiella halophila* and *A. thaliana* have remarkable cold, drought, and salinity tolerance. So, plants tend to modify their omics profile to withstand the fluctuating environment for their existence (Gong et al., 2005). Almost 50% of the plant genes were activated by stresses including drought and salinity. The abiotic stress responding genes can be divided into two classes based either on their response in terms of timescale or on their involvement in tolerance, some seem to respond quickly within seconds or minutes, while others respond slowly (Ramanjulu and Bartels, 2002). It has been reported that about 15% more unknown genes were expressed in the plant subjected to salt stress than in the unstressed plant suggesting that the exposure of plant to abiotic stress results in the surge in expression of genes. In response to various abiotic stresses in plants, proteomic studies has been mostly accepted to explore the protein profiles that might lead to the progression of new strategic ways to improve stress tolerance (Cushman and Bohnert, 2000). Researchers have used various omics approaches to decipher an integrated mode of plant response to different abiotic stresses (Zhu et al., 2017; Bajwa et al., 2018; Parida et al., 2018; Zhang et al., 2018). Treatment of plants with MeJA showed remarkable change in their protein profile. Nearly 194 proteins were differentially expressed in various plant physiological processes. Functional analyses revealed that carbohydrate catabolism was upregulated along with some proteins involved in JA biosynthesis pathway and stress defense (Chen Y. et al., 2011). Multi-omics analysis determined vigorous cascade of transcriptional reprogramming via TF MYC2 and MYC3 that tend to target lots of JA-responsive genes, thereby enabling expression of cohorts of genes that have distinct roles within the JA response. This depicts the complexity of the hormone-response based genome regulatory program. Further, functional importance of MYC2 and MYC3 target genes in JA responses has been validated. Mutations in six genes caused evident disturbances in JA responses, both hypersensitivity and hyposensitivity (Zander et al., 2020). Microarray experiments of wheat and barley cultivars under boron toxicity conditions suggest that genes related to jasmonate biosynthesis and GST can have roles in boron tolerance mechanisms in cereals (Öz et al., 2009). Moreover, plants have a complex antioxidant defense system to scavenge ROS under stress conditions (Hossain et al., 2009). Transgenic plants over express enzymes involved in oxidative stress protection like GPX, SOD, and GR (Tang et al., 2006). The differential expression of Medicago GST (MtGST) were upregulated whereas some of them were downregulated under abiotic stress. Two cluster groups - MtGSTU46 to MtGSTF8 and MtGSTL4 to MtGSTH5 genes were mostly increased in both drought and salinity stresses. Among them, MtGSTU8, MtGSTU17, MtGSTF8, MtGSTT2, and MtGSTZ1 members were mostly upregulated in all cases of these two abiotic stresses (Hasan et al., 2021). AtGSTU19 and AtGSTF2 favors the glutathionylation and binds to JA hormone precursor oxylipin 12-OPDA. So, GST seems to be involved in the export of 12-OPDA from the chloroplast to the site of JA synthesis (peroxisome) (Dixon and Edwards, 2009). Interestingly, greater 12-OPDA level in plants promoted less stomatal aperture and drought tolerance (Savchenko et al., 2014). This points to a potential crosstalk among 12-OPDA (JA) and GSTs as GSTs are quickly induced by 12-OPDA and JA (Mueller et al., 2008). However, GST binding might regulate the temporal signaling of oxylipins under stress. Elevated GST expression correlates with increased stress tolerance as observed in tomato (Sun et al., 2010) and wheat (Gallé et al., 2009). Moreover, OsGSTL2 overexpression imparted a rise in tolerance level against drought, cold stress, and salinity (Kumar et al., 2013). However, the cytosolic Ca^2+^ transients have been reported under various stresses such as heat, cold, salinity, and water (Mahajan and Tuteja, 2005; Tuteja and Mahajan, 2007; Riveras et al., 2015). The promoter region analysis of the Ca^2+^ shows biased nature toward abiotic stress responsive genes. Three among four Ca^2+^ regulated promoter regions were reported to be indispensable for abiotic stress responses (Iqbal et al., 2020). About 30 CDPK genes have been reported in Arabidopsis that have role in abiotic stress responses (Hrabak et al., 2003). Arabidopsis has Ca^2+^-binding calcineurin B-like (CBL) proteins dependent 25 SnRK3-type kinases including SALT OVERLY SENSITIVE 2 (SOS2)/CIPK24/SnRK3 which have important functions in abiotic stress response. SOS3/ScaBP8/CBL10 and SOS2 participates in activation of the plasma membrane Na+/H+ antiporter (SOS1) prerequisite for salinity tolerance (Luan, 2009). Transcriptomics has revealed that CaM binds to regulate various transcription factors called as CAMTAs generating the stress response (Reddy et al., 2011). CAMTAs comprise bZIP, MYB, WRKY, and NAC families of transcription factors (Popescu et al., 2007; Yoon et al., 2008). Several CBLs have a myristoylation site that stimulates membrane association (Ishitani et al., 2000; Kolukisaoglu et al., 2004; Cheong et al., 2007). Moreover, different combinations of CBLs and CIPKs genes have been identified in plants. Genes encoding CBLs or CIPKs are attributed only to the plant kingdom by computer analysis deciphering their function being restricted to plants (Kolukisaoglu et al., 2004). Differential expression of CBL genes indicating their role in abiotic stress response has been reported also (Kudla et al., 2010). So, different omics tools have been employed to understand plants’ responses to abiotic stress conditions. It involves the integration of multiple omics. Systematic use of omics approaches such as metabolomics, transcriptomics, proteomics, and fluxome are means to connect the global data generated via phenomics has led to expansion toward stress biology for revealing the mechanisms the expression of agronomic traits. The comprehensive nature of multi-omic studies provides an entirely new avenue and future research programs that should be well planned to adapt accordingly. Different omics based tools and integrated approaches will provide glimpses of current scenarios and future perspectives to reveal the plant responses and adaptation to a specific abiotic stress.

## Conclusion and Future Perspective

Jasmonic acid plays a key role in plant regulatory and developmental processes. It has a potent role in alleviating abiotic stress conditions in plants. This review of literature is of the opinion that JA biosynthesis and signaling is dependent on Ca^2+^ levels, however, JA itself can modulate the Ca^2+^ transients. Ca^2+^ transients are also seen in varied abiotic stresses as an early response. In addition to this, one of the plant antioxidants GSH also has a pivotal role in abiotic stress response and tolerance. GSH tends to interact with JA and also facilitates the expression of genes involved in JA biosynthesis. Moreover, it has been seen that release of Ca^2+^ from internal plant stores is also mediated by GSH. Exogenous application of JA also results in an increase in cytosolic Ca^2+^ concentration. This all implies possible interactions between JA, Ca^2+^, and GSH which helps in mitigating plant abiotic stress. This kind of study will help to adopt different approaches pertaining to abiotic stress tolerance. So, JA biosynthesis and signaling, calcium transients, and GSH seem to be co-related with each other. This review clearly suggests (1) Ca^2+^ signaling leads to JA formation then followed by GSH. (2) JA also facilitates the expression of GSH and vice versa. (3) JA and GSH both mediate the release of Ca^2+^ from internal plant stores. This type of interaction between the JA, Ca^2+^, and GSH deciphers the novel mechanism of abiotic stress tolerance in plants. Detailed functional characterization of JA, Ca^2+^, and GSH will help us to decipher the core mechanism and identification of various novel entities that could have an important role in this cross talk. It will further help us to understand plant stress biology and unravel the intricate molecular mechanisms that help plants to combat the effect of abiotic stresses which are otherwise a major threat to agricultural productivity. Therefore, it may be concluded that JA, Ca^2+^, and GSH can enhance abiotic stress tolerance via initiating the possibly correlated signaling cascade.

## Author Contributions

SA and NG: conceptualization and compilation of data. SA, NG, and SQ: writing part. SA, NG, MM, MA, NA-S, and AAA: designing of figures and generation of table.

## Conflict of Interest

The authors declare that the research was conducted in the absence of any commercial or financial relationships that could be construed as a potential conflict of interest.

## Abbreviations

JA: jasmonic acid
Ca^2+^: calcium
GSH: glutathione
MeJA: methyl jasmonate
JAZ: jasmonate zip domain
Ile: isoleucine
ROS: reactive oxygen species
Na^+^: sodium
Pb: lead
CAT: catalase
APX: ascorbate peroxidase
LOX3: lipoxygenase3
AsA: ascorbic acid
Cd: cadmium
HM: heavy metal
Cu: copper
RuBisCO: ribulose-1,5-bisphosphate carboxylase oxygenase
SOD: superoxide dismutase
POD: peroxidase
H_2_O_2_: hydrogen peroxide
jar1: methyl jasmonate resistant1
coi1: coronatine insensitive1
oji1: ozone-sensitive and jasmonate insensitive
OPR3: 12-oxophytodienoate reductase 3
GST: glutathione *s-*transferase
CAM: calmodulins
GR: glutathione reductase.

## References

[B1] Abd-Allah E. F. Hashem A. Alqarawi A. A. Wirth S. Egamberdieva D. (2017). Calcium application enhances growth and alleviates the damaging effects induced by Cd stress in sesame (*Sesamum indicum* L.). *J. Plant Interact.* 12 237–243. 10.1080/17429145.2017.1319500

[B2] Abe H. Urao T. Ito T. Seki M. Shinozaki K. Yamaguchi-Shinozaki K. (2003). Arabidopsis AtMYC2 (bHLH) and AtMYB2 (MYB) function as transcriptional activators in abscisic acid signaling. *Plant Cell* 15 63–78. 10.1105/tpc.006130 12509522 PMC143451

[B3] Acosta-Motos J. R. Ortuño M. F. Bernal-Vicente A. Diaz-Vivancos P. Sanchez-Blanco M. J. Hernandez J. A. (2017). Plant responses to salt stress: adaptive mechanisms. *Agronomy* 7:18. 10.3390/agronomy7010018

[B4] Agrawal G. K. Tamogami S. Iwahashi H. Agrawal V. P. Rakwal R. (2003). Transient regulation of jasmonic acid-inducible rice MAP kinase gene (OsBWMK1) by diverse biotic and abiotic stresses. *Plant Physiol. Biochem.* 41 355–361. 10.1016/S0981-9428(03)00030-5

[B5] Ahmad M. A. Murali P. V. (2015). Exogenous jasmonic acid alleviates adverse effects of drought stress in *Allium cepa* L. *Int. J. Geol. Agric. Environ. Sci* 3 10–18.

[B6] Ahmad P. Abass Ahanger M. Nasser Alyemeni M. Wijaya L. Alam P. Ashraf M. (2018). Mitigation of sodium chloride toxicity in *Solanum lycopersicum* L. by supplementation of jasmonic acid and nitric oxide. *J. Plant Interact.* 13 64–72. 10.1080/17429145.2017.1420830

[B7] Ahmad P. Alyemeni M. N. Wijaya L. Alam P. Ahanger M. A. Alamri S. A. (2017). Jasmonic acid alleviates negative impacts of cadmium stress by modifying osmolytes and antioxidants in faba bean (*Vicia faba* L.). *Arch. Agron. Soil Sci.* 63 1889–1899. 10.1080/03650340.2017.1313406

[B8] Ahmad P. Jaleel C. A. Salem M. A. Nabi G. Sharma S. (2010). Roles of enzymatic and nonenzymatic antioxidants in plants during abiotic stress. *Crit. Rev. Biotechnol.* 30 161–175. 10.3109/07388550903524243 20214435

[B9] Ahmad P. Rasool S. Gul A. Sheikh S. A. Akram N. A. Ashraf M. (2016). Jasmonates: multifunctional roles in stress tolerance. *Front. Plant Sci.* 7:813. 10.3389/fpls.2016.00813 27379115 PMC4908892

[B10] Ahmad P. Sarwat M. Bhat N. A. Wani M. R. Kazi A. G. Tran L.-S. P. (2015). Alleviation of cadmium toxicity in *Brassica juncea* L. (Czern. & Coss.) by calcium application involves various physiological and biochemical strategies. *PLoS One* 10:e0114571. 10.1371/journal.pone.0114571 25629695 PMC4309397

[B11] Ahmadi F. I. Karimi K. Struik P. C. (2018). Effect of exogenous application of methyl jasmonate on physiological and biochemical characteristics of *Brassica napus* L. cv. Talaye under salinity stress. *South Afr. J. Bot.* 115 5–11. 10.1016/j.sajb.2017.11.018

[B12] Ainsworth E. A. Yendrek C. R. Sitch S. Collins W. J. Emberson L. D. (2012). The effects of tropospheric ozone on net primary productivity and implications for climate change. *Annu. Rev. Plant Biol.* 63 637–661. 10.1146/annurev-arplant-042110-103829 22404461

[B13] Akram S. Siddiqui M. N. Hussain B. M. N. Al Bari M. A. Mostofa M. G. Hossain M. A. (2017). Exogenous glutathione modulates salinity tolerance of soybean [*Glycine* max (L.) Merrill] at reproductive stage. *J. Plant Growth Regul.* 36 877–888. 10.1007/s00344-017-9691-9

[B14] Akter N. Sobahan M. A. Hossain M. A. Uraji M. Nakamura Y. Mori I. C. (2010). The involvement of intracellular glutathione in methyl jasmonate signaling in *Arabidopsis* guard cells. *Biosci. Biotechnol. Biochem.* 74 2504–2506. 10.1271/bbb.100513 21150111

[B15] Alam M. M. Nahar K. Hasanuzzaman M. Fujita M. (2014). Exogenous jasmonic acid modulates the physiology, antioxidant defense and glyoxalase systems in imparting drought stress tolerance in different *Brassica* species. *Plant Biotechnol. Rep.* 8 279–293. 10.1007/s11816-014-0321-8

[B16] Alhaithloul H. A. S. Abu-Elsaoud A. M. Soliman M. H. (2020). *Abiotic Stress Tolerance in Crop Plants: Role of Phytohormones. In Abiotic Stress in Plants*. London, UK: IntechOpen.

[B17] Ali E. Hussain N. Shamsi I. H. Jabeen Z. Siddiqui M. H. Jiang L. (2018). Role of jasmonic acid in improving tolerance of rapeseed (*Brassica napus* L.) to Cd toxicity. *J. Zhejiang Univ. B* 19 130–146. 10.1631/jzus.B1700191 29405041 PMC5833327

[B18] Ali M. S. Baek K. H. (2020). Jasmonic acid signaling pathway in response to abiotic stresses in plants. *Int. J. Mol. Sci.* 21:621. 10.3390/ijms21020621 31963549 PMC7013817

[B19] Alisofi S. Einali A. Sangtarash M. H. (2020). Jasmonic acid-induced metabolic responses in bitter melon (*Momordica charantia*) seedlings under salt stress. *J. Hortic. Sci. Biotechnol.* 95 247–259. 10.1080/14620316.2019.1663135

[B20] Allagulova C. Avalbaev A. Fedorova K. Shakirova F. (2020). Methyl jasmonate alleviates water stress-induced damages by promoting dehydrins accumulation in wheat plants. *Plant Physiol. Biochem.* 155 676–682. 10.1016/j.plaphy.2020.07.012 32861034

[B21] Allan A. C. Rubery P. H. (1991). Calcium deficiency and auxin transport in *Cucurbita pepo* L. seedlings. *Planta* 183 604–612. 24193855 10.1007/BF00194283

[B22] Ameen N. Amjad M. Murtaza B. Abbas G. Shahid M. Imran M. (2019). Biogeochemical behavior of nickel under different abiotic stresses: toxicity and detoxification mechanisms in plants. *Environ. Sci. Pollut. Res.* 26 10496–10514. 10.1007/s11356-019-04540-4 30835069

[B23] Armstrong W. (1980). “Aeration in higher plants,” in *Advances in Botanical Research*, ed. Woolhouse H. W. (London: Academic Press), 225–332. 10.1016/S0065-2296(08)60089-0

[B24] Asgher M. Khan M. I. R. Anjum N. A. Khan N. A. (2015). Minimising toxicity of cadmium in plants–role of plant growth regulators. *Protoplasma* 252 399–413. 10.1007/s00709-014-0710-4 25303855

[B25] Asgher M. Per T. S. Verma S. Pandith S. A. Masood A. Khan N. A. (2018). Ethylene Supplementation increases PSII efficiency and alleviates chromium-inhibited photosynthesis through increased nitrogen and sulfur assimilation in mustard. *J. Plant Growth Regul.* 37 1300–1317. 10.1007/s00344-018-9858-z

[B26] Asrar Z. Mozafari H. Rezanejad F. Pourseyedi S. Yaghoobi M. M. (2014). Calcium and L-histidine effects on ascorbate-glutathione cycle components under nickel-induced oxidative stress in tomato plants. *Biol. Plant.* 58 709–716. 10.1007/s10535-014-0443-4

[B27] Avalbaev A. Yuldashev R. Fedorova K. Somov K. Vysotskaya L. Allagulova C. (2016). Exogenous methyl jasmonate regulates cytokinin content by modulating cytokinin oxidase activity in wheat seedlings under salinity. *J. Plant Physiol.* 191 101–110. 10.1016/j.jplph.2015.11.013 26748373

[B28] Avanci N. C. Luche D. D. Goldman G. H. Goldman M. H. S. (2010). Jasmonates are phytohormones with multiple functions, including plant defense and reproduction. *Genet. Mol. Res.* 9 484–505. 10.4238/vol9-1gmr754 20391333

[B29] Azeem U. (2018). Ameliorating nickel stress by jasmonic acid treatment in *Zea mays* L. *Russ. Agric. Sci.* 44 209–215. 10.3103/s1068367418030035

[B30] Bailey-Serres J. Voesenek L. A. C. J. (2008). Flooding stress: acclimations and genetic diversity. *Annu. Rev. Plant Biol.* 59 313–339. 10.1146/annurev.arplant.59.032607.092752 18444902

[B31] Bajwa A. A. Farooq M. Nawaz A. (2018). Seed priming with sorghum extracts and benzyl aminopurine improves the tolerance against salt stress in wheat (*Triticum aestivum* L.). *Physiol. Mol. Biol. Plants* 24 239–249. 10.1007/s12298-018-0512-9 29515318 PMC5834994

[B32] Balaguer J. Almendro M. B. Gomez I. Navarro Pedreño J. Mataix J. (1993). Tomato growth and yield affected by nickel presented in the nutrient solution. *Int. Symp. Water Qual. Quant. Greenhouse* 458 269–272. 10.17660/actahortic.1998.458.34

[B33] Bali S. Jamwal V. L. Kaur P. Kohli S. K. Ohri P. Gandhi S. G. (2019a). Role of P-type ATPase metal transporters and plant immunity induced by jasmonic acid against Lead (Pb) toxicity in tomato. *Ecotoxicol. Environ. Saf.* 174 283–294. 10.1016/j.ecoenv.2019.02.084 30844668

[B34] Bali S. Jamwal V. L. Kohli S. K. Kaur P. Tejpal R. Bhalla V. (2019b). Jasmonic acid application triggers detoxification of lead (Pb) toxicity in tomato through the modifications of secondary metabolites and gene expression. *Chemosphere* 235 734–748. 10.1016/j.chemosphere.2019.06.188 31280042

[B35] Bali S. Kaur P. Jamwal V. L. Gandhi S. G. Sharma A. Ohri P. (2020). Seed priming with jasmonic acid counteracts root knot nematode infection in tomato by modulating the activity and expression of antioxidative enzymes. *Biomolecules* 10:98. 10.3390/biom10010098 31936090 PMC7022828

[B36] Bali S. Kaur P. Kohli S. K. Ohri P. Thukral A. K. Bhardwaj R. (2018). Jasmonic acid induced changes in physio-biochemical attributes and ascorbate-glutathione pathway in Lycopersicon esculentum under lead stress at different growth stages. *Sci. Total Environ.* 645 1344–1360. 10.1016/j.scitotenv.2018.07.164 30248858

[B37] Banuelos G. S. Bangerth F. Marschner H. (1987). Relationship between polar basipetal auxin transport and acropetal Ca2+ transport into tomato fruits. *Physiol. Plant.* 71 321–327. 10.1111/j.1399-3054.1987.tb04350.x

[B38] Baryla A. Carrier P. Franck F. Coulomb C. Sahut C. Havaux M. (2001). Leaf chlorosis in oilseed rape plants (*Brassica napus*) grown on cadmium-polluted soil: causes and consequences for photosynthesis and growth. *Planta* 212 696–709. 10.1007/s004250000439 11346943

[B39] Ben Massoud M. Sakouhi L. Chaoui A. (2019). Effect of plant growth regulators, calcium and citric acid on copper toxicity in pea seedlings. *J. Plant Nutr.* 42 1230–1242. 10.1080/01904167.2019.1609506

[B40] Bergey D. R. Ryan C. A. (1999). Wound-and systemin-inducible calmodulin gene expression in tomato leaves. *Plant Mol. Biol.* 40 815–823. 10487216 10.1023/a:1006247624823

[B41] Berridge M. J. Lipp P. Bootman M. D. (2000). Signal transduction. The calcium entry pas de deux. *Science* 287 1604–1605. 10.1126/science.287.5458.1604 10733429

[B42] Beyhl D. Hörtensteiner S. Martinoia E. Farmer E. E. Fromm J. Marten I. (2009). The fou2 mutation in the major vacuolar cation channel TPC1 confers tolerance to inhibitory luminal calcium. *Plant J.* 58 715–723. 10.1111/j.1365-313x.2009.03820.x 19298454

[B43] Bianchi M. W. Roux C. Vartanian N. (2002). Drought regulation of GST8, encoding the Arabidopsis homologue of ParC/Nt107 glutathione transferase/peroxidase. *Physiol. Plant.* 116 96–105. 10.1034/j.1399-3054.2002.1160112.x 12207667

[B44] Bidar G. Verdin A. Garçon G. Pruvot C. Laruelle F. Grandmougin-Ferjani A. (2008). Changes in fatty acid composition and content of two plants (*Lolium perenne* and *Trifolium repens*) grown during 6 and 18 months in a Metal (Pb, Cd, Zn) contaminated field. *Water Air Soil Pollut.* 192 281–291. 10.1007/s11270-008-9655-6

[B45] Boguszewska D. Zagdańska B. (2012). “ROS as signaling molecules and enzymes of plant response to unfavorable environmental conditions,” in *Oxidative Stress*, eds Lushchak V. Semchyshyn H. M. (Rijeka: IntechOpen). 10.5772/33589

[B46] Bonaventure G. Gfeller A. Proebsting W. M. Hörtensteiner S. Chételat A. Martinoia E. (2007). A gain-of-function allele of TPC1 activates oxylipin biogenesis after leaf wounding in *Arabidopsis*. *Plant J.* 49 889–898. 10.1111/j.1365-313x.2006.03002.x 17253984

[B47] Boominathan R. Doran P. M. (2002). Ni-induced oxidative stress in roots of the Ni hyperaccumulator, *Alyssum bertolonii*. *New Phytol.* 156 205–215. 10.1046/j.1469-8137.2002.00506.x 33873276

[B48] Cai Y. Cao F. Cheng W. Zhang G. Wu F. (2011a). Modulation of exogenous glutathione in phytochelatins and photosynthetic performance against cd stress in the two rice genotypes differing in Cd tolerance. *Biol. Trace Elem. Res.* 143 1159–1173. 10.1007/s12011-010-8929-1 21191821

[B49] Cai Y. Cao S. Yang Z. Zheng Y. (2011b). MeJA regulates enzymes involved in ascorbic acid and glutathione metabolism and improves chilling tolerance in loquat fruit. *Postharvest Biol. Technol.* 59 324–326. 10.1016/j.postharvbio.2010.08.020

[B50] Cassia R. Nocioni M. Correa-Aragunde N. Lamattina L. (2018). Climate change and the impact of greenhouse gasses: CO2 and NO, friends and foes of plant oxidative stress. *Front. Plant Sci.* 9:273. 10.3389/fpls.2018.00273 29545820 PMC5837998

[B51] Causier B. Ashworth M. Guo W. Davies B. (2012). The TOPLESS interactome: a framework for gene repression in *Arabidopsis*. *Plant Physiol.* 158 423–438. 10.1104/pp.111.186999 22065421 PMC3252085

[B52] Chary N. S. Kamala C. T. Raj D. S. S. (2008). Assessing risk of heavy metals from consuming food grown on sewage irrigated soils and food chain transfer. *Ecotoxicol. Environ. Saf.* 69 513–524. 10.1016/j.ecoenv.2007.04.013 17555815

[B53] Chawla K. Barah P. Kuiper M. Bones A. M. (2011). “Systems biology: a promising tool to study abiotic stress responses,” in *Omics and Plant Abiotic Stress Tolerance* eds Tuteja N. Gill S. Tuteja R. (Sharjah: Bentham) 163–172. 10.2174/978160805092511101010163

[B54] Chen C. Huang D. Liu J. (2009). Functions and toxicity of nickel in plants: recent advances and future prospects. *Clean Soil Air Water* 37 304–313. 10.1002/clen.200800199

[B55] Chen F. Wang F. Wu F. Mao W. Zhang G. Zhou M. (2010). Modulation of exogenous glutathione in antioxidant defense system against Cd stress in the two barley genotypes differing in Cd tolerance. *Plant Physiol. Biochem.* 48 663–672. 10.1016/j.plaphy.2010.05.001 20605723

[B56] Chen J. Hu B. Han Y. Yang X. (2008). Detoxification of calcium on soybean seeds under copper stress. *Soybean Sci.* 6.

[B57] Chen J.-H. Jiang H.-W. Hsieh E.-J. Chen H.-Y. Chien C.-T. Hsieh H.-L. (2012). Drought and salt stress tolerance of an *Arabidopsis* glutathione S-transferase U17 knockout mutant are attributed to the combined effect of glutathione and abscisic acid. *Plant Physiol.* 158 340–351. 10.1104/pp.111.181875 22095046 PMC3252094

[B58] Chen P. Yu S. Zhan Y. Kang X. (2006). Effects of jasmonate acid on thermotolerance of grape seedlings. *J. Shihezi Univ. Nat. Sci.* 24 87–91.

[B59] Chen Q. Sun J. Zhai Q. Zhou W. Qi L. Xu L. (2011). The basic helix-loop-helix transcription factor MYC2 directly represses PLETHORA expression during jasmonate-mediated modulation of the root stem cell niche in *Arabidopsis*. *Plant Cell* 23 3335–3352. 10.1105/tpc.111.089870 21954460 PMC3203420

[B60] Chen W.-J. Wang X. Yan S. Huang X. Yuan H.-M. (2019). The ICE-like transcription factor HbICE2 is involved in jasmonate-regulated cold tolerance in the rubber tree (*Hevea brasiliensis*). *Plant Cell Rep.* 38 699–714. 10.1007/s00299-019-02398-x 30830263

[B61] Chen W.-P. Li P. (2002). “Attenuation of reactive oxygen production during chilling in ABA-treated maize cultured cells,” in *Plant Cold Hardiness*, eds Li P. H. Palva E. T. (Boston, MA: Springer), 223–233. 10.1007/978-1-4615-0711-6_16

[B62] Chen Y. (2012). Alleviation effects of exogenous glutathione on the copper toxicity during soybean seeds germination. *Soybean Sci.* 31 247–251.

[B63] Chen Y. Pang Q. Dai S. Wang Y. Chen S. Yan X. (2011). Proteomic identification of differentially expressed proteins in *Arabidopsis* in response to methyl jasmonate. *J. Plant Physiol.* 168 995–1008. 10.1016/j.jplph.2011.01.018 21377756

[B64] Cheng M.-C. Ko K. Chang W.-L. Kuo W.-C. Chen G.-H. Lin T.-P. (2015). Increased glutathione contributes to stress tolerance and global translational changes in *Arabidopsis*. *Plant J.* 83 926–939. 10.1111/tpj.12940 26213235

[B65] Cheong Y. H. Pandey G. K. Grant J. J. Batistic O. Li L. Kim B. (2007). Two calcineurin B-like calcium sensors, interacting with protein kinase CIPK23, regulate leaf transpiration and root potassium uptake in *Arabidopsis*. *Plant J.* 52 223–239. 10.1111/j.1365-313x.2007.03236.x 17922773

[B66] Chini A. Gimenez-Ibanez S. Goossens A. Solano R. (2016). Redundancy and specificity in jasmonate signalling. *Curr. Opin. Plant Biol.* 33 147–156. 10.1016/j.pbi.2016.07.005 27490895

[B67] Chinnusamy V. Schumaker K. Zhu J.-K. (2004). Molecular genetic perspectives on cross-talk and specificity in abiotic stress signalling in plants. *J. Exp. Bot.* 55 225–236. 10.1093/jxb/erh005 14673035

[B68] Chotikacharoensuk T. Arteca R. N. Arteca J. M. (2006). Use of differential display for the identification of touch-induced genes from an ethylene-insensitive *Arabidopsis* mutant and partial characterization of these genes. *J. Plant Physiol.* 163 1305–1320. 10.1016/j.jplph.2005.12.005 16533544

[B69] Chronopoulou E. Ataya F. S. Pouliou F. Perperopoulou F. Georgakis N. Nianiou-Obeidat I. (2017). “Structure, evolution and functional roles of plant glutathione transferases,” in *Glutathione in Plant Growth, Development, and Stress Tolerance*, eds Hossain M. Mostofa M. Diaz-Vivancos P. Burritt D. Fujita M. Tran L. S. (eds) (Cham: Springer), 195–213. 10.1007/978-3-319-66682-2_9

[B70] Chung H. S. Koo A. J. K. Gao X. Jayanty S. Thines B. Jones A. D. (2008). Regulation and function of *Arabidopsis* JASMONATE ZIM-domain genes in response to wounding and herbivory. *Plant Physiol.* 146 952–964. 10.1104/pp.107.115691 18223147 PMC2259048

[B71] Clayton H. Knight M. R. Knight H. McAinsh M. R. Hetherington A. M. (1999). Dissection of the ozone-induced calcium signature. *Plant J.* 17 575–579. 10.1046/j.1365-313x.1999.00411.x 10205911

[B72] Coelho D. G. de Andrade H. M. Marinato C. S. Araujo S. C. de Matos L. P. da Silva V. M. (2020). Exogenous jasmonic acid enhances oxidative protection of *Lemna valdiviana* subjected to arsenic. *Acta Physiol. Plant.* 42 1–9. 10.1007/978-3-319-23534-9_1

[B73] Csiszár J. Szabó M. Erdei L. Márton L. Horváth F. Tari I. (2004). Auxin autotrophic tobacco callus tissues resist oxidative stress: the importance of glutathione S-transferase and glutathione peroxidase activities in auxin heterotrophic and autotrophic calli. *J. Plant Physiol.* 161 691–699. 10.1078/0176-1617-01071 15266716

[B74] Cushman J. C. Bohnert H. J. (2000). Genomic approaches to plant stress tolerance. *Curr. Opin. Plant Biol.* 3 117–124. 10.1016/s1369-5266(99)00052-7 10712956

[B75] Daud M. K. Mei L. Azizullah A. Dawood M. Ali I. Mahmood Q. (2016). Leaf-based physiological, metabolic, and ultrastructural changes in cultivated cotton cultivars under cadmium stress mediated by glutathione. *Environ. Sci. Pollut. Res.* 23 15551–15564. 10.1007/s11356-016-6739-5 27126868

[B76] Davenport S. Gallego S. Benavides M. P. Tomaro M. (2003). Behaviour of antioxidant defense system in the adaptive response to salt stress in *Helianthus annuus* L. cells. *Plant Growth Regul.* 40 81–88. 10.1023/A:1023060211546

[B77] de Campos F. V. de Oliveira J. A. da Silva A. A. Ribeiro C. dos Santos Farnese F. (2019). Phytoremediation of arsenite-contaminated environments: is *Pistia stratiotes* L. a useful tool? *Ecol. Indic.* 104 794–801. 10.1016/j.ecolind.2019.04.048

[B78] de Ollas C. Hernando B. Arbona V. Gómez-Cadenas A. (2013). Jasmonic acid transient accumulation is needed for abscisic acid increase in citrus roots under drought stress conditions. *Physiol. Plant.* 147 296–306. 10.1111/j.1399-3054.2012.01659.x 22671923

[B79] Demidchik V. Shabala S. Isayenkov S. Cuin T. A. Pottosin I. (2018). Calcium transport across plant membranes: mechanisms and functions. *New Phytol.* 220 49–69. 10.1111/nph.15266 29916203

[B80] Deng Z. Zhang X. Tang W. Oses-Prieto J. A. Suzuki N. Gendron J. M. (2007). A proteomics study of brassinosteroid response in *Arabidopsis*. *Mol. Cell. Proteomics* 6 2058–2071. 10.1074/mcp.m700123-mcp200 17848588 PMC2966871

[B81] Dhankher O. P. (2005). Arsenic metabolism in plants: an inside story. *New Phytol.* 503–505. 10.1111/j.1469-8137.2005.01598.x 16313633

[B82] Dhingra M. (2015). Physiological responses and tolerance mechanisms of low. 3 637–646.

[B83] Ding H. Lai J. Wu Q. Zhang S. Chen L. Dai Y.-S. (2016). Jasmonate complements the function of *Arabidopsis* lipoxygenase3 in salinity stress response. *Plant Sci.* 244 1–7. 10.1016/j.plantsci.2015.11.009 26810448

[B84] Ding S. Ma C. Shi W. Liu W. Lu Y. Liu Q. (2017). Exogenous glutathione enhances cadmium accumulation and alleviates its toxicity in Populus × canescens. *Tree Physiol.* 37 1697–1712. 10.1093/treephys/tpx132 29121354

[B85] Ding X. Jiang Y. He L. Zhou Q. Yu J. Hui D. (2016). Exogenous glutathione improves high root-zone temperature tolerance by modulating photosynthesis, antioxidant and osmolytes systems in cucumber seedlings. *Sci. Rep.* 6:35424. 10.1038/srep35424 PMC5067582 27752105

[B86] Dixon D. P. Edwards R. (2009). Selective binding of glutathione conjugates of fatty acid derivatives by plant glutathione transferases. *J. Biol. Chem.* 284 21249–21256. 10.1074/jbc.m109.020107 19520850 PMC2755848

[B87] Dixon D. P. Cummins L. Cole D. J. Edwards R. (1998). Glutathione-mediated detoxification systems in plants. *Curr. Opin. Plant Biol.* 1 258–266. 10.1016/s1369-5266(98)80114-3 10066594

[B88] Dodd A. N. Kudla J. Sanders D. (2010). The language of calcium signaling. *Annu. Rev. Plant Biol.* 61 593–620. 10.1146/annurev-arplant-070109-104628 20192754

[B89] Dolferus R. (2014). To grow or not to grow: a stressful decision for plants. *Plant Sci.* 229 247–261. 10.1016/j.plantsci.2014.10.002 25443851

[B90] Erlandson A. Jensén P. (2006). Influence of low temperature on regulation of Rb+ and Ca2+ influx in roots of winter wheat. *Physiol. Plant.* 75 114–120. 10.1111/j.1399-3054.1989.tb02072.x

[B91] Evans N. H. McAinsh M. R. Hetherington A. M. (2001). Calcium oscillations in higher plants. *Curr. Opin. Plant Biol.* 4 415–420. 10.1016/s1369-5266(00)00194-1 11597499

[B92] Fahad S. Hussain S. Bano A. Saud S. Hassan S. Shan D. (2015). Potential role of phytohormones and plant growth-promoting rhizobacteria in abiotic stresses: consequences for changing environment. *Environ. Sci. Pollut. Res.* 22 4907–4921. 10.1007/s11356-014-3754-2 25369916

[B93] Fang Z. Hu Z. Yin X. Song G. Cai Q. (2020). Exogenous glutathione alleviation of Cd toxicity in Italian ryegrass (*Lolium multiflorum*) by modulation of the Cd absorption, subcellular distribution, and chemical form. *Int. J. Environ. Res. Public Health* 17:8143. 10.3390/ijerph17218143 33158133 PMC7663564

[B94] Farooq M. A. Gill R. A. Islam F. Ali B. Liu H. Xu J. (2016). Methyl jasmonate regulates antioxidant defense and suppresses arsenic uptake in *Brassica napus* L. *Front. Plant Sci.* 7:468. 10.3389/fpls.2016.00468 27148299 PMC4826882

[B95] Farooq M. A. Islam F. Yang C. Nawaz A. Gill R. A. Ali B. (2018). Methyl jasmonate alleviates arsenic-induced oxidative damage and modulates the ascorbate–glutathione cycle in oilseed rape roots. *Plant Growth Regul.* 84 135–148. 10.1007/s10725-017-0327-7

[B96] Fatima A. Singh A. A. Mukherjee A. Agrawal M. Agrawal S. B. (2018). Variability in defence mechanism operating in three wheat cultivars having different levels of sensitivity against elevated ozone. *Environ. Exp. Bot.* 155 66–78. 10.1016/j.envexpbot.2018.06.015

[B97] Fedina I. S. Tsonev T. D. (1997). Effect of pretreatment with methyl jasmonate on the response of *Pisum sativum* to salt stress. *J. Plant Physiol.* 151 735–740. 10.1016/s0176-1617(97)80071-5

[B98] Feys B. J. F. Benedetti C. E. Penfold C. N. Turner J. G. (1994). *Arabidopsis* mutants selected for resistance to the phytotoxin coronatine are male sterile, insensitive to methyl jasmonate, and resistant to a bacterial pathogen. *Plant Cell* 6 751–759. 10.1105/tpc.6.5.751 12244256 PMC160473

[B99] Fisahn J. Herde O. Willmitzer L. Peña-Cortés H. (2004). Analysis of the transient increase in cytosolic Ca2+ during the action potential of higher plants with high temporal resolution: requirement of Ca2+ transients for induction of jasmonic acid biosynthesis and PINII gene expression. *Plant Cell Physiol.* 45 456–459. 10.1093/pcp/pch054 15111720

[B100] Foyer C. H. Souriau N. Perret S. Lelandais M. Kunert K.-J. Pruvost C. (1995). Overexpression of glutathione reductase but not glutathione synthetase leads to increases in antioxidant capacity and resistance to photoinhibition in poplar trees. *Plant Physiol.* 109 1047–1057. 10.1104/pp.109.3.1047 8552710 PMC161408

[B101] Freeman J. L. Garcia D. Kim D. Hopf A. Salt D. E. (2005). Constitutively elevated salicylic acid signals glutathione-mediated nickel tolerance in Thlaspi nickel hyperaccumulators. *Plant Physiol.* 137 1082–1091. 10.1104/pp.104.055293 15734913 PMC1065408

[B102] Freeman J. L. Persans M. W. Nieman K. Albrecht C. Peer W. Pickering I. J. (2004). Increased glutathione biosynthesis plays a role in nickel tolerance in Thlaspi nickel hyperaccumulators. *Plant Cell* 16 2176–2191. 10.1105/tpc.104.023036 15269333 PMC519206

[B103] Fu J. Wu H. Ma S. Xiang D. Liu R. Xiong L. (2017). OsJAZ1 attenuates drought resistance by regulating JA and ABA signaling in rice. *Front. Plant Sci.* 8:2108. 10.3389/fpls.2017.02108 29312378 PMC5733117

[B104] Fujita M. Fujita Y. Noutoshi Y. Takahashi F. Narusaka Y. Yamaguchi-Shinozaki K. (2006). Crosstalk between abiotic and biotic stress responses: a current view from the points of convergence in the stress signaling networks. *Curr. Opin. Plant Biol.* 9 436–442. 10.1016/j.pbi.2006.05.014 16759898

[B105] Galant A. Preuss M. L. Cameron J. Jez J. M. (2011). Plant glutathione biosynthesis: diversity in biochemical regulation and reaction products. *Front. Plant Sci.* 2:45. 10.3389/fpls.2011.00045 22645536 PMC3355797

[B106] Gallé Á Csiszár J. Secenji M. Guóth A. Cseuz L. Tari I. (2009). Glutathione transferase activity and expression patterns during grain filling in flag leaves of wheat genotypes differing in drought tolerance: response to water deficit. *J. Plant Physiol.* 166 1878–1891. 10.1016/j.jplph.2009.05.016 19615785

[B107] Gallego S. Lb P. Ra B. Ce A. Iannone F. Rosales E. (2012). Unravelling Cadmium toxicity and tolerance in plants: insight into regulatory mechanisms. *Environ. Exp. Bot.* 83 33–46 10.1016/j.envexpbot.2012.04.006

[B108] Gang A. Vyas A. Vyas H. (2013). Toxic effects of heavy metals on germination and seedling growth of wheat. *J. Environ. Res. Dev.* 8 206–213.

[B109] Geetika S. Ruqia M. Singh G. S. Sharma P. Parvaiz A. (2020). Jasmonic acid and methyl jasmonate modulate growth, photosynthetic activity and expression of photosystem II subunit genes in *Brassica oleracea* L. *Sci. Rep.* 10:9322. 10.1038/s41598-020-65309-1 PMC7283480 32518304

[B110] Gfeller A. Liechti R. Farmer E. E. (2010). *Arabidopsis* jasmonate signaling pathway. *Sci. Signal.* 3:cm4. 10.1126/scisignal.3109cm4 20159850

[B111] Ghafari H. Tadayon M. R. (2019). Effect of seed soaking with exogenous jasmonic acid on seed germination indexes of sugar beet under drought stress. *J. Environ. Stress. Crop Sci.* 12 1263–1273

[B112] Ghaffari H. Tadayon M. R. Nadeem M. Razmjoo J. Cheema M. (2019). Foliage applications of jasmonic acid modulate the antioxidant defense under water deficit growth in sugar beet. *Span. J. Agric. Res.* 17:e0805. 10.5424/sjar/2019174-15380

[B113] Ghosh S. Debsarkar A. Dutta A. (2019). Technology alternatives for decontamination of arsenic-rich groundwater—A critical review. *Environ. Technol. Innov.* 13 277–303. 10.1016/j.eti.2018.12.003

[B114] Gibbs J. Greenway H. (2003). Mechanisms of anoxia tolerance in plants. I. Growth, survival and anaerobic catabolism. *Funct. Plant Biol.* 30:353. 10.1071/PP98095_ER 32689018

[B115] Gill S. S. Tuteja N. (2010). Reactive oxygen species and antioxidant machinery in abiotic stress tolerance in crop plants. *Plant Physiol. Biochem.* 48 909–930. 10.1016/j.plaphy.2010.08.016 20870416

[B116] Gómez L. D. Vanacker H. Buchner P. Noctor G. Foyer C. H. (2004). Intercellular distribution of glutathione synthesis in maize leaves and its response to short-term chilling. *Plant Physiol.* 134 1662–1671. 10.1104/pp.103.033027 15047902 PMC419840

[B117] Gomez-Cadenas A. Vives V. Zandalinas I. S. Manzi M. Sanchez-Perez M. A. Perez-Clemente M. R. (2015). Abscisic acid: a versatile phytohormone in plant signaling and beyond. *Curr. Protein Pept. Sci.* 16 413–434. 10.2174/1389203716666150330130102 25824385

[B118] Gong Q. Li P. Ma S. Indu Rupassara S. Bohnert H. J. (2005). Salinity stress adaptation competence in the extremophile *Thellungiella halophila* in comparison with its relative *Arabidopsis* thaliana. *Plant J.* 44 826–839. 10.1111/j.1365-313x.2005.02587.x 16297073

[B119] Günthardt-Goerg M. McQuattie C. Scheidegger C. Rhiner C. Matyssek R. (1997). Ozone-induced cytochemical and ultrastructural changes in leaf mesophyll cell walls. *Can. J. For. Res. Can. Rech. For.* 27 453–463. 10.1139/cjfr-27-4-453 33356898

[B120] Gupta N. K. Meena S. K. Gupta S. Khandelwal S. K. (2002). Gas exchange, membrane permeability, and ion uptake in two species of Indian jujube differing in salt tolerance. *Photosynthetica* 40 535–539. 10.1023/A:1024343817290

[B121] Gust A. Saitoh H. Felix G. Freymark G. Miersch O. Wasternack C. (2005). Ethylene-mediated cross-talk between calcium-dependent protein kinase and MAPK signaling controls stress responses in plants. *Proc. Natl. Acad. Sci. U.S.A.* 102 10736–10741. 16027369 10.1073/pnas.0502954102 PMC1176231

[B122] Han Y. Mhamdi A. Chaouch S. Noctor G. (2013). Regulation of basal and oxidative stress-triggered jasmonic acid-related gene expression by glutathione. *Plant. Cell Environ.* 36 1135–1146. 10.1111/pce.12048 23210597

[B123] Hanaka A. Maksymiec W. Bednarek W. (2015). The effect of methyl jasmonate on selected physiological parameters of copper-treated *Phaseolus* coccineus plants. *Plant Growth Regul.* 77 167–177. 10.1007/s10725-015-0048-8

[B124] Hasan M. S. Islam S. Hasan M. N. Das Sajib S. Ahmed S. Islam T. (2020). Genome-wide analysis and transcript profiling identify several abiotic and biotic stress-responsive Glutathione S-transferase genes in soybean. *Plant Gene* 23:100239. 10.1016/j.plgene.2020.100239

[B125] Hasan M. S. Singh V. Islam S. Islam M. S. Ahsan R. Kaundal A. (2021). Genome-wide identification and expression profiling of glutathione S-transferase family under multiple abiotic and biotic stresses in *Medicago truncatula* L. *PLoS One* 16:e0247170. 10.1371/journal.pone.0247170 33606812 PMC7894904

[B126] Hasanuzzaman M. Al Mahmud J. Anee T. Nahar K. Islam T. (2017a). “Drought stress tolerance in wheat: omics approaches in understanding and enhancing antioxidant defense,” in *Abiotic Stress-Mediated Sensing and Signaling in Plants: An Omics Perspective*, eds Zargar S. Zargar M. (Singapore: Springer). 10.1007/978-981-10-7479-0_10

[B127] Hasanuzzaman M. Al Mahmud J. Nahar K. Anee T. I. Inafuku M. Oku H. (2017b). “Responses, adaptation, and ROS metabolism in plants exposed to waterlogging stress,” in *Reactive Oxygen Species and Antioxidant Systems in Plants: Role and Regulation Under Abiotic Stress*, eds Khan M. Khan N. (Singapore: Springer), 257–281. 10.1007/978-981-10-5254-5_10

[B128] Hasanuzzaman M. Alam M. Nahar K. Fujita M. (2014). Trehalose-induced drought stress tolerance: a comparative study among different *Brassica* species. *Plant Omics* 7 271–283. 10.13140/2.1.2883.1366

[B129] Hasanuzzaman M. Nahar K. Alam M. M. Roychowdhury R. Fujita M. (2013). Physiological, biochemical, and molecular mechanisms of heat stress tolerance in plants. *Int. J. Mol. Sci.* 14 9643–9684. 10.3390/ijms14059643 23644891 PMC3676804

[B130] Hasanuzzaman M. Nahar K. Rahman A. Al Mahmud J. Alharby H. F. Fujita M. (2018). Exogenous glutathione attenuates lead-induced oxidative stress in wheat by improving antioxidant defense and physiological mechanisms. *J. Plant Interact.* 13 203–212. 10.1080/17429145.2018.1458913

[B131] Hasegawa P. M. (2013). Sodium (Na+) homeostasis and salt tolerance of plants. *Environ. Exp. Bot.* 92 19–31. 10.1016/j.envexpbot.2013.03.001

[B132] Hasenstein K. H. Evans M. L. (1986). Calcium ion dependency of ethylene production in segments of primary roots of *Zea mays*. *Physiol. Plant.* 67 570–575. 10.1111/j.1399-3054.1986.tb05057.x 11538216

[B133] Hatfield J. L. Prueger J. H. (2015). Temperature extremes: effect on plant growth and development. *Weather Clim. Extrem.* 10 4–10. 10.1016/j.wace.2015.08.001

[B134] He M. He C.-Q. Ding N.-Z. (2018). Abiotic stresses: general defenses of land plants and chances for engineering multistress tolerance. *Front. Plant Sci.* 9:1771. 10.3389/fpls.2018.01771 30581446 PMC6292871

[B135] He X. Jiang J. Wang C. Dehesh K. (2017). ORA59 and EIN3 interaction couples jasmonate-ethylene synergistic action to antagonistic salicylic acid regulation of PDF expression. *J. Integr. Plant Biol.* 59 275–287. 10.1111/jipb.12524 28168848 PMC5396539

[B136] Hell R. Bergmann L. (1988). Glutathione synthetase in tobacco suspension cultures: catalytic properties and localization. *Physiol. Plant.* 72 70–76. 10.1111/j.1399-3054.1988.tb06624.x

[B137] Hell R. Bergmann L. (1990). λ-Glutamylcysteine synthetase in higher plants: catalytic properties and subcellular localization. *Planta* 180 603–612. 10.1007/bf02411460 24202107

[B138] Helleday T. Nilsson R. Jenssen D. (2000). Arsenic [III] and heavy metal ions induce intrachromosomal homologous recombination in the hprt gene of V79 Chinese hamster cells. *Environ. Mol. Mutagen.* 35 114–122. 10.1002/(sici)1098-2280(2000)35:2<114::aid-em6>3.0.co;2-q 10712745

[B139] Hernández I. Chacón O. Rodriguez R. Portieles R. López Y. Pujol M. (2009). Black shank resistant tobacco by silencing of glutathione S-transferase. *Biochem. Biophys. Res. Commun.* 387 300–304. 10.1016/j.bbrc.2009.07.003 19577539

[B140] Hicks L. M. Cahoon R. E. Bonner E. R. Rivard R. S. Sheffield J. Jez J. M. (2007). Thiol-based regulation of redox-active glutamate-cysteine ligase from Arabidopsis thaliana. *Plant Cell* 19 2653–2661. 10.1105/tpc.107.052597 17766407 PMC2002632

[B141] Hossain M. A. Munemasa S. Uraji M. Nakamura Y. Mori I. C. Murata Y. (2011). Involvement of endogenous abscisic acid in methyl jasmonate-induced stomatal closure in *Arabidopsis*. *Plant Physiol.* 156 430–438. 10.1104/pp.111.172254 21402795 PMC3091061

[B142] Hossain M. A. Piyatida P. da Silva J. A. T. Fujita M. (2012). Molecular mechanism of heavy metal toxicity and tolerance in plants: central role of glutathione in detoxification of reactive oxygen species and methylglyoxal and in heavy metal chelation. *J. Bot.* 2012:872875. 10.1155/2012/872875

[B143] Hossain Z. López-Climent M. F. Arbona V. Pérez-Clemente R. M. Gómez-Cadenas A. (2009). Modulation of the antioxidant system in citrus under waterlogging and subsequent drainage. *J. Plant Physiol.* 166 1391–1404. 10.1016/j.jplph.2009.02.012 19362387

[B144] Hou X. Lee L. Y. C. Xia K. Yan Y. Yu H. (2010). DELLAs modulate jasmonate signaling via competitive binding to JAZs. *Dev. Cell* 19 884–894. 10.1016/j.devcel.2010.10.024 21145503

[B145] Hrabak E. M. Chan C. W. M. Gribskov M. Harper J. F. Choi J. H. Halford N. (2003). The *Arabidopsis* CDPK-SnRK superfamily of protein kinases. *Plant Physiol.* 132 666–680. 10.1104/pp.102.011999 12805596 PMC167006

[B146] Hu W. Tian S. B. Di Q. Duan S. H. Dai K. (2018). Effects of exogenous calcium on mesophyll cell ultrastructure, gas exchange, and photosystem II in tobacco (*Nicotiana tabacum* Linn.) under drought stress. *Photosynthetica* 56 1204–1211. 10.1007/s11099-018-0822-8

[B147] Hu X. Li W. Chen Q. Yang Y. (2009). Early signal transduction linking the synthesis of jasmonic acid in plant. *Plant Signal. Behav.* 4 696–697. 10.4161/psb.4.8.9181 19820318 PMC2801378

[B148] Hu Y. Jiang L. Wang F. Yu D. (2013). Jasmonate regulates the INDUCER OF CBF expression-C-repeat binding factor/dre binding factor1 Cascade and freezing tolerance in Arabidopsis. *Plant Cell* 25 2907–2924. 10.1105/tpc.113.112631 23933884 PMC3784588

[B149] Hu Y. Jiang Y. Han X. Wang H. Pan J. Yu D. (2017). Jasmonate regulates leaf senescence and tolerance to cold stress: crosstalk with other phytohormones. *J. Exp. Bot.* 68 1361–1369. 10.1093/jxb/erx004 28201612

[B150] Ingole K. D. Dahale S. K. Bhattacharjee S. (2021). Proteomic analysis of SUMO1-SUMOylome changes during defense elicitation in *Arabidopsis*. *J. Proteomics* 232:104054. 10.1016/j.jprot.2020.104054 33238213

[B151] Iqbal Z. Iqbal M. S. Singh S. P. Buaboocha T. (2020). Ca2+/calmodulin complex triggers CAMTA transcriptional machinery under stress in plants: signaling cascade and molecular regulation. *Front. Plant Sci.* 11. 598327. 10.3389/fpls.2020.598327 33343600 PMC7744605

[B152] Iriti M. Faoro F. (2007). Oxidative Stress, the paradigm of ozone toxicity in plants and animals. *Water Air Soil Pollut.* 187 285–301. 10.1007/s11270-007-9517-7

[B153] Ishitani M. Liu J. Halfter U. Kim C.-S. Shi W. Zhu J.-K. (2000). SOS3 function in plant salt tolerance requires N-myristoylation and calcium binding. *Plant Cell* 12 1667–1677. 10.2307/3871181 11006339 PMC149077

[B154] Jackson M. B. (1985). Ethylene and responses of plants to soil waterlogging and submergence. *Annu. Rev. Plant Physiol.* 36 145–174. 10.1146/annurev.pp.36.060185.001045

[B155] Jackson M. B. Colmer T. D. (2005). Response and adaptation by plants to flooding stress. *Ann. Bot.* 96 501–505. 10.1093/aob/mci205 16217870 PMC4247020

[B156] Jang G. Do Choi Y. (2018). Drought stress promotes xylem differentiation by modulating the interaction between cytokinin and jasmonic acid. *Plant Signal. Behav.* 13:e1451707. 10.1080/15592324.2018.1451707 29533132 PMC5927639

[B157] Jang G. Yoon Y. Do Choi Y. (2020). Crosstalk with jasmonic acid integrates multiple responses in plant development. *Int J Mol Sci.* 21:305. 10.3390/ijms21010305 31906415 PMC6981462

[B158] Jez J. M. Cahoon R. E. Chen S. (2004). *Arabidopsis* thaliana glutamate-cysteine ligase: functional properties, kinetic mechanism, and regulation of activity. *J. Biol. Chem.* 279 33463–33470. 10.1074/jbc.m405127200 15180996

[B159] Jung C. Lyou S. H. Yeu S. Kim M. A. Rhee S. Kim M. (2007). Microarray-based screening of jasmonate-responsive genes in *Arabidopsis thaliana*. *Plant Cell Rep.* 26 1053–1063. 10.1007/s00299-007-0311-1 17297615

[B160] Jung H. Kong M.-S. Lee B.-R. Kim T.-H. Chae M.-J. Lee E.-J. (2019). Exogenous glutathione increases arsenic translocation into shoots and alleviates arsenic-induced oxidative stress by sustaining ascorbate–glutathione homeostasis in rice seedlings. *Front. Plant Sci.* 10:1089. 10.3389/fpls.2019.01089 31572411 PMC6754068

[B161] Jung S. (2004). Effect of chlorophyll reduction in *Arabidopsis thaliana* by methyl jasmonate or norflurazon on antioxidant systems. *Plant Physiol. Biochem.* 42 225–231. 10.1016/j.plaphy.2004.01.001 15051046

[B162] Jung T. Lee J. H. Cho M. H. Kim W. T. (2000). Induction of 1-aminocyclopropane-1-carboxylate oxidase mRNA by ethylene in mung bean roots: possible involvement of Ca2+ and phosphoinositides in ethylene signalling. *Plant. Cell Environ.* 23 205–213. 10.1046/j.1365-3040.2000.00534.x

[B163] Kang D.-J. Seo Y.-J. Lee J.-D. Ishii R. Kim K. U. Shin D. H. (2005). Jasmonic acid differentially affects growth, ion uptake and abscisic acid concentration in salt-tolerant and salt-sensitive rice cultivars. *J. Agron. Crop Sci.* 191 273–282. 10.1111/j.1439-037X.2005.00153.x

[B164] Kanna M. Tamaoki M. Kubo A. Nakajima N. Rakwal R. Agrawal G. (2004). Isolation of an ozone-sensitive and jasmonate-semi-insensitive *Arabidopsis* mutant (oji1). *Plant Cell Physiol.* 44 1301–1310. 10.1093/pcp/pcg157 14701925

[B165] Kanu A. S. Ashraf U. Mo Z. Baggie I. Charley C. S. Tang X. (2019). Calcium amendment improved the performance of fragrant rice and reduced metal uptake under cadmium toxicity. *Environ. Sci. Pollut. Res.* 26 24748–24757. 10.1007/s11356-019-05779-7 31240656

[B166] Karpinski S. Escobar C. Karpinska B. Creissen G. Mullineaux P. M. (1997). Photosynthetic electron transport regulates the expression of cytosolic ascorbate peroxidase genes in Arabidopsis during excess light stress. *Plant Cell* 9 627–640. 10.1105/tpc.9.4.627 9144965 PMC156944

[B167] Kaur H. Sharma P. Sirhindi G. Kaur H. Sharma P. Sirhindi G. (2013). Sugar accumulation and its regulation by jasmonic acid in *Brassica napus* L. under salt stress. *J. Stress Physiol. Biochem.* 9 53–64.

[B168] Kazan K. (2015). Diverse roles of jasmonates and ethylene in abiotic stress tolerance. *Trends Plant Sci.* 20 219–229. 10.1016/j.tplants.2015.02.001 25731753

[B169] Kellős T. Timar I. Szilágyi V. Szalai G. Galiba G. Kocsy G. (2008). Stress hormones and abiotic stresses have different effects on antioxidants in maize lines with different sensitivity. *Plant Biol.* 10 563–572. 10.1111/j.1438-8677.2008.00071.x 18761495

[B170] Keramat B. Kalantari K. M. Arvin M. J. (2010). Effects of methyl jasmonate treatment on alleviation of cadmium damages in soybean. *J. Plant Nutr.* 33 1016–1025. 10.1080/01904161003728685

[B171] Khan M. I. R. Fatma M. Per T. S. Anjum N. A. Khan N. A. (2015). Salicylic acid-induced abiotic stress tolerance and underlying mechanisms in plants. *Front. Plant Sci.* 6:462. 10.3389/fpls.2015.00462 26175738 PMC4485163

[B172] Khan M. N. Komatsu S. (2016). Characterization of post-flooding recovery-responsive enzymes in soybean root and hypocotyl. *J. Plant Biol.* 59 478–487. 10.1007/s12374-016-0048-x

[B173] Khan M. Daud M. K. Basharat A. Khan M. J. Azizullah A. Muhammad N. (2016). Alleviation of lead-induced physiological, metabolic, and ultramorphological changes in leaves of upland cotton through glutathione. *Environ. Sci. Pollut. Res.* 23 8431–8440. 10.1007/s11356-015-5959-4 26782322

[B174] Khan N. A. Asgher M. Per T. S. Masood A. Fatma M. Khan M. I. R. (2016). Ethylene potentiates sulfur-mediated reversal of cadmium inhibited photosynthetic responses in mustard. *Front. Plant Sci.* 7:1628. 10.3389/fpls.2016.01628 27853462 PMC5090167

[B175] Kim Y. Arihara J. Nakayama T. Nakayama N. Shimada S. Usui K. (2004). Antioxidative responses and their relation to salt tolerance in *Echinochloa oryzicola* Vasing and *Setaria virdis* (L.) Beauv. *Plant Growth Regul.* 44 87–92. 10.1007/s10725-004-2746-5

[B176] Knörzer O. C. Lederer B. Durner J. Böger P. (1999). Antioxidative defense activation in soybean cells. *Physiol. Plant.* 107 294–302. 10.1034/j.1399-3054.1999.100306.x 11841302

[B177] Kolbert Z. Lindermayr C. Loake G. J. (2021). The role of nitric oxide in plant biology: current insights and future perspectives. *J Exp Bot.* 72 777–780 10.1093/jxb/erab013 33570126

[B178] Kolukisaoglu Ü Weinl S. Blazevic D. Batistic O. Kudla J. (2004). Calcium sensors and their interacting protein kinases: genomics of the *Arabidopsis* and rice CBL-CIPK signaling networks. *Plant Physiol.* 134 43–58. 10.1104/pp.103.033068 14730064 PMC316286

[B179] Koornneef A. Pieterse C. M. J. (2008). Cross talk in defense signaling. *Plant Physiol.* 146 839–844. 10.1104/pp.107.112029 18316638 PMC2259093

[B180] Kudla J. Batistič O. Hashimoto K. (2010). Calcium signals: the lead currency of plant information processing. *Plant Cell* 22 541–563. 10.1105/tpc.109.072686 20354197 PMC2861448

[B181] Kumar A. Dixit S. Ram T. Yadaw R. B. Mishra K. K. Mandal N. P. (2014). Breeding high-yielding drought-tolerant rice: genetic variations and conventional and molecular approaches. *J. Exp. Bot.* 65 6265–6278. 10.1093/jxb/eru363 25205576 PMC4223988

[B182] Kumar S. Asif M. H. Chakrabarty D. Tripathi R. D. Dubey R. S. Trivedi P. K. (2013). Differential expression of rice lambda class GST gene family members during plant growth, development, and in response to stress conditions. *Plant Mol. Biol. Report.* 31 569–580. 10.1007/s11105-012-0524-5

[B183] Kumar S. Dubey R. S. Tripathi R. D. Chakrabarty D. Trivedi P. K. (2015). Omics and biotechnology of arsenic stress and detoxification in plants: current updates and prospective. *Environ. Int.* 74 221–230. 10.1016/j.envint.2014.10.019 25454239

[B184] Küpper H. (2017). Lead toxicity in plants. *Lead Its Eff. Environ. Heal.* 17 491–500. 10.1515/9783110434330-015 28731308

[B185] Kusumi K. Yaeno T. Kojo K. Hirayama M. Hirokawa D. Yara A. (2006). The role of salicylic acid in the glutathione-mediated protection against photooxidative stress in rice. *Physiol. Plant.* 128 651–661. 10.1111/j.1399-3054.2006.00786.x

[B186] Ladyzhenskaia E. P. Korableva N. P. (2008). [Effect of jasmonic acid on Ca+2 transport through the plasmalemma of potato tuber cells]. *Prikl. Biokhim. Mikrobiol.* 44 709–713. 19145980

[B187] Lang D. Yu X. Jia X. Li Z. Zhang X. (2020). Methyl jasmonate improves metabolism and growth of NaCl-stressed *Glycyrrhiza uralensis* seedlings. *Sci. Hortic. (Amsterdam)* 266:109287. 10.1016/j.scienta.2020.109287

[B188] Le D. T. Nishiyama R. Watanabe Y. Vankova R. Tanaka M. Seki M. (2012). Identification and expression analysis of cytokinin metabolic genes in soybean under normal and drought conditions in relation to cytokinin levels. *PLoS One* 7:e42411. 10.1371/journal.pone.0042411 22900018 PMC3416864

[B189] Lee J. S. Mulkey T. J. Evans M. L. (1983). Gravity-induced polar transport of calcium across root tips of maize. *Plant Physiol.* 73 874–876. 10.1104/pp.73.4.874 16663333 PMC1066570

[B190] Lei G. J. Sun L. Sun Y. Zhu X. F. Li G. X. Zheng S. J. (2020). Jasmonic acid alleviates cadmium toxicity in *Arabidopsis* via suppression of cadmium uptake and translocation. *J. Integr. Plant Biol.* 62 218–227. 10.1111/jipb.12801 30912267

[B191] Li D.-M. Guo Y.-K. Li Q. Zhang J. Wang X.-J. Bai J.-G. (2012). The pretreatment of cucumber with methyl jasmonate regulates antioxidant enzyme activities and protects chloroplast and mitochondrial ultrastructure in chilling-stressed leaves. *Sci. Hortic. (Amsterdam)* 143 135–143. 10.1016/j.scienta.2012.06.020

[B192] Li G. Peng X. Xuan H. Wei L. Yang Y. Guo T. (2013). Proteomic analysis of leaves and roots of common wheat (*Triticum aestivum* L.) under copper-stress conditions. *J. Proteome Res.* 12 4846–4861. 10.1021/pr4008283 24074260

[B193] Li P. Zhao C. Zhang Y. Wang X. Wang X. Wang J. (2016). Calcium alleviates cadmium-induced inhibition on root growth by maintaining auxin homeostasis in *Arabidopsis* seedlings. *Protoplasma* 253 185–200. 10.1007/s00709-015-0810-9 25837011

[B194] Li Q. Lei S. Du K. Li L. Pang X. Wang Z. (2016). RNA-seq based transcriptomic analysis uncovers α-linolenic acid and jasmonic acid biosynthesis pathways respond to cold acclimation in *Camellia japonica*. *Sci. Rep.* 6:36463. 10.1038/srep36463 PMC5098223 27819341

[B195] Liu J. Niu Y. Zhang J. Zhou Y. Ma Z. Huang X. (2018). Ca2+ channels and Ca2+ signals involved in abiotic stress responses in plant cells: recent advances. *Plant Cell Tissue Organ Cult.* 132 413–424. 10.1007/s11240-017-1350-0

[B196] Liu N. Ding Y. Fromm M. Avramova Z. (2014). Different gene-specific mechanisms determine the ‘revised-response’memory transcription patterns of a subset of A. thaliana dehydration stress responding genes. *Nucleic Acids Res.* 42 5556–5566. 10.1093/nar/gku220 24744238 PMC4027201

[B197] Lu M. Zhang Y. Tang S. Pan J. Yu Y. Han J. (2016). AtCNGC2 is involved in jasmonic acid-induced calcium mobilization. *J. Exp. Bot.* 67 809–819. 10.1093/jxb/erv500 26608645

[B198] Lu S. C. (2013). Glutathione synthesis. *Biochim. Biophys. Acta (BBA) General Subj.* 1830 3143–3153. 10.1016/j.bbagen.2012.09.008 PMC3549305 22995213

[B199] Luan S. (2009). The CBL–CIPK network in plant calcium signaling. *Trends Plant Sci.* 14 37–42. 10.1016/j.tplants.2008.10.005 19054707

[B200] Ludwig A. A. Saitoh H. Felix G. Freymark G. Miersch O. Wasternack C. (2005). Ethylene-mediated cross-talk between calcium-dependent protein kinase and MAPK signaling controls stress responses in plants. *Proc. Natl. Acad. Sci. U.S.A.* 102 10736–10741. 10.1073/pnas.0502954102 16027369 PMC1176231

[B201] Ma C. Wang Z. Q. Zhang L. T. Sun M. M. Lin T. B. (2014). Photosynthetic responses of wheat (*Triticum aestivum* L.) to combined effects of drought and exogenous methyl jasmonate. *Photosynthetica* 52 377–385. 10.1007/s11099-014-0041-x

[B202] Ma C. Zhang J. Yuan J. Guo J. Xiong Y. Feng Y. (2019). Differential expression of microRNAs are responsive to drought stress and exogenous methyl jasmonate in wheat (*Triticum aestivum*). *Int. J. Agric. Biol.* 22 475–486.

[B203] Ma L. Ye J. Yang Y. Lin H. Yue L. Luo J. (2019). The SOS2-SCaBP8 complex generates and fine-tunes an AtANN4-dependent calcium signature under salt stress. *Dev. Cell* 48 697–709. 10.1016/j.devcel.2019.02.010 30861376

[B204] Mahajan S. Tuteja N. (2005). Cold, salinity and drought stresses: an overview. *Arch. Biochem. Biophys.* 444 139–158. 10.1016/j.abb.2005.10.018 16309626

[B205] Maksymiec W. Krupa Z. (2002). Jasmonic acid and heavy metals in Arabidopsis plants - a similar physiological response to both stressors? *J. Plant Physiol.* 159 509–515. 10.1078/0176-1617-00610

[B206] Maksymiec W. Wianowska D. Dawidowicz A. L. Radkiewicz S. Mardarowicz M. Krupa Z. (2005). The level of jasmonic acid in *Arabidopsis thaliana* and *Phaseolus coccineus* plants under heavy metal stress. *J. Plant Physiol.* 162 1338–1346. 10.1016/j.jplph.2005.01.013 16425452

[B207] Maksymiec W. Wojcik M. Krupa Z. (2007). Variation in oxidative stress and photochemical activity in *Arabidopsis thaliana* leaves subjected to cadmium and excess copper in the presence or absence of jasmonate and ascorbate. *Chemosphere* 66 421–427. 10.1016/j.chemosphere.2006.06.025 16860844

[B208] Malar S. Shivendra Vikram S. Jc Favas P. Perumal V. (2016). Lead heavy metal toxicity induced changes on growth and antioxidative enzymes level in water hyacinths [*Eichhornia crassipes* (Mart.)]. *Bot. Stud.* 55 1–11. 10.1186/s40529-014-0054-6 28597420 PMC5430585

[B209] Manan A. Ayyub C. M. Pervez M. A. Ahmad R. (2016). Methyl jasmonate brings about resistance against salinity stressed tomato plants by altering biochemical and physiological processes. *Pakistan J. Agric. Sci.* 53 35–41. 10.21162/PAKJAS/16.4441 29808431

[B210] Mansour M. (2004). Mansour MMF and Salama KHA 2004. Cellular basis of salinity tolerance in plants. *Environ. Exp. Bot.* 52 113–122. 10.1016/j.envexpbot.2004.01.009

[B211] Marrs K. A. (1996). The functions and regulation of glutathione S-transferases in plants. *Annu. Rev. Plant Biol.* 47 127–158. 10.1146/annurev.arplant.47.1.127 15012285

[B212] Maserti B. E. Del Carratore R. Della Croce C. M. Podda A. Migheli Q. Froelicher Y. (2011). Comparative analysis of proteome changes induced by the two spotted spider mite *Tetranychus urticae* and methyl jasmonate in citrus leaves. *J. Plant Physiol.* 168 392–402. 10.1016/j.jplph.2010.07.026 20926159

[B213] Masindi V. Muedi K. L. (2018). Environmental contamination by heavy metals. *Heavy Met.* 10 115–132.

[B214] Matyssek R. Agerer R. Ernst D. Munch J. Osswald W. Pretzsch H. (2005). The plant’s capacity in regulating resource demand. *Plant Biol.* 7 560–580. 10.1055/s-2005-872981 16388460

[B215] McAinsh M. R. Pittman J. K. (2009). Shaping the calcium signature. *New Phytol.* 181 275–294. 10.1111/j.1469-8137.2008.02682.x 19121028

[B216] Meister A. (1995). “[3] Glutathione biosynthesis and its inhibition,” in *Biothiols Part B: Thiols in Signal Transduction and Gene Regulation*, ed. Packer L. (San Diego, CA: Academic Press), 26–30. 10.1016/0076-6879(95)52005-8

[B217] Meldau S. Ullman-Zeunert L. Govind G. Bartram S. Baldwin I. T. (2012). MAPK-dependent JA and SA signalling in Nicotiana attenuata affects plant growth and fitness during competition with conspecifics. *BMC Plant Biol.* 12:213. 10.1186/1471-2229-12-213 23148462 PMC3519580

[B218] Melotto M. Mecey C. Niu Y. Chung H. S. Katsir L. Yao J. (2008). A critical role of two positively charged amino acids in the Jas motif of Arabidopsis JAZ proteins in mediating coronatine- and jasmonoyl isoleucine-dependent interactions with the COI1 F-box protein. *Plant J.* 55 979–988. 10.1111/j.1365-313X.2008.03566.x 18547396 PMC2653208

[B219] Meng X. Han J. Wang Q. Tian S. (2009). Changes in physiology and quality of peach fruits treated by methyl jasmonate under low temperature stress. *Food Chem.* 114 1028–1035. 10.1016/j.foodchem.2008.09.109

[B220] Meyer A. J. Fricker M. D. (2002). Control of demand-driven biosynthesis of glutathione in green *Arabidopsis* suspension culture cells. *Plant Physiol.* 130 1927–1937. 10.1104/pp.008243 12481075 PMC166703

[B221] Meyer A. J. May M. J. Fricker M. (2001). Quantitative in vivo measurement of glutathione in *Arabidopsis* cells. *Plant J.* 27 67–78. 11489184 10.1046/j.1365-313x.2001.01071.x

[B222] Mhamdi A. Van Breusegem F. (2018). Reactive oxygen species in plant development. *Development* 145:dev164376. 10.1242/dev.164376 30093413

[B223] Mir M. A. John R. Alyemeni M. N. Alam P. Ahmad P. (2018a). Jasmonic acid ameliorates alkaline stress by improving growth performance, ascorbate glutathione cycle and glyoxylase system in maize seedlings. *Sci. Rep.* 8:2831. 10.1038/s41598-018-21097-3 PMC5809373 29434207

[B224] Mir M. A. Sirhindi G. Alyemeni M. N. Alam P. Ahmad P. (2018b). Jasmonic acid improves growth performance of soybean under nickel toxicity by regulating nickel uptake, redox balance, and oxidative stress metabolism. *J. Plant Growth Regul.* 37 1195–1209. 10.1007/s00344-018-9814-y

[B225] Miranshahi B. Sayyari M. (2016). Methyl jasmonate mitigates drought stress injuries and affects essential oil of summer savory. *J. Agric. Sci. Technol.* 18 1635–1645.

[B226] Mishra S. Srivastava S. Tripathi R. D. Govindarajan R. Kuriakose S. V. Prasad M. N. V. (2006). Phytochelatin synthesis and response of antioxidants during cadmium stress in *Bacopa monnieri* L. *Plant Physiol. Biochem. PPB* 44 25–37. 10.1016/j.plaphy.2006.01.007 16545573

[B227] Mittova V. Theodoulou F. L. Kiddle G. Gómez L. Volokita M. Tal M. (2003). Coordinate induction of glutathione biosynthesis and glutathione-metabolizing enzymes is correlated with salt tolerance in tomato. *FEBS Lett.* 554 417–421. 10.1016/S0014-5793(03)01214-6 14623104

[B228] Monroy A. F. Dhindsa R. S. (1995). Low-temperature signal transduction: induction of cold acclimation-specific genes of alfalfa by calcium at 25 degrees C. *Plant Cell* 7 321–331. 10.1105/tpc.7.3.321 7734966 PMC160785

[B229] Moons A. (2005). Regulatory and functional interactions of plant growth regulators and plant glutathione S-transferases (GSTs). *Vitam. Horm.* 72 155–202. 10.1016/s0083-6729(05)72005-7 16492471

[B230] Mostofa M. G. Seraj Z. I. Fujita M. (2015). Interactive effects of nitric oxide and glutathione in mitigating copper toxicity of rice (*Oryza sativa* L.) seedlings. *Plant Signal. Behav.* 10:e991570. 10.4161/15592324.2014.991570 25897471 PMC4623416

[B231] Mousavi S. R. Niknejad Y. Fallah H. Tari D. B. (2020). Methyl jasmonate alleviates arsenic toxicity in rice. *Plant Cell Rep.* 39 1041–1060. 10.1007/s00299-020-02547-7 32388591

[B232] Mrozek E. Funicelli N. A. (1982). Effect of zinc and lead on germination of Spartina alterniflora loisel seeds at various salinities. *Environ. Exp. Bot.* 22 23–32. 10.1016/0098-8472(82)90005-3

[B233] Mueller S. Hilbert B. Dueckershoff K. Roitsch T. Krischke M. Mueller M. J. (2008). General detoxification and stress responses are mediated by oxidized lipids through TGA transcription factors in *Arabidopsis*. *Plant Cell* 20 768–785. 10.1105/tpc.107.054809 18334669 PMC2329937

[B234] Mukta R. H. Khatun M. R. Nazmul Huda A. K. M. (2019). Calcium induces phytochelatin accumulation to cope with chromium toxicity in rice (*Oryza sativa* L.). *J. Plant Interact.* 14 295–302. 10.1080/17429145.2019.1629034

[B235] Munemasa S. Oda K. Watanabe-Sugimoto M. Nakamura Y. Shimoishi Y. Murata Y. (2007). The coronatine-insensitive 1 mutation reveals the hormonal signaling interaction between abscisic acid and methyl jasmonate in *Arabidopsis* guard cells. Specific impairment of ion channel activation and second messenger production. *Plant Physiol.* 143 1398–1407. 10.1104/pp.106.091298 17220365 PMC1820907

[B236] Myśliwa-Kurdziel B. Prasad M. N. V. Strzałtka K. (2004). “Photosynthesis in heavy metal stressed plants,” in *Heavy Metal Stress in Plants*, ed. Prasad M. N. V. (Berlin: Springer), 146–181. 10.1007/978-3-662-07743-6_6

[B237] Naeem M. Naeem M. S. Ahmad R. Ihsan M. Z. Ashraf M. Y. Hussain Y. (2018). Foliar calcium spray confers drought stress tolerance in maize via modulation of plant growth, water relations, proline content and hydrogen peroxide activity. *Arch. Agron. Soil Sci.* 64 116–131. 10.1080/03650340.2017.1327713

[B238] Naeem M. Traub J. R. Loescher W. (2020). Exogenous calcium mitigates heat stress effects in common bean: a coordinated impact of photoprotection of PSII, up-regulating antioxidants, and carbohydrate metabolism. *Acta Physiol. Plant.* 42 1–13.

[B239] Nahar K. Hasanuzzaman M. Alam M. M. Fujita M. (2015a). Glutathione-induced drought stress tolerance in mung bean: coordinated roles of the antioxidant defence and methylglyoxal detoxification systems. *AoB Plants* 7:plv069. 10.1093/aobpla/plv069 26134121 PMC4526754

[B240] Nahar K. Hasanuzzaman M. Alam M. M. Fujita M. (2015b). Roles of exogenous glutathione in antioxidant defense system and methylglyoxal detoxification during salt stress in mung bean. *Biol. Plant.* 59 745–756. 10.1007/s10535-015-0542-x

[B241] Nanjo Y. Maruyama K. Yasue H. Yamaguchi-Shinozaki K. Shinozaki K. Komatsu S. (2011). Transcriptional responses to flooding stress in roots including hypocotyl of soybean seedlings. *Plant Mol. Biol.* 77 129–144. 10.1007/s11103-011-9799-4 21656040

[B242] Nguyen D. Rieu I. Mariani C. van Dam N. M. (2016). How plants handle multiple stresses: hormonal interactions underlying responses to abiotic stress and insect herbivory. *Plant Mol. Biol.* 91 727–740. 10.1007/s11103-016-0481-8 27095445 PMC4932144

[B243] Noctor G. Foyer C. H. (1998). Ascorbate and glutathione: keeping active oxygen under control. *Annu. Rev. Plant Physiol. Plant Mol. Biol.* 49 249–279. 10.1146/annurev.arplant.49.1.249 15012235

[B244] Noctor G. Mhamdi A. Chaouch S. Han Y. I. Neukermans J. Marquez-Garcia B. (2012). Glutathione in plants: an integrated overview. *Plant. Cell Environ.* 35 454–484. 10.1111/j.1365-3040.2011.02400.x 21777251

[B245] Noriega G. Cruz D. Batlle A. Tomaro M. Balestrasse K. (2012). Heme oxygenase is involved in the protection exerted by jasmonic acid against cadmium stress in soybean roots. *J. Plant Growth Regul.* 31 79–89. 10.1007/s00344-011-9221-0

[B246] Ogawa T. Pan L. Kawai-Yamada M. Yu L.-H. Yamamura S. Koyama T. (2005). Functional analysis of *Arabidopsis* ethylene-responsive element binding protein conferring resistance to bax and abiotic stress-induced plant cell death. *Plant Physiol.* 138 1436–1445. 10.1104/pp.105.063586 15980186 PMC1176415

[B247] Oh M. Nanjo Y. Komatsu S. (2014). Gel-free proteomic analysis of soybean root proteins affected by calcium under flooding stress. *Front. Plant Sci.* 5:559. 10.3389/fpls.2014.00559 25368623 PMC4202786

[B248] Ou L.-J. Liu Z.-B. Zhang Y.-P. Zou X.-X. (2017). Effects of exogenous Ca 2+ on photosynthetic characteristics and fruit quality of pepper under waterlogging stress. *Chil. J. Agric. Res.* 77 126–133. 10.4067/s0718-58392017000200126 27315006

[B249] Overmyer K. Tuominen H. Kettunen R. Betz C. Langebartels C. Sandermann H. J. (2000). Ozone-sensitive arabidopsis rcd1 mutant reveals opposite roles for ethylene and jasmonate signaling pathways in regulating superoxide-dependent cell death. *Plant Cell* 12 1849–1862. 10.1105/tpc.12.10.1849 11041881 PMC149124

[B250] Öz M. T. Yilmaz R. Eyidoğan F. De Graaff L. Yücel M. Öktem H. A. (2009). Microarray analysis of late response to boron toxicity in barley (*Hordeum vulgare* L.) leaves. *Turkish J. Agric. For.* 33 191–202.

[B251] Pandey N. Sharma C. P. (2002). Effect of heavy metals Co2+, Ni2+ and Cd2+ on growth and metabolism of cabbage. *Plant Sci.* 163 753–758. 10.1016/s0168-9452(02)00210-8

[B252] Paradiso A. Caretto S. Leone A. Bove A. Nisi R. De Gara L. (2016). ROS Production and scavenging under anoxia and Re-oxygenation in *Arabidopsis* cells: a balance between redox signaling and impairment. *Front. Plant Sci.* 7:1803. 10.3389/fpls.2016.01803 27990148 PMC5130980

[B253] Parida A. K. Panda A. Rangani J. (2018). “Metabolomics-guided elucidation of abiotic stress tolerance mechanisms in plants,” in *Plant Metabolites and Regulation Under Environmental Stress*, eds Ahmad P. Ahanger M. A. Singh V. P. Tripathi D. K. Alam P. Alyemeni M. N. (Amsterdam: Elsevier), 89–131. 10.1016/b978-0-12-812689-9.00005-4

[B254] Parvin K. Ahamed K. U. Islam M. M. Haque M. N. (2015). Response of tomato plant under salt stress: role of exogenous calcium. *J. Plant Sci.* 10 222–233. 10.3923/jps.2015.222.233

[B255] Parvin K. Nahar K. Hasanuzzaman M. Bhuyan M. H. M. Fujita M. (2019). “Calcium-mediated growth regulation and abiotic stress tolerance in plants,” in *Plant Abiotic Stress Tolerance*, eds Hasanuzzaman M. Hakeem K. Nahar K. Alharby H. (Cham: Springer), 291–331. 10.1007/978-3-030-06118-0_13

[B256] Pasqualini S. Batini P. Ederli L. Antonielli M. (1999). Responses of the xanthophyll cycle pool and ascorbate-glutathione cycle to ozone stress in two tobacco cultivars. *Free Radic. Res.* 31(Suppl.) S67–S73. 10.1080/10715769900301341 10694043

[B257] Paz R. C. Rocco R. A. Reinoso H. Menéndez A. B. Pieckenstain F. L. Ruiz O. A. (2012). Comparative study of alkaline, saline, and mixed saline–alkaline stresses with regard to their effects on growth, nutrient accumulation, and root morphology of *Lotus tenuis*. *J. Plant Growth Regul.* 31 448–459. 10.1007/s00344-011-9254-4

[B258] Pedranzani H. Racagni G. Alemano S. Miersch O. Ramírez I. Peña-Cortés H. (2003). Salt tolerant tomato plants show increased levels of jasmonic acid. *Plant Growth Regul.* 41 149–158. 10.1023/A:1027311319940

[B259] Peng X. Y. Chang B. Xu S. R. Wu W. L. Shi L. (2012). Effects of glutathione on alleviation of copper toxicity and its correlation with accumulation of nitrogen, sulfur and phosphorus in wheat seedlings. *J. Agro Environ. Sci.* 31 867–873.

[B260] Per T. S. Khan M. I. R. Anjum N. A. Masood A. Hussain S. J. Khan N. A. (2018). Jasmonates in plants under abiotic stresses: crosstalk with other phytohormones matters. *Environ. Exp. Bot.* 145 104–120. 10.1016/j.envexpbot.2017.11.004

[B261] Petrov V. Hille J. Mueller-Roeber B. Gechev T. S. (2015). ROS-mediated abiotic stress-induced programmed cell death in plants. *Front. Plant Sci.* 6:69. 10.3389/fpls.2015.00069 25741354 PMC4332301

[B262] Piotrowska A. Bajguz A. Godlewska-Żyłkiewicz B. Czerpak R. Kamińska M. (2009). Jasmonic acid as modulator of lead toxicity in aquatic plant *Wolffia arrhiza* (Lemnaceae). *Environ. Exp. Bot.* 66 507–513. 10.1016/j.envexpbot.2009.03.019

[B263] Poonam S. Kaur H. Geetika S. (2013). Effect of jasmonic acid on photosynthetic pigments and stress markers in &lti&gt;Cajanus cajan&lt;/i&gt; (L.) Millsp. Seedlings under Copper Stress. *Am. J. Plant Sci.* 04 817–823. 10.4236/ajps.2013.44100

[B264] Popescu S. C. Popescu G. V. Bachan S. Zhang Z. Seay M. Gerstein M. (2007). Differential binding of calmodulin-related proteins to their targets revealed through high-density *Arabidopsis* protein microarrays. *Proc. Natl. Acad. Sci. U.S.A.* 104 4730–4735. 10.1073/pnas.0611615104 17360592 PMC1838668

[B265] Porto B. N. Alves J. D. Magalhães P. C. Castro E. M. Campos N. A. Souza K. R. D. (2013). Calcium-Dependent tolerant response of cell wall in maize mesocotyl under flooding stress. *J. Agron. Crop Sci.* 199 134–143. 10.1111/j.1439-037X.2012.00535.x

[B266] Qin Y. Yang J. Zhao J. (2005). Calcium changes and the response to methyl jasmonate in rice lodicules during anthesis. *Protoplasma* 225 103–112. 10.1007/s00709-005-0086-6 15868217

[B267] Qiu Z. Guo J. Zhu A. Zhang L. Zhang M. (2014). Exogenous jasmonic acid can enhance tolerance of wheat seedlings to salt stress. *Ecotoxicol. Environ. Saf.* 104 202–208. 10.1016/j.ecoenv.2014.03.014 24726929

[B268] Qureshi A. S. Hussain M. I. Ismail S. Khan Q. M. (2016). Evaluating heavy metal accumulation and potential health risks in vegetables irrigated with treated wastewater. *Chemosphere* 163 54–61. 10.1016/j.chemosphere.2016.07.073 27521639

[B269] Rakwal R. Tamogami S. Kodama O. (1996). Role of jasmonic acid as a signaling molecule in copper chloride-elicited rice phytoalexin production. *Biosci. Biotechnol. Biochem.* 60 1046–1048. 10.1271/bbb.60.1046

[B270] Ramanjulu S. Bartels D. (2002). Drought-and desiccation-induced modulation of gene expression in plants. *Plant. Cell Environ.* 25 141–151. 10.1046/j.0016-8025.2001.00764.x 11841659

[B271] Ramegowda V. Senthil-Kumar M. (2015). The interactive effects of simultaneous biotic and abiotic stresses on plants: mechanistic understanding from drought and pathogen combination. *J. Plant Physiol.* 176 47–54. 10.1016/j.jplph.2014.11.008 25546584

[B272] Randhawa V. K. Zhou F. Jin X. Nalewajko C. Kushner D. J. (2001). Role of oxidative stress and thiol antioxidant enzymes in nickel toxicity and resistance in strains of the green alga Scenedesmus acutus f. alternans. *Can. J. Microbiol.* 47 987–993. 10.1139/cjm-47-11-987 11766059

[B273] Rao K. V. M. Sresty T. V. S. (2000). Antioxidative parameters in the seedlings of pigeonpea (*Cajanus cajan* (L.) Millspaugh) in response to Zn and Ni stresses. *Plant Sci.* 157 113–128. 10.1016/s0168-9452(00)00273-9 10940475

[B274] Raza A. Charagh S. Zahid Z. Mubarik M. S. Javed R. Siddiqui M. H. (2020). Jasmonic acid: a key frontier in conferring abiotic stress tolerance in plants. *Plant Cell Rep.* 1–29. 10.1007/s00299-020-02614-z 33034676

[B275] Reddy A. M. Kumar S. G. Jyothsnakumari G. Thimmanaik S. Sudhakar C. (2005). Lead induced changes in antioxidant metabolism of horsegram (*Macrotyloma uniflorum* (Lam.) Verdc.) and bengalgram (*Cicer arietinum* L.). *Chemosphere* 60 97–104. 10.1016/j.chemosphere.2004.11.092 15910908

[B276] Reddy A. S. N. Ali G. S. Celesnik H. Day I. S. (2011). Coping with stresses: roles of calcium-and calcium/calmodulin-regulated gene expression. *Plant Cell* 23 2010–2032. 10.1105/tpc.111.084988 21642548 PMC3159525

[B277] Rincon M. Hanson J. B. (1986). Controls on calcium ion fluxes in injured or shocked corn root cells: importance of proton pumping and cell membrane potential. *Physiol. Plant.* 67 576–583. 10.1111/j.1399-3054.1986.tb05058.x

[B278] Riveras E. Alvarez J. M. Vidal E. A. Oses C. Vega A. Gutiérrez R. A. (2015). The calcium ion is a second messenger in the nitrate signaling pathway of *Arabidopsis*. *Plant Physiol.* 169 1397–1404. 10.1104/pp.15.00961 26304850 PMC4587466

[B279] Rouhier N. Lemaire S. D. Jacquot J.-P. (2008). The role of glutathione in photosynthetic organisms: emerging functions for glutaredoxins and glutathionylation. *Annu. Rev. Plant Biol.* 59 143–166. 10.1146/annurev.arplant.59.032607.092811 18444899

[B280] Roychoudhury A. Banerjee A. (2017). “Abscisic acid signaling and involvement of mitogen activated protein kinases and calcium-dependent protein kinases during plant abiotic stress,” in *Mechanism of Plant Hormone Signaling Under Stress* ed. Pandey G. K. (Hoboken, NJ: John Wiley & Sons Ltd) 1 197–241. 10.1002/9781118889022.ch9

[B281] Roychoudhury A. Paul A. (2012). Abscisic acid-inducible genes during salinity and drought stress. *Adv. Med. Biol.* 51 1–78.

[B282] Ruan J. Zhou Y. Zhou M. Yan J. Khurshid M. Weng W. (2019). Jasmonic acid signaling pathway in plants. *Int. J. Mol. Sci.* 20:2479. 10.3390/ijms20102479 31137463 PMC6566436

[B283] Sadeghipour O. (2018). Drought tolerance of cowpea enhanced by exogenous application of methyl jasmonate. *Int. J. Mod. Agric.* 7 51–57.

[B284] Sakhonwasee S. Phingkasan W. (2017). Effects of the foliar application of calcium on photosynthesis, reactive oxygen species production, and changes in water relations in tomato seedlings under heat stress. *Hortic. Environ. Biotechnol.* 58 119–126. 10.1007/s13580-017-0194-1

[B285] Sanders D. Brownlee C. Harper J. F. (1999). Communicating with calcium. *Plant Cell* 11 691–706. 10.1105/tpc.11.4.691 10213787 PMC144209

[B286] Sanders D. Pelloux J. Brownlee C. Harper J. F. (2002). Calcium at the crossroads of signaling. *Plant Cell* 14(Suppl.) S401–S417. 10.1105/tpc.002899 12045291 PMC151269

[B287] Sarwar M. Saleem M. F. Ullah N. Rizwan M. Ali S. Shahid M. R. (2018). Exogenously applied growth regulators protect the cotton crop from heat-induced injury by modulating plant defense mechanism. *Sci. Rep.* 8:17086. 10.1038/s41598-018-35420-5 PMC6244283 30459328

[B288] Sarwat M. Ahmad P. Nabi G. Hu X. (2013). Ca2+ signals: the versatile decoders of environmental cues. *Crit. Rev. Biotechnol.* 33 97–109. 10.3109/07388551.2012.672398 22568501

[B289] Sasaki-Sekimoto Y. Taki N. Obayashi T. Aono M. Matsumoto F. Sakurai N. (2005). Coordinated activation of metabolic pathways for antioxidants and defence compounds by jasmonates and their roles in stress tolerance in *Arabidopsis*. *Plant J.* 44 653–668. 10.1111/j.1365-313x.2005.02560.x 16262714

[B290] Savchenko T. Kolla V. A. Wang C.-Q. Nasafi Z. Hicks D. R. Phadungchob B. (2014). Functional convergence of oxylipin and abscisic acid pathways controls stomatal closure in response to drought. *Plant Physiol.* 164 1151–1160. 10.1104/pp.113.234310 24429214 PMC3938610

[B291] Schafer H. J. Greiner S. Rausch T. Haag-Kerwer A. (1997). In seedlings of the heavy metal accumulator *Brassica juncea* Cu2+ differentially affects transcript amounts for gamma-glutamylcysteine synthetase (gamma-ECS) and metallothionein (MT2). *FEBS Lett.* 404 216–220. 10.1016/s0014-5793(97)00132-4 9119067

[B292] Schmöger M. E. V. Oven M. Grill E. (2000). Detoxification of arsenic by phytochelatins in plants. *Plant Physiol.* 122 793–802. 10.1104/pp.122.3.793 10712543 PMC58915

[B293] Seregin I. Ivanov V. (2001). Physiological aspects of cadmium and lead toxic effects on higher plants. *Russ. J. Plant Physiol.* 48 523–544. 10.1023/A:1016719901147

[B294] Sharma M. Laxmi A. (2016). Jasmonates: emerging players in controlling temperature stress tolerance. *Front. Plant Sci.* 6:1129. 10.3389/fpls.2015.01129 26779205 PMC4701901

[B295] Sharma P. (2013). “Salicylic acid: a novel plant growth regulator–role in physiological processes and abiotic stresses under changing environments,” in *Climate Change and Plant Abiotic Stress Tolerance* eds Tuteja N. Gill S. S. (Hoboken, NJ: John Wiley & Sons) 939–990. 10.1002/9783527675265.ch36

[B296] Sharma R. De Vleesschauwer D. Sharma M. K. Ronald P. C. (2013). Recent advances in dissecting stress-regulatory crosstalk in rice. *Mol. Plant* 6 250–260. 10.1093/mp/sss147 23292878

[B297] Shen Z. Zhang F. Zhang F. (1998). Toxicity of copper and zinc in seedlings of Mung bean and inducing accumulation of polyamine. *J. Plant Nutr.* 21 1153–1162. 10.1080/01904169809365474

[B298] Shi H. Ye T. Zhong B. Liu X. Chan Z. (2014). Comparative proteomic and metabolomic analyses reveal mechanisms of improved cold stress tolerance in bermudagrass (*Cynodon dactylon* (L.) Pers.) by exogenous calcium. *J. Integr. Plant Biol.* 56 1064–1079. 10.1111/jipb.12167 24428341

[B299] Shi S. Li S. Asim M. Mao J. Xu D. Ullah Z. (2018). The *Arabidopsis* calcium-dependent protein kinases (CDPKs) and their roles in plant growth regulation and abiotic stress responses. *Int. J. Mol. Sci.* 19:1900. 10.3390/ijms19071900 29958430 PMC6073581

[B300] Shi Y. Ding Y. Yang S. (2018). Molecular regulation of CBF signaling in cold acclimation. *Trends Plant Sci.* 23 623–637. 10.1016/j.tplants.2018.04.002 29735429

[B301] Short E. F. North K. A. Roberts M. R. Hetherington A. M. Shirras A. D. McAinsh M. R. (2012). A stress-specific calcium signature regulating an ozone-responsive gene expression network in *Arabidopsis*. *Plant J.* 71 948–961. 10.1111/j.1365-313X.2012.05043.x 22563867

[B302] Shukla T. Khare R. Kumar S. Lakhwani D. Sharma D. Asif M. H. (2018). Differential transcriptome modulation leads to variation in arsenic stress response in Arabidopsis thaliana accessions. *J. Hazard. Mater.* 351 1–10. 10.1016/j.jhazmat.2018.02.031 29506000

[B303] Siddiqui M. H. Al-Whaibi M. H. Sakran A. M. Basalah M. O. Ali H. M. (2012). Effect of calcium and potassium on antioxidant system of *Vicia faba* L. under cadmium stress. *Int. J. Mol. Sci.* 13 6604–6619. 10.3390/ijms13066604 22837652 PMC3397484

[B304] Siddiqui M. N. Mostofa M. G. Rahman M. M. Tahjib-Ul-Arif M. Das A. K. Mohi-Ud-Din M. (2020). Glutathione improves rice tolerance to submergence: insights into its physiological and biochemical mechanisms. *J. Biotechnol.* 325 109–118. 10.1016/j.jbiotec.2020.11.011 33188807

[B305] Singh A. Kumar A. Yadav S. Singh I. K. (2019). Reactive oxygen species-mediated signaling during abiotic stress. *Plant Gene* 18:100173. 10.1016/j.plgene.2019.100173

[B306] Singh I. Shah K. (2014). Exogenous application of methyl jasmonate lowers the effect of cadmium-induced oxidative injury in rice seedlings. *Phytochemistry* 108 57–66. 10.1016/j.phytochem.2014.09.007 25301663

[B307] Singh R. Singh S. Parihar P. Singh V. P. Prasad S. M. (2015). Arsenic contamination, consequences and remediation techniques: a review. *Ecotoxicol. Environ. Saf.* 112 247–270. 10.1016/j.ecoenv.2014.10.009 25463877

[B308] Sirhindi G. Mir M. A. Abd-Allah E. F. Ahmad P. Gucel S. (2016). Jasmonic acid modulates the physio-biochemical attributes, antioxidant enzyme activity, and gene expression in Glycine max under nickel toxicity. *Front. Plant Sci.* 7:591. 10.3389/fpls.2016.00591 27242811 PMC4864666

[B309] Sirhindi G. Mir M. A. Sharma P. Gill S. S. Kaur H. Mushtaq R. (2015). Modulatory role of jasmonic acid on photosynthetic pigments, antioxidants and stress markers of *Glycine max* L. under nickel stress. *Physiol. Mol. Biol. Plants* 21 559–565. 10.1007/s12298-015-0320-4 26600682 PMC4646870

[B310] Smeets K. Cuypers A. Lambrechts A. Semane B. Hoet P. Van Laere A. (2005). Induction of oxidative stress and antioxidative mechanisms in Phaseolus vulgaris after Cd application. *Plant Physiol. Biochem. PPB* 43 437–444. 10.1016/j.plaphy.2005.03.007 15890519

[B311] Smirnoff N. (1993). The role of active oxygen in the response of plants to water deficit and desiccation. *New Phytol.* 125 27–58. 10.1111/j.1469-8137.1993.tb03863.x 33874604

[B312] Sohag A. A. M. Tahjib-Ul-Arif M. Polash M. A. S. Chowdhury M. B. Afrin S. Burritt D. J. (2020). Exogenous glutathione-mediated drought stress tolerance in Rice (*Oryza sativa* L.) is associated with lower oxidative damage and favorable ionic homeostasis. *Iran. J. Sci. Technol. Trans. A Sci.* 44 955–971. 10.1007/s40995-020-00917-0

[B313] Somerville C. Briscoe J. (2001). Genetic engineering and water. *Science* 292:2217. 10.1126/science.292.5525.2217 11423621

[B314] Song S. Qi T. Huang H. Ren Q. Wu D. Chang C. (2011). The Jasmonate-ZIM domain proteins interact with the R2R3-MYB transcription factors MYB21 and MYB24 to affect Jasmonate-regulated stamen development in *Arabidopsis*. *Plant Cell* 23 1000–1013. 10.1105/tpc.111.083089 21447791 PMC3082250

[B315] Souza V. L. de Almeida A.-A. F. de S. Souza J. Mangabeira P. A. O. de Jesus R. M. (2014). Altered physiology, cell structure, and gene expression of *Theobroma cacao* seedlings subjected to Cu toxicity. *Environ. Sci. Pollut. Res.* 21 1217–1230. 10.1007/s11356-013-1983-4 23888348

[B316] Stael S. Wurzinger B. Mair A. Mehlmer N. Vothknecht U. C. Teige M. (2012). Plant organellar calcium signalling: an emerging field. *J. Exp. Bot.* 63 1525–1542. 10.1093/jxb/err394 22200666 PMC3966264

[B317] Staswick P. E. Su W. Howell S. H. (1992). Methyl jasmonate inhibition of root growth and induction of a leaf protein are decreased in an *Arabidopsis thaliana* mutant. *Proc. Natl. Acad. Sci. U.S.A.* 89 6837–6840. 10.1073/pnas.89.15.6837 11607311 PMC49599

[B318] Stohs S. J. Bagchi D. Hassoun E. Bagchi M. (2000). Oxidative mechanisms in the toxicity of chromium and cadmium ions. *J. Environ. Pathol. Toxicol. Oncol. Off. Organ Int. Soc. Environ. Toxicol. Cancer* 19 201–213. 10983887

[B319] Subbaiah C. C. Sachs M. M. (2003). Molecular and cellular adaptations of maize to flooding stress. *Ann. Bot.* 91 119–127. 10.1093/aob/mcf210 12509333 PMC4244996

[B320] Suhita D. Kolla V. A. Vavasseur A. Raghavendra A. S. (2003). Different signaling pathways involved during the suppression of stomatal opening by methyl jasmonate or abscisic acid. *Plant Sci.* 164 481–488. 10.1016/s0168-9452(02)00432-6

[B321] Suhita D. Raghavendra A. S. Kwak J. M. Vavasseur A. (2004). Cytoplasmic alkalization precedes reactive oxygen species production during methyl jasmonate-and abscisic acid-induced stomatal closure. *Plant Physiol.* 134 1536–1545. 10.1104/pp.103.032250 15064385 PMC419829

[B322] Sun Q. Yu Y. Wan S. Zhao F. Hao Y. (2009). Is there crosstalk between extracellular and intracellular calcium mobilization in jasmonic acid signaling. *Plant Growth Regul.* 57 7–13. 10.1007/s10725-008-9317-0

[B323] Sun Q.-P. Guo Y. Sun Y. Sun D.-Y. Wang X.-J. (2006). Influx of extracellular Ca2+ involved in jasmonic-acid-induced elevation of [Ca2+]cyt and JR1 expression in *Arabidopsis thaliana*. *J. Plant Res.* 119 343–350. 10.1007/s10265-006-0279-x 16708291

[B324] Sun W. Xu X. Zhu H. Liu A. Liu L. Li J. (2010). Comparative transcriptomic profiling of a salt-tolerant wild tomato species and a salt-sensitive tomato cultivar. *Plant Cell Physiol.* 51 997–1006. 10.1093/pcp/pcq056 20410049

[B325] Taheri Z. Vatankhah E. Jafarian V. (2020). Methyl jasmonate improves physiological and biochemical responses of *Anchusa italica* under salinity stress. *South Afr. J. Bot.* 130 375–382. 10.1016/j.sajb.2020.01.026

[B326] Tahjib-Ul-Arif M. Al Mamun Sohag A. Mostofa M. G. Polash M. A. S. Mahamud A. G. M. S. U. Afrin S. (2020). Comparative effects of ascobin and glutathione on copper homeostasis and oxidative stress metabolism in mitigation of copper toxicity in rice. *Plant Biol.* 23 162–169. 10.1111/plb.13222 33236382

[B327] Tahjib-Ul-Arif M. Roy P. R. Al Mamun Sohag A. Afrin S. Rady M. M. Hossain M. A. (2018). Exogenous calcium supplementation improves salinity tolerance in BRRI Dhan28; a salt-susceptible high-yielding *Oryza sativa* cultivar. *J. Crop Sci. Biotechnol.* 21 383–394. 10.1007/s12892-018-0098-0

[B328] Tamaoki M. (2008). The role of phytohormone signaling in ozone-induced cell death in plants. *Plant Signal. Behav.* 3 166–174. 10.4161/psb.3.3.5538 19513211 PMC2634110

[B329] Tan W. Meng Q. Brestic M. Olsovska K. Yang X. (2011). Photosynthesis is improved by exogenous calcium in heat-stressed tobacco plants. *J. Plant Physiol.* 168 2063–2071. 10.1016/j.jplph.2011.06.009 21803445

[B330] Tang L. Kwon S.-Y. Kim S.-H. Kim J.-S. Choi J. S. Cho K. Y. (2006). Enhanced tolerance of transgenic potato plants expressing both superoxide dismutase and ascorbate peroxidase in chloroplasts against oxidative stress and high temperature. *Plant Cell Rep.* 25 1380–1386. 10.1007/s00299-006-0199-1 16841217

[B331] Tanji K. K. (2006). “Salinity in the soil environment,” in *Salinity: Environment - Plants - Molecules.* eds Läuchli A. Lüttge U. (Dordrecht: Springer) 21–51. 10.1007/0-306-48155-3_2

[B332] Tausz M. Bytnerowicz A. Weidner W. Arbaugh M. Padgett P. Grill D. (1999). Changes in free-radical scavengers describe the susceptibility of *Pinus* ponderosa to ozone in southern californian forests. *Water Air Soil Pollut.* 116 249–254. 10.1023/A:1005200220921

[B333] Tayyab N. Naz R. Yasmin H. Nosheen A. Keyani R. Sajjad M. (2020). Combined seed and foliar pre-treatments with exogenous methyl jasmonate and salicylic acid mitigate drought-induced stress in maize. *PLoS One* 15:e0232269. 10.1371/journal.pone.0232269 32357181 PMC7194409

[B334] Thao N. P. Khan M. I. R. Thu N. B. A. Hoang X. L. T. Asgher M. Khan N. A. (2015). Role of ethylene and its cross talk with other signaling molecules in plant responses to heavy metal stress. *Plant Physiol.* 169 73–84. 10.1104/pp.15.00663 26246451 PMC4577409

[B335] Tian D. Traw M. B. Chen J. Q. Kreitman M. Bergelson J. (2003). Fitness costs of R-gene-mediated resistance in *Arabidopsis thaliana*. *Nature* 423 74–77. 10.1038/nature01588 12721627

[B336] Tuteja N. Mahajan S. (2007). Calcium signaling network in plants: an overview. *Plant Signal. Behav.* 2 79–85. 10.4161/psb.2.2.4176 19516972 PMC2633903

[B337] Vadassery J. Reichelt M. Hause B. Gershenzon J. Boland W. Mithöfer A. (2012a). CML42-mediated calcium signaling coordinates responses to *Spodoptera* herbivory and abiotic stresses in *Arabidopsis*. *Plant Physiol.* 159 1159–1175. 10.1104/pp.112.198150 22570470 PMC3387702

[B338] Vadassery J. Scholz S. S. Mithöfer A. (2012b). Multiple calmodulin-like proteins in *Arabidopsis* are induced by insect-derived (*Spodoptera littoralis*) oral secretion. *Plant Signal. Behav.* 7 1277–1280. 10.4161/psb.21664 22902684 PMC3493413

[B339] van der Fits L. Memelink J. (2000). ORCA3, a jasmonate-responsive transcriptional regulator of plant primary and secondary metabolism. *Science* 289 295–297. 10.1126/science.289.5477.295 10894776

[B340] Vanholme B. Grunewald W. Bateman A. Kohchi T. Gheysen G. (2007). The tify family previously known as ZIM. *Trends Plant Sci.* 12 239–244. 10.1016/j.tplants.2007.04.004 17499004

[B341] Velitchkova M. Fedina I. (1998). Response of photosynthesis of *Pisum sativum* to salt stress as affected by methyl jasmonate. *Photosynthetica* 35 89–97. 10.1023/A:1006878016556

[B342] Verma G. Srivastava D. Narayan S. Shirke P. A. Chakrabarty D. (2020). Exogenous application of methyl jasmonate alleviates arsenic toxicity by modulating its uptake and translocation in rice (*Oryza sativa* L.). *Ecotoxicol. Environ. Saf.* 201:110735. 10.1016/j.ecoenv.2020.110735 32480163

[B343] Verma S. Dubey R. S. (2003). Lead toxicity induces lipid peroxidation and alters the activities of antioxidant enzymes in growing rice plants. *Plant Sci.* 164 645–655. 10.1016/S0168-9452(03)00022-0

[B344] Verma S. Verma P. K. Meher A. K. Dwivedi S. Bansiwal A. K. Pande V. (2016). A novel arsenic methyltransferase gene of Westerdykella aurantiaca isolated from arsenic contaminated soil: phylogenetic, physiological, and biochemical studies and its role in arsenic bioremediation. *Metallomics* 8 344–353. 10.1039/c5mt00277j 26776948

[B345] Verma V. Ravindran P. Kumar P. P. (2016). Plant hormone-mediated regulation of stress responses. *BMC Plant Biol.* 16:86. 10.1186/s12870-016-0771-y 27079791 PMC4831116

[B346] Vivancos P. D. Dong Y. Ziegler K. Markovic J. Pallardo F. V. Pellny T. K. (2010). Recruitment of glutathione into the nucleus during cell proliferation adjusts whole-cell redox homeostasis in *Arabidopsis thaliana* and lowers the oxidative defence shield. *Plant J.* 64 825–838. 10.1111/j.1365-313X.2010.04371.x 21105929

[B347] Vollenweider P. Ottiger M. Gunthardt-Goerg M. S. (2003). Validation of leaf ozone symptoms in natural vegetation using microscopical methods. *Environ. Pollut.* 124 101–118. 10.1016/s0269-7491(02)00412-8 12683987

[B348] Wachter A. Wolf S. Steininger H. Bogs J. Rausch T. (2005). Differential targeting of GSH1 and GSH2 is achieved by multiple transcription initiation: implications for the compartmentation of glutathione biosynthesis in the Brassicaceae. *Plant J.* 41 15–30. 10.1111/j.1365-313x.2004.02269.x 15610346

[B349] Waidyarathne P. Samarasinghe S. (2018). Boolean calcium signalling model predicts calcium role in acceleration and stability of abscisic acid-mediated stomatal closure. *Sci. Rep.* 8:17635. 10.1038/s41598-018-35872-9 PMC6281740 30518777

[B350] Walia H. Wilson C. Condamine P. Liu X. Ismail A. M. Close T. J. (2007). Large-scale expression profiling and physiological characterization of jasmonic acid-mediated adaptation of barley to salinity stress. *Plant. Cell Environ.* 30 410–421. 10.1111/j.1365-3040.2006.01628.x 17324228

[B351] Walter A. Mazars C. Maitrejean M. Hopke J. Ranjeva R. Boland W. (2007). Structural requirements of jasmonates and synthetic analogues as inducers of Ca2+ signals in the nucleus and the cytosol of plant cells. *Angew. Chem. Int. Ed.* 46 4783–4785. 10.1002/anie.200604989 17487903

[B352] Wang J. Song L. Gong X. Xu J. Li M. (2020). Functions of jasmonic acid in plant regulation and response to abiotic stress. *Int. J. Mol. Sci.* 21:1446. 10.3390/ijms21041446 32093336 PMC7073113

[B353] Wang R. Liu S. Zhou F. Ding C. (2014). Exogenous ascorbic acid and glutathione alleviate oxidative stress induced by salt stress in the chloroplasts of *Oryza sativa* L. *Z. Naturforsch. C J. Biosci.* 69 226–236. 10.5560/znc.2013-0117 25069161

[B354] Wani S. H. Kumar V. Shriram V. Sah S. K. (2016). Phytohormones and their metabolic engineering for abiotic stress tolerance in crop plants. *Crop J.* 4 162–176. 10.1016/j.cj.2016.01.010

[B355] Wasternack C. (2007). Jasmonates: an update on biosynthesis, signal transduction and action in plant stress response, growth and development. *Ann. Bot.* 100 681–697. 10.1093/aob/mcm079 17513307 PMC2749622

[B356] Wasternack C. (2015). How jasmonates earned their laurels: past and present. *J. Plant Growth Regul.* 34 761–794. 10.1007/s00344-015-9526-5

[B357] Wasternack C. Hause B. (2002). Jasmonates and octadecanoids: signals in plant stress responses and development. *Prog. Nucleic Acid Res. Mol. Biol.* 72 165–221. 10.1016/s0079-6603(02)72070-9 12206452

[B358] Wasternack C. Hause B. (2013). Jasmonates: biosynthesis, perception, signal transduction and action in plant stress response, growth and development. An update to the 2007 review in Annals of Botany. *Ann. Bot.* 111 1021–1058. 10.1093/aob/mct067 23558912 PMC3662512

[B359] Wasternack C. Song S. (2017). Jasmonates: biosynthesis, metabolism, and signaling by proteins activating and repressing transcription. *J. Exp. Bot.* 68 1303–1321. 27940470 10.1093/jxb/erw443

[B360] Wasternack C. Strnad M. (2018). Jasmonates: news on occurrence, biosynthesis, metabolism and action of an ancient group of signaling compounds. *Int. J. Mol. Sci.* 19:2539. 10.3390/ijms19092539 30150593 PMC6164985

[B361] Wei S. Hu W. Deng X. Zhang Y. Liu X. Zhao X. (2014). A rice calcium-dependent protein kinase OsCPK9 positively regulates drought stress tolerance and spikelet fertility. *BMC Plant Biol.* 14:133. 10.1186/1471-2229-14-133 24884869 PMC4036088

[B362] Wellburn F. A. M. Creissen G. P. Lake J. A. Mullineaux P. M. Wellburn A. R. (1998). Tolerance to atmospheric ozone in transgenic tobacco over-expressing glutathione synthetase in plastids. *Physiol. Plant.* 104 623–629. 10.1034/j.1399-3054.1998.1040415.x 11841302

[B363] White P. J. Broadley M. R. (2003). Calcium in plants. *Ann. Bot.* 92 487–511. 10.1093/aob/mcg164 12933363 PMC4243668

[B364] Wildi B. Lütz C. (1996). Antioxidant composition of selected high alpine plant species from different altitudes. *Plant. Cell Environ.* 19 138–146. 10.1111/j.1365-3040.1996.tb00235.x

[B365] Wu H. Wu X. Li Z. Duan L. Zhang M. (2012). Physiological evaluation of drought stress tolerance and recovery in cauliflower (*Brassica oleracea* L.) seedlings treated with methyl jasmonate and coronatine. *J. Plant Growth Regul.* 31 113–123. 10.1007/s00344-011-9224-x

[B366] Wu J. C. Sun S. H. Ke Y. T. Xie C. P. Chen F. X. (2010). “Effects of glutathione on chloroplast membrane fluidity and the glutathione circulation system in young loquat fruits under low temperature stress,” in *Proceedings of the 3rd International Symposium on Loquat*, Antakya-Hatay, Vol. 887 221–225. 10.17660/actahortic.2011.887.36

[B367] Xiang C. Oliver D. J. (1998). Glutathione metabolic genes coordinately respond to heavy metals and jasmonic acid in *Arabidopsis*. *Plant Cell* 10 1539–1550. 10.1105/tpc.10.9.1539 9724699 PMC144077

[B368] Xu C. Li X. Zhang L. (2013). The effect of calcium chloride on growth, photosynthesis, and antioxidant responses of *Zoysia japonica* under drought conditions. *PLoS One* 8:e68214. 10.1371/journal.pone.0068214 23844172 PMC3699550

[B369] Yadav S. K. (2010). Cold stress tolerance mechanisms in plants. A Review. *Agron. Sustain. Dev.* 30 515–527. 10.1051/agro/2009050

[B370] Yamakawa H. Mitsuhara I. Ito N. Seo S. Kamada H. Ohashi Y. (2001). Transcriptionally and post-transcriptionally regulated response of 13 calmodulin genes to tobacco mosaic virus-induced cell death and wounding in tobacco plant. *Eur. J. Biochem.* 268 3916–3929. 10.1046/j.1432-1327.2001.02301.x 11453984

[B371] Yan Z. Chen J. Li X. (2013). Methyl jasmonate as modulator of Cd toxicity in *Capsicum* frutescens var. fasciculatum seedlings. *Ecotoxicol. Environ. Saf.* 98 203–209. 10.1016/j.ecoenv.2013.08.019 24064260

[B372] Yang B. Z. Liu Z. B. Zhou S. D. Ou L. J. Dai X. Z. Ma Y. Q. (2016). Exogenous Ca 2+ alleviates waterlogging-caused damages to pepper. *Photosynthetica* 54 620–629. 10.1007/s11099-016-0200-3

[B373] Yang D.-L. Yao J. Mei C.-S. Tong X.-H. Zeng L.-J. Li Q. (2012). Plant hormone jasmonate prioritizes defense over growth by interfering with gibberellin signaling cascade. *Proc. Natl. Acad. Sci. U.S.A.* 109 E1192–E1200. 22529386 10.1073/pnas.1201616109 PMC3358897

[B374] Yang T. Poovaiah B. W. (2002). A calmodulin-binding/CGCG box DNA-binding protein family involved in multiple signaling pathways in plants. *J. Biol. Chem.* 277 45049–45058. 10.1074/jbc.m207941200 12218065

[B375] Ye H. Du H. Tang N. Li X. Xiong L. (2009). Identification and expression profiling analysis of TIFY family genes involved in stress and phytohormone responses in rice. *Plant Mol. Biol.* 71 291–305. 10.1007/s11103-009-9524-8 19618278

[B376] Yin Y. Yang R. Han Y. Gu Z. (2015). Comparative proteomic and physiological analyses reveal the protective effect of exogenous calcium on the germinating soybean response to salt stress. *J. Proteomics* 113 110–126. 10.1016/j.jprot.2014.09.023 25284050

[B377] Yoon H.-K. Kim S.-G. Kim S.-Y. Park C.-M. (2008). Regulation of leaf senescence by NTL9-mediated osmotic stress signaling in *Arabidopsis*. *Mol. Cells* 25 438–445. 18443413

[B378] Yoon J. Y. Hamayun M. Lee S.-K. Lee I.-J. (2009). Methyl jasmonate alleviated salinity stress in soybean. *J. Crop Sci. Biotechnol.* 12 63–68. 10.1007/s12892-009-0060-5

[B379] Yu J. Niu L. Yu J. Liao W. Xie J. Lv J. (2019). The involvement of ethylene in calcium-induced adventitious root formation in cucumber under salt stress. *Int. J. Mol. Sci.* 20:1047. 10.3390/ijms20051047 30823363 PMC6429442

[B380] Yu P. Jiang N. Fu W. Zheng G. Li G. Feng B. (2020). ATP hydrolysis determines cold tolerance by regulating available energy for glutathione synthesis in rice seedling plants. *Rice* 13 1–16. 32274603 10.1186/s12284-020-00383-7 PMC7145886

[B381] Yuan H. Zhang Y. Huang S. Yang Y. Gu C. (2015). Effects of exogenous glutathione and cysteine on growth, lead accumulation, and tolerance of Iris lactea var. chinensis. *Environ. Sci. Pollut. Res.* 22 2808–2816. 10.1007/s11356-014-3535-y 25212813

[B382] Zaid A. Mohammad F. (2018). Methyl jasmonate and nitrogen interact to alleviate cadmium stress in mentha arvensis by regulating physio-biochemical damages and ROS detoxification. *J. Plant Growth Regul.* 37 1331–1348. 10.1007/s00344-018-9854-3

[B383] Zander M. Lewsey M. G. Clark N. M. Yin L. Bartlett A. Guzmán J. P. S. (2020). Integrated multi-omics framework of the plant response to jasmonic acid. *Nat. Plants* 6 290–302. 10.1038/s41477-020-0605-7 32170290 PMC7094030

[B384] Zhai Y. Wang Y. Li Y. Lei T. Yan F. Su L. (2013). Isolation and molecular characterization of GmERF7, a soybean ethylene-response factor that increases salt stress tolerance in tobacco. *Gene* 513 174–183. 10.1016/j.gene.2012.10.018 23111158

[B385] Zhang G. Liu Y. Ni Y. Meng Z. Lu T. Li T. (2014). Exogenous calcium alleviates low night temperature stress on the photosynthetic apparatus of tomato leaves. *PLoS One* 9:e97322. 10.1371/journal.pone.0097322 24828275 PMC4020824

[B386] Zhang L. Xu B. Wu T. Wen M. Fan L. Feng Z. (2017). Transcriptomic analysis of Pak Choi under acute ozone exposure revealed regulatory mechanism against ozone stress. *BMC Plant Biol.* 17:236. 10.1186/s12870-017-1202-4 29216819 PMC5721698

[B387] Zhang Q. Liu Y. Yu Q. Ma Y. Gu W. Yang D. (2020). Physiological changes associated with enhanced cold resistance during maize (*Zea mays*) germination and seedling growth in response to exogenous calcium. *Crop Pasture Sci.* 71 529–538. 10.1071/cp19510

[B388] Zhang X. Yao C. Fu S. Xuan H. Wen S. Liu C. (2018). Stress2TF: a manually curated database of TF regulation in plant response to stress. *Gene* 638 36–40. 10.1016/j.gene.2017.09.067 28974472

[B389] Zhang X. Zhu Z. An F. Hao D. Li P. Song J. (2014). Jasmonate-activated MYC2 represses ETHYLENE INSENSITIVE3 activity to antagonize ethylene-promoted apical hook formation in *Arabidopsis*. *Plant Cell* 26 1105–1117. 10.1105/tpc.113.122002 24668749 PMC4001372

[B390] Zhao M. Tian Q. Zhang W. (2007). Ethylene activates a plasma membrane Ca2+-permeable channel in tobacco suspension cells. *New Phytol.* 174 507–515. 10.1111/j.1469-8137.2007.02037.x 17447907

[B391] Zhao M.-L. Wang J.-N. Shan W. Fan J.-G. Kuang J.-F. Wu K.-Q. (2013). Induction of jasmonate signalling regulators MaMYC2s and their physical interactions with MaICE1 in methyl jasmonate-induced chilling tolerance in banana fruit. *Plant. Cell Environ.* 36 30–51. 10.1111/j.1365-3040.2012.02551.x 22651394

[B392] Zhao S. Ma Q. Xu X. Li G. Hao L. (2016). Tomato jasmonic acid-deficient mutant spr2 seedling response to cadmium stress. *J. Plant Growth Regul.* 35 603–610. 10.1007/s00344-015-9563-0

[B393] Zhou J. Zhang Z. Zhang Y. Wei Y. Jiang Z. (2018). Effects of lead stress on the growth, physiology, and cellular structure of privet seedlings. *PLoS One* 13:e0191139. 10.1371/journal.pone.0191139 29494617 PMC5832220

[B394] Zhou M. Memelink J. (2016). Jasmonate-responsive transcription factors regulating plant secondary metabolism. *Biotechnol. Adv.* 34 441–449. 10.1016/j.biotechadv.2016.02.004 26876016

[B395] Zhou X. Li Q. Arita A. Sun H. Costa M. (2009). Effects of nickel, chromate, and arsenite on histone 3 lysine methylation. *Toxicol. Appl. Pharmacol.* 236 78–84. 10.1016/j.taap.2009.01.009 19371620 PMC2684878

[B396] Zhou Y. Wen Z. Zhang J. Chen X. Cui J. Xu W. (2017). Exogenous glutathione alleviates salt-induced oxidative stress in tomato seedlings by regulating glutathione metabolism, redox status, and the antioxidant system. *Sci. Hortic. (Amsterdam)* 220 90–101. 10.1016/j.scienta.2017.02.021

[B397] Zhu F. Chen M. Ye N. Shi L. Ma K. Yang J. (2017). Proteogenomic analysis reveals alternative splicing and translation as part of the abscisic acid response in *Arabidopsis* seedlings. *Plant J.* 91 518–533. 10.1111/tpj.13571 28407323

[B398] Zhu J. K. (2003). Regulation of ion homeostasis under salt stress. *Curr. Opin. Plant Biol.* 6 441–445. 10.1016/s1369-5266(03)00085-2 12972044

[B399] Zhu Z. (2014). Molecular basis for jasmonate and ethylene signal interactions in *Arabidopsis*. *J. Exp. Bot.* 65 5743–5748. 10.1093/jxb/eru349 25165148

[B400] Zhu Z. Lee B. (2015). Friends or foes: new insights in jasmonate and ethylene co-actions. *Plant Cell Physiol.* 56 414–420. 10.1093/pcp/pcu171 25435545

